# 320 GHz photonic-electronic analogue-to-digital converter (ADC) exploiting Kerr soliton microcombs

**DOI:** 10.1038/s41377-025-01778-1

**Published:** 2025-07-08

**Authors:** Dengyang Fang, Daniel Drayss, Huanfa Peng, Grigory Lihachev, Christoph Füllner, Artem Kuzmin, Pablo Marin-Palomo, Patrick Matalla, Prashanta Kharel, Rui Ning Wang, Johann Riemensberger, Mian Zhang, Jeremy Witzens, J. Christoph Scheytt, Wolfgang Freude, Sebastian Randel, Tobias J. Kippenberg, Christian Koos

**Affiliations:** 1 Institute of Photonics and Quantum Electronics (IPQ), Karlsruhe Institute of Technology (KIT), 76131 Karlsruhe, Germany; 2 Teragear GmbH, 76227 Karlsruhe, Germany; 3 Institute of Microstructure Technology (IMT), Karlsruhe Institute of Technology (KIT), 76344 Eggenstein-Leopoldshafen, Germany; 4 Deeplight GmbH, 76131 Karlsruhe, Germany | Deeplight SA, 1025 St. Sulpice VD, Switzerland; 5 Institute of Physics, Swiss Federal Institute of Technology Lausanne (EPFL), 1025 Saint-Sulpice, Switzerland; 6 Hyperlight Corporation, Cambridge, MA 02139 USA; 7 Institute of Integrated Photonics (IPH), RWTH Aachen University, 52074 Aachen, Germany; 8 Heinz Nixdorf Institute (HNI), University of Paderborn, 33102 Paderborn, Germany

**Keywords:** Microwave photonics, Frequency combs, Solitons, Fibre optics and optical communications, Integrated optics

## Abstract

Kerr soliton microcombs have the potential to disrupt a variety of applications such as ultra-high-speed optical communications, ultra-fast distance measurements, massively parallel light detection and ranging (LiDAR) or high-resolution optical spectroscopy. Similarly, ultra-broadband photonic-electronic signal processing could also benefit from chip-scale frequency comb sources that offer wideband optical emission along with ultra-low phase noise and timing jitter. However, while photonic analogue-to-digital converters (ADC) based on femtosecond lasers have been shown to overcome the jitter-related limitations of electronic oscillators, the potential of Kerr combs in photonic-electronic signal processing remains to be explored. In this work, we demonstrate a microcomb-based photonic-electronic ADC that combines a high-speed electro-optic modulator with a Kerr comb for spectrally sliced coherent detection of the generated optical waveform. The system offers a record-high acquisition bandwidth of 320 GHz, corresponding to an effective sampling rate of at least 640 GSa/s. In a proof-of-concept experiment, we demonstrate the viability of the concept by acquiring a broadband analogue data signal comprising different channels with centre frequencies between 24 GHz and 264 GHz, offering bit error ratios (BER) below widely used forward-error-correction (FEC) thresholds. To the best of our knowledge, this is the first demonstration of a microcomb-based ADC, leading to the largest acquisition bandwidth demonstrated for any ADC so far.

## Introduction

Ultra-broadband analogue-to-digital converters (ADC) are key to a variety of applications in science and industry, ranging from high-speed communications^1^ to radar and security^2^ and to capturing of ultra-short events in scientific experiments^3^. While BiCMOS or CMOS-based ADC combine compactness and robustness with the inherent scalability of the underlying technology platforms, the acquisition bandwidths of currently available devices are limited to approximately 60 GHz, and maximum sampling rates amount to 200 GSa/s^4^. The bandwidths of individual ADC can be increased by electronic time-domain or frequency-domain multiplexing, leading to real-time acquisition bandwidths^5^ of approximately 110 GHz with an effective number of bits (ENOB) of approximately 5. However, the bandwidth scalability of electronically multiplexed schemes is limited by the analogue multiplexer circuits and their jitter^8^. Photonic-electronic ADC^6–9^ may overcome some of these limitations, exploiting, e.g., pulse trains derived from ultra-stable mode-locked lasers for time-domain sampling^8,9^. Such schemes have been applied to a sampling of broadband optical waveforms^10,11^, using Kerr nonlinearities in fibres^10^, or integrated waveguides^11^, as well as to the acquisition of high-frequency electrical signals via Mach-Zehnder modulators^8^ (MZM). However, mode-locked lasers are usually bulky, which prevents integration into miniaturised systems, and they have low repetition rates of the order of a few GHz, which limits the sampling rate to, e.g., 2.5 GSa/s^8^.

In this paper, we demonstrate an ultra-broadband photonic-electronic ADC that exploits a Kerr soliton microcomb for spectrally sliced detection in the optical domain. Our scheme relies on a high-speed thin-film lithium-niobate (TFLN) electro-optic (EO) modulator that first converts the broadband analogue electrical waveform to an optical signal. The optical signal is then decomposed into spectrally sliced tributaries, which are coherently detected using the phase-locked tones of a Kerr comb as a multi-wavelength local oscillator (LO). The digital representation of the initial analogue waveform is then reconstructed from the resulting electronic tributary signals. For precise signal recovery, we develop a dedicated model of our system, which we calibrate by measuring the EO transfer function of the modulator and the optoelectronic (OE) transfer functions of the slicing filters combined with the associated in-phase/quadrature (IQ) receivers. In a proof-of-concept experiment, we demonstrate an implementation with four spectral slices and an overall acquisition bandwidth of 320 GHz in the first Nyquist band, corresponding to an effective sampling rate of at least 640 GSa/s. Our system offers ENOB values between 2.6 and 3.3, depending on the frequency of the respective test signal. We demonstrate the viability of the scheme by acquiring an ultra-broadband analogue data signal, comprising a 30 GBd 32QAM signal centred at 24.4 GHz, a 40 GBd QPSK signal centred at 233.4 GHz, and a 10 GBd 16QAM signal centred at 264.4 GHz, leading to bit-error ratios (BER) below 10^−5^. To the best of our knowledge, this is the first demonstration of a microcomb-based ADC, leading to the largest acquisition bandwidth demonstrated for any ADC so far. Moreover, our work represents the first demonstration of a TFLN Mach-Zehnder modulator (MZM) as an ultra-broadband front-end of a photonic-electronic ADC.

## Results

### Vision and concept

**Fig. 1:**
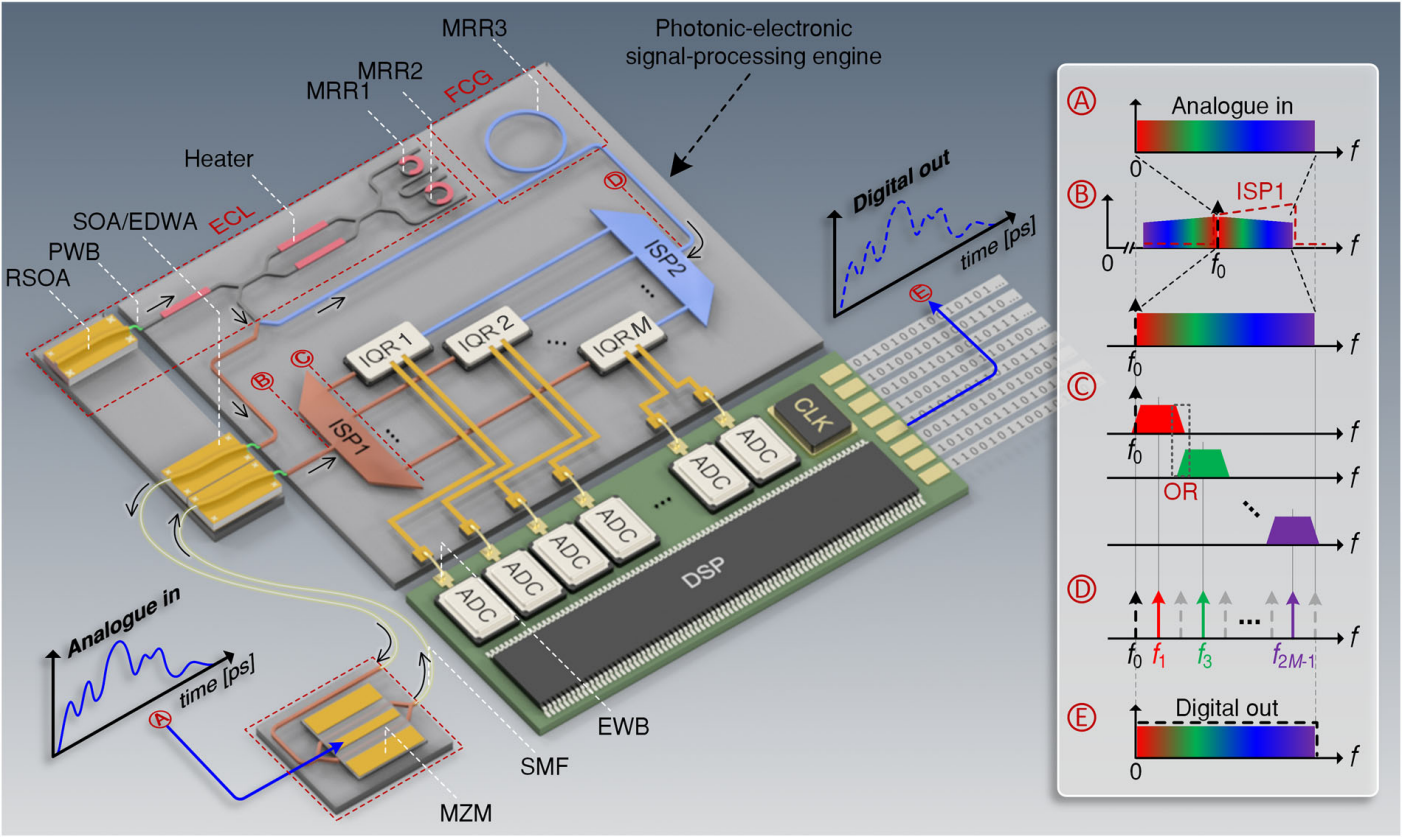
Vision of an integrated spectrally-sliced photonic-electronic ADC, relying on a photonic-electronic signal processing engine. The analogue electrical signal is translated into an optical signal by a front-end ultra-broadband electro-optic Mach-Zehnder modulator (MZM), which is fed by a continuous-wave external-cavity laser (ECL) providing an optical carrier at frequency *f*_0_. The ECL consists of a reflective semiconductor optical amplifier (RSOA) and a Sagnac loop reflector that comprises a Vernier pair of tuneable ring resonators (MRR1, MRR2) and a thermally tuneable output coupler. One portion of the ECL emission is used to pump a Kerr frequency comb generator (FCG), which comprises a high-Q Si_3_N_4_ micro-ring resonator (MRR3), and which generates a Kerr soliton comb serving as a multi-colour local oscillator (LO). The other portion of the ECL emission is amplified by a semiconductor optical amplifier (SOA) or an erbium-doped waveguide amplifier (EDWA) before being sent to the MZM. The modulated output signal of the MZM is then routed to the photonic-electronic signal-processing engine via single-mode fibres (SMF). At the input of the engine, the optical signal is amplified and sent to a first integrated spectral processor (ISP1), which extracts the upper sideband at frequencies *f* > *f*_0_, partially compensates the electro-optic response of the MZM, and then decomposes the signal into multiple spectrally sliced signal tributaries. A second integrated spectral processor (ISP2) is used to separate the multi-colour LO into individual phase-locked tones. The spectral slices of the signal are coherently detected using an array of *M* in-phase/quadrature receivers (IQR1, IQR2, …, IQR*M*), with the corresponding phase-locked comb tones serving as LO. The in-phase and quadrature components of the resulting electrical signals are digitised by an array of 2*M* synchronised low-speed electronic ADC that are connected via electric wire bonds (EWB) and then spectrally stitched together by a digital signal processor (DSP). This finally results in a digital representation of the reconstructed analogue input waveform. Inset: Illustration of the spectrally sliced photonic-electronic ADC in the spectral domain: Ⓐ Broadband analogue electrical input signal. Ⓑ The resulting optical signal is generated by modulating the broadband analogue electrical input signal onto an optical carrier prior to slicing into *M* tributaries by ISP1. Ⓒ Spectrally sliced signal tributaries with small overlap regions (OR) between neighbouring slices, generated at the output of ISP1. Ⓓ Spectrum of the frequency comb prior to slicing by ISP2. The selected tones are colour-coded according to the corresponding signal slices. Ⓔ Reconstructed spectrum of the analogue electrical input signal, obtained by merging the *M* spectral slices in the digital domain

The vision of an integrated spectrally sliced photonic-electronic ADC is illustrated in Fig. 1. The ultra-broadband analogue electrical signal (“Analogue in”) is first transferred to an optical carrier using a high-speed Mach-Zehnder modulator (MZM) that offers sufficient bandwidth of hundreds of GHz^12–14^. The modulated optical signal is then sent to an integrated photonic-electronic signal processing engine via single-mode fibres (SMF). This scheme allows for the acquisition of the ultra-broadband signal close to its source, while subsequent processing steps are done in a separate unit further away, thereby keeping the overall system architecture flexible. The narrowband optical carrier at frequency *f*_0_ is generated by a hybrid external-cavity laser^15,16^ (ECL) that is part of the signal processing engine, and that may rely on an InP-based reflective semiconductor optical amplifier (RSOA) and a low-loss silicon-nitride-(Si_3_N_4_-)based external feedback circuit, connected by an intra-cavity photonic wire bond^17^ (PWB). At the input of the signal-processing engine, the modulated optical signal is amplified, using, e.g., a low-noise semiconductor optical amplifier^18^ (SOA) or an erbium-doped waveguide amplifier^19^ (EDWA). The amplified optical signal is then sent through an integrated spectral processor (ISP1), that extracts the upper optical modulation sideband at frequencies *f* > *f*_0_, which is then decomposed into a series of *M* spectral slices with at least partially overlapping roll-off regions, see Insets Ⓐ, Ⓑ, and Ⓒ for an illustration in the spectral domain. Note that ISP1 can additionally be used to partially compensate for the electro-optic low-pass characteristics of the MZM through an appropriate optical flattening filter that suppresses spectral components close to the optical carrier at frequency *f*_0_ compared to components further away from the carrier, Inset Ⓑ. The spectral slices are then coherently detected by an array of in-phase/quadrature receivers (IQR1, …, IQR*M*) that are fed by a corresponding series of phase-locked local oscillator (LO) tones. These LO tones are derived from a chip-scale frequency-comb generator (FCG) that relies on a high-Q Kerr-nonlinear silicon-nitride micro-resonator operated in the single-soliton regime^20^, followed by a second integrated spectral processor (ISP2) that separates the comb lines. The photocurrents from the in-phase (I) and the quadrature (Q) components of the received optical signal slices are digitised by an array of 2*M* synchronised electronic ADC. A digital representation of the overall optical waveform is then reconstructed from the resulting electronic tributary signals, where the slight spectral overlap between adjacent optical slices is used for spectral stitching—similar to other optical arbitrary waveform measurement (OAWM) schemes described in previous publications^21,22^. Note that the FCG is pumped by the same ECL that also feeds the MZM, i.e., the ECL line at frequency *f*_0_ and all LO tones are phase-locked. The reconstructed optical waveform can hence be directly related to the ultra-broadband analogue electrical signal driving the MZM.

The insets of Fig. 1 illustrate colour-coded spectra of the various electrical and optical signals that occur throughout the illustrated scheme. The top row, Inset Ⓐ, shows the spectrum of the ultra-broadband analogue electrical input signal that is fed to the MZM in Point Ⓐ in the depicted scheme. The MZM produces an optical signal in Point Ⓑ in the depicted scheme, that is centred about the optical carrier frequency *f*_0_ and that features Hermitian symmetry with respect to the upper and lower sidebands, see Inset Ⓑ. These sidebands may be subject to a frequency-dependent decay in amplitude due to the low-pass characteristics of the MZM, which may be compensated by appropriately designed transmission characteristics of ISP1, see first row of Inset Ⓑ. The second row of Inset Ⓑ finally shows the optical signals after extraction and optional spectral flattening of the upper modulation sideband by ISP1. The upper sideband is then decomposed into a series of spectral slices at Point Ⓒ in the depicted scheme, see following rows of Inset Ⓒ. The LO lines selected from the FCG, Point Ⓓ of the depicted scheme, are correspondingly colour-coded in Inset Ⓓ. The resulting electronic tributary signals are digitised and finally merged by digital signal processing (DSP) to reconstruct the ultra-broadband input waveform that was fed to the MZM, Point Ⓔ and corresponding Inset Ⓔ. Note that the spectral width of each optical signal slice corresponds to twice the free spectral range *f*_FSR_ of the comb, and therefore, only every second tone positioned in the centre of a slice at frequency, ${f}_{1},{f}_{3}, \ldots, {f}_{2M-1}$, is used as an LO tone for coherent reception. This ensures gapless signal acquisition from DC to an upper frequency of $2{f}_{{\rm{FSR}}}\times M$.

### Experimental setup

**Fig. 2:**
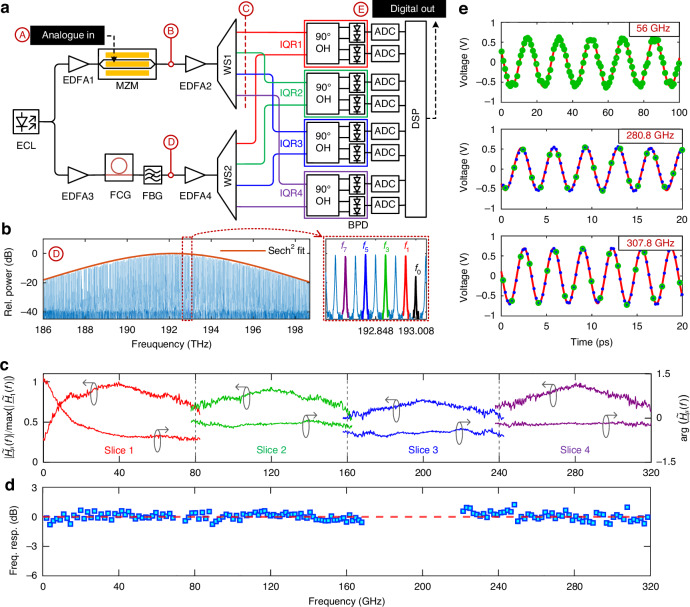
Experimental proof-of-concept demonstration of a spectrally sliced photonic-electronic ADC. **a** Simplified sketch of the experimental setup, implementing the scheme illustrated in Fig. 1 by discrete fibre-based components. A benchtop-type external-cavity laser (ECL, New Focus TLB-6700) is used to generate the carrier for optical modulation and Kerr-comb generation. Electro-optic conversion of the analogue electrical input signal (Analogue in) is accomplished by a thin-film lithium-niobate (TFLN) Mach-Zehnder Modulator (MZM)^13^, which can handle high optical powers and reach 3-dB bandwidths beyond 100 GHz, while still showing a reasonable modulation response well beyond 300 GHz^24,25^. Programmable optical filters (WS, e.g., Finisar WaveShaper 4000S) are used for slicing the resulting optical signal and the corresponding Kerr comb, which is derived from a Si_3_N_4_-based frequency comb generator (FCG, free spectral range *f*_FSR_ = 40.025 GHz). The four spectral slices are detected by an array of optical IQ receivers (IQR1,…,IQR4), which comprise 90° optical hybrids (OH, Kylia COH28) and balanced photodiodes (BPD, Finisar 43 GHz BPDV21x0R). The overall eight BPD are read out by a pair of synchronised high-speed oscilloscopes (Keysight UXR1004A), offering four channels each with a 256 GSa/s sampling rate per channel. A fibre Bragg grating (FBG) is used to suppress the remaining pump tone after the FCG, and erbium-doped fibre amplifiers (EDFA) help to overcome the optical losses in the setup. **b** Spectrum of the single-soliton Kerr comb along with a zoom-in of the selected LO tones. Note that in the proof-of-concept experiment, we detected the lower sideband of the signal spectrum due to the slightly better optical carrier-to-noise ratio (OCNR) of the comb tones, while the spectral scheme in Fig. 1 illustrates the case of detecting the upper sideband for easier understanding. **c** Amplitude and phase of the measured time-invariant frequency-dependent parts ${\underline{\tilde{H}}}_{\mu }(f)$ of the optoelectronic (OE) transfer functions associated with the $M=4$ detection channels. The transfer functions are obtained by feeding a known reference waveform (ORW) derived from an ultra-stable mode-locked laser (Menhir-1550) to Point Ⓑ of the system and by comparing the spectral amplitude and phase obtained at the various IQR outputs to that of the ORW. In combination with an independently measured MZM transfer function, this allows for full calibration of the ADC system. In the graph, the magnitude $|{\underline{\tilde{H}}}_{\mu }(f)|$ of the transfer functions has been normalised to the maximum occurring value, which was found in slice 1 at a frequency of around 40 GHz. **d** Measured frequency response of the fully calibrated photonic-electronic ADC, obtained by feeding sinusoidal analogue input signals to the MZM in Point Ⓐ, see Section S6 of the SI for details. Blue squares: Measured data points. Dashed red line: Linear fit. The frequency range between 170 GHz and 220 GHz could not be sampled due to the lack of adequate signal sources. **e** Exemplary illustrations of digitised sinusoidal test signals at frequencies of 56 GHz, 280.8 GHz, and 307.8 GHz. Green circles: Sampling points. Red curve: Least-squares fit of a sinusoidal model function. Blue dots (only in two graphs): Sinc-interpolated sampling points. The deviations of the interpolated points from the sinusoidal fits are minute, underlining the quality of the reconstructed waveforms

To demonstrate the viability of our ADC scheme, we perform a proof-of-concept experiment using a thin-film lithium-niobate (TFLN) MZM^13^ for conversion of the analogue input signal (Analogue in) and a Si_3_N_4_-based Kerr frequency comb generator^23^ (FCG, free spectral range ${f}_{{\rm{FSR}}}=40.025\,{\rm{GHz}}$) as a multi-wavelength LO, see Fig. 2 for a simplified sketch of the experimental setup. The TFLN MZM, which is biased at the zero-transmission point, can handle high optical powers and can reach 3-dB bandwidths beyond 100 GHz while still showing a reasonable modulation response well beyond 300 GHz^24,25^. For simplicity, we use discrete fibre-optic components such as programmable wave shapers (WS), 90° optical hybrids (OH), and balanced photodetectors (BPD) to implement the remaining parts of the photonic-electronic signal processing engine in our proof-of-concept experiment. For the acquisition of the resulting electrical signals, we use a pair of synchronised high-speed oscilloscopes, offering a total of 8 channels with a 256 GSa/s sampling rate each. The optical carrier for comb generation is provided by a tuneable external-cavity laser (ECL), and a narrow-band fibre Bragg grating (FBG) is used as a notch filter to suppress the remaining pump tone at the output of the Si_3_N_4_ comb generator. The same ECL provides the carrier for the MZM such that the phase noise of the signal and of the LO essentially cancel. Points Ⓐ, Ⓑ, Ⓒ, Ⓓ, and Ⓔ of Fig. 1 are also labelled in Fig. 2, and the spectral scheme shown in the insets of Fig. 1 applies accordingly. Figure 2b depicts a spectrum of our FCG (free spectral range ${f}_{{\rm{FSR}}}=40.025\,{\rm{GHz}}$) along with a zoom-in of the lines at frequencies ${f}_{1},{f}_{3},{f}_{5},{f}_{7}$ that are selected as LO-tones for coherent detection. Note that, contrary to the illustration in Fig. 1, our experiment relied on comb tones at frequencies ${f}_{1},{f}_{3},{f}_{5},{f}_{7}$ that are below the pump-tone frequency *f*_0_, thereby detecting the lower rather than the upper sideband of the modulated optical signal. This choice was made due to a slightly lower noise level of the comb tones to the left of the pump. Note that we did not yet implement an optical compensation of the frequency-dependent role-off related to the modulator in this experiment to keep the analysis of the measured data simple. Further details relating to the experimental setup are given in the Supplementary Information (SI), Fig. S1.

### System calibration and bandwidth measurement

For accurate signal reconstruction, we calibrate our setup in a three-stage process. In the first step, we measure the electro-optic (EO) transfer function of the TFLN MZM using a technique similar to the one described in Ref. ^12^, see Section S4 of the SI for the corresponding results. In the next step, we extract the complex-valued transfer functions of the associated optoelectronic signal paths between the MZM output and the electronic ADC outputs. To this end, we first separate each transfer function into two components: A time-invariant frequency-dependent part ${\tilde{\underline{H}}}_{\mu }(f)$ which describes the spectral transfer characteristics of the signal path associated with each detection $\mu ={1, ..., 4}$, and a frequency-invariant time-dependent factor ${\tilde{\underline{H}}}_{\tau ,\mu }$ which accounts for slowly varying amplitude and phase variations of the LO comb lines as well as for the random phase drift caused by the optical fibres in our setup. For a one-time calibration of the time-invariant part ${\tilde{\underline{H}}}_{\mu }(f)$, we replace the MZM at Point Ⓑ with a mode-locked laser (MENHIR-1550, repetition rate 250 MHz, pulse width 200 fs), serving as a source of a known optical reference waveform (ORW). The spectral amplitude and phase of the pulses emitted by the laser are independently measured by a frequency-resolved optical gating (FROG) technique. By referring the complex-valued received spectra at the output of the photonic-electronic signal-processing engine to the known spectrum of the ORW, we find the complex-valued time-invariant frequency-dependent part ${\tilde{\underline{H}}}_{\mu }(f)$ of the opto-electronic (OE) transfer functions associated with the slices $\mu ={1,\, ...,\, 4}$, see Fig. 2c. More details on the calibration techniques are explained in Section S5 of the SI. The thus measured transfer functions are taken into account for reconstructing the digital representation of the initial analogue electrical input signal from recorded electric tributaries. To this end, we still need to estimate the frequency-invariant time-dependent factors ${\tilde{\underline{H}}}_{\tau ,\mu }$. This is done by correcting the measured signals for the time-invariant frequency-dependent transfer functions ${\tilde{\underline{H}}}_{\mu }(f)$ first and by then comparing and merging the complex-valued amplitude spectra in the overlap regions between adjacent slices. This process is referred to as spectral stitching in the following, see Section S2 of the SI for more details. To relate the digital output to a certain voltage scale at the input, we finally record a known electrical test signal and extract the analogue-to-digital conversion factor.

We finally check the acquisition bandwidth of our fully calibrated system by feeding a small-signal sinusoidal tone to the analogue input at Point Ⓐ in Fig. 2a and by dividing the digitally recorded signal power to the physical power of the respective drive tone. This leads to the transfer function shown in Fig. 2d, confirming that the fully calibrated system offers an essentially flat response up to 320 GHz.

### Acquisition of sinusoidal test signals

To prove the viability of our concept, we perform two sets of experiments. In the first set of experiments, we generate electrical test signals with frequencies of 56.0 GHz, 280.8 GHz, and 307.8 GHz using different signal generators, feed them to the analogue input of our system, and record the associated digital signal, see Fig. 2e. To investigate the quality of the measured signals, we perform a sinc-interpolation of the sampling points (green circles/blue dots) and we fit them by a sinusoidal model function (red line) to approximate the associated continuous-time waveforms. The deviations of the interpolated points from the sinusoidal fits are minute, underlining the quality of the reconstructed waveforms. Note that we always reconstructed all four slices in these experiments, even if the test signal was only present on one or two slices. To facilitate spectral stitching in case the spectral overlap region between neighbouring slices does not contain any components of the use signal, we added artificial stitching tones in the optical domain, see Fig. S6 of the SI for the spectrum of an exemplary constructed signal. Note also that our system operates within the first Nyquist band—unlike other previously demonstrated photonic ADC systems, which relied on down-sampling of single-frequency tones in higher-order Nyquist bands^6,8^. Offering the full bandwidth of 320 GHz in the first Nyquist band is key to the acquisition of broadband analogue test signals, as discussed in the next section.

### Acquisition of broadband analogue signals

**Fig. 3:**
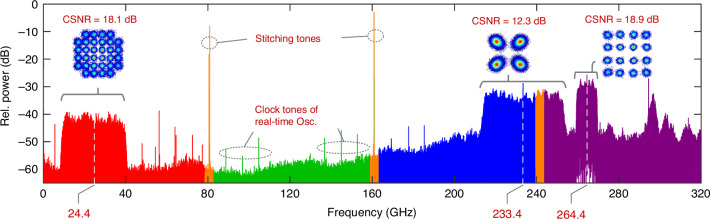
Digital representation of a broadband electronic signal. The analogue signal covers the spectral ranges 0 GHz…80 GHz, 160 GHz…240 GHz, and 240 GHz…320 GHz. The spectral region 80 GHz…160 GHz is empty. The electronic signal comprises three QAM data signals, centred at carrier frequencies 24.4 GHz (30 GBd 32QAM), 233.4 GHz (40 GBd QPSK), and 264.4 GHz (10 GBd 16QAM). From the digital representation indicated by horizontal braces, three constellation diagrams can be extracted. In the “empty” region, clock tones from the real-time sampling oscilloscope are to be seen. In the middle of the empty overlap regions, stitching tones (orange) help to adjust the phases of neighbouring slices

In a second experiment, we use our photonic-electronic ADC to acquire a broadband analogue data signal, consisting of a 30 GBd 32QAM waveform centred at 24.4 GHz, a 40 GBd QPSK waveform centred at 233.4 GHz, and a 10 GBd 16QAM waveform centred at 264.4 GHz. Figure 3 shows the spectrum of the reconstructed signal along with the constellation diagrams of the data signals extracted by using state-of-the-art digital signal processing (DSP) techniques. The constellation signal-to-noise ratios (CSNR) of the 30 GBd, the 40 GBd, and the 10 GBd data signals amount to 18.1 dB, 12.3 dB, and 18.9 dB, corresponding to error-vector magnitudes (EVM) of 12.5%, 24.3%, and 11.3%, respectively. The underlying signal impairments can be attributed to two main effects: First, the various optoelectronic devices that were used for generating and converting the test signal were subject to significant nonidealities. This applies in particular to the TFLN MZM, which suffers from a strong spectral dip in the transfer function at approximately 290 GHz, see Section S4 of the SI, as well as to a uni-travelling carrier photodiode (UTC-PD), that was used to generate high-speed test signals by photomixing, see Section S8 of the SI. In this context, the distortion related to the UTC-PD should be attributed to the signal source rather than to the photonic-electronic ADC system. Second, this experiment was performed with a rather low optical carrier-to-noise ratio (OCNR) of the Kerr-comb tones, which leads to multiplicative noise as seen in the constellation diagrams, see Sections S8 and S9.1 of the SI for a more detailed discussion. Still, the bit error ratios (BER) of the reconstructed individual signals are below 10^−5^, thereby proving the viability of the scheme. The increase of the noise floor towards higher frequencies and the noise peak at 290 GHz are a consequence of the digital compensation of the MZM EO transfer function, which decreases with frequency and exhibits the above-mentioned dip at around 290 GHz, see Fig. S3b of the SI for details. Note that coupling of this ultra-broadband signal to the MZM electrode requires special measures, see Section S8 of the SI explains the details. Besides the data signals, the spectrum contains residual clock tones of the oscilloscope as well as optical stitching tones at approximately 80 GHz and 160 GHz, which were included to enable proper spectral stitching of the otherwise empty spectral overlap regions. Note that the stitching tones were chosen to be unnecessarily high in this specific experiment and could be reduced by approximately 30 dB, see Fig. S6 of the SI.

### ENOB estimation

We also estimate the ENOB of our system by measuring the signal-to-noise-and-distortion ratio (SINAD) of a harmonic test signal that covers the full-scale input range (FSIR) of our ADC. To determine the FSIR, we have to account for the nonlinear transfer characteristics of the MZM, which lead to a strong increase of third-order and residual second-order distortions at high input swings, while the SINAD is limited by the ASE noise of the EDFA following the MZM (EDFA2 in Fig. 2a) at low signal swings. In between, we find an optimum voltage swing of the RF input signal, which leads to maximum SINAD. We extract this optimum voltage swing for a low-frequency test signal, for which the nonlinear distortions of the modulator are most pronounced, see Section S7.1 of the SI for details, and we define it as the FSIR of our ADC. In a next step, we estimate the SINAD values associated with a full-scale test signal at different frequencies. The associated dB-values of the SINAD, SINAD_dB_ = 10log_10_(SINAD), are then used to derive the effective number of bits (ENOB) using the relation ENOB = (SINAD_dB_ − 1.76)/6.02 according to the corresponding IEEE definition^26^. Using the current implementation of our system with sinusoidal test signals at 2.0 GHz, 5.6 GHz, 9.2 GHz, 280.0 GHz, and 307.8 GHz, we find ENOB values of 2.7, 2.6, 2.6, 3.1, and 3.3, respectively. Note that the ENOB becomes better for higher test-signal frequencies, since the nonlinear distortions of the MZM are less pronounced due to higher RF losses in the on-chip transmission lines and since certain intermodulation products fall outside the frequency domain of interest, see Section S7.2 of SI for details.

## Discussion

### Performance and limitations

While our proof-of-concept implementation of a photonic-electronic ADC shows extraordinary performance advantages, in particular with respect to the acquisition bandwidth, it is clearly also subject to limitations, some of which are fundamental in nature whereas others can be overcome by technical improvements. With respect to acquisition bandwidth, the main limitation of the current implementation lies in the roll-off of the MZM transfer function as well as in the spectral dip at approximately 290 GHz. In contrast to that, the bandwidth of the photonic-electronic signal-processing engine can be efficiently scaled beyond the currently demonstrated 320 GHz by using more IQ receivers (IQR) and by exploiting the fact that Kerr comb sources can easily provide tens of comb lines^27^. The limited modulator bandwidth and the spectral dip in the transfer function can be avoided by optimised device designs^25^ or by using alternative modulator technologies^12,28,29^, see Section 4 of the SI for a more detailed discussion. We analyse the impact of these effects in Section S7.3 of the SI and estimate that the ENOB can be improved to approximately 3.6 by avoiding the spectral dip in the MZM transfer function and by compensating the roll-off of the MZM in the optical domain.

Another important limitation of the current implementation is the optical carrier-to-noise ratio (OCNR) of the underlying Kerr-comb tones. More specifically, the OCNR of the comb tones used in the ENOB estimation above are in the range between 28.6 dB and 30.5 dB, dictated by the ASE noise of the booster EDFA (EDFA4 in Fig. 2a) in combination with the comb-line power found at the output of the FCG. These OCNR levels can be significantly increased to 40 … 50 dB by increasing the per-line comb power, e.g., through advanced microcomb designs^30,31^, possibly in combination with using dark solitons in normal-dispersion regime^32,33^, see Section S9.1 of the SI for a more detailed discussion of comb OCNR.

In addition to the comb OCNR, the ENOB of the underlying electronic ADC is a limiting factor for the overall performance of our current ADC system. For the high-speed real-time oscilloscopes used in our experiment (Keysight UXR series), the manufacturer specifies an ENOB of approximately 5 for a 100 GHz acquisition bandwidth, which, in combination with the roll-off of the MZM transfer function, limits the SINAD levels achieved in our experiments, see Sections S4 and S7.1 of the SI for a more detailed discussion. This limitation may be overcome by using more detection channels and, hence, smaller detection bandwidth per channel, which allows the use of electronic ADC with higher ENOB^22,34^.

Another important performance limitation of high-speed ADC, in general, is related to timing uncertainties, which eventually restrict bandwidth scalability. In this respect, using a Kerr comb as a multi-wavelength LO turns out to be particularly attractive since Kerr combs can significantly mitigate jitter-related distortions of the overall acquisition system^7^. Specifically, while the high-end oscilloscopes (Keysight UXR series) used in our experiment are subject to a clock timing jitter of ${\sigma }_{\text{ADC}}$ = 25 fs, the root-mean-square (RMS) timing jitter of our LO comb is significantly lower and amounts to ${\sigma }_{\text{LO}}$ = 3.1 fs only. The low timing jitter of the comb allows for precise stitching of frequency bands with minimum impairments and is hence key to the outstanding bandwidth-scalability of our approach, see in Section S11 of the SI for a more detailed analysis. Kerr-comb-based photonic-electronic ADC can hence pave a path towards overcoming the jitter limitations of all-electronic ADC systems.

Other limitations of the proposed photonic-electronic ADC scheme arise from the technical complexity of the approach, regarding both the implementation and the operation of the system. It should be mentioned that the unprecedented acquisition bandwidth of our approach comes at the price of hardware efforts and power consumption that may be higher than that of conventional monolithically integrated CMOS-based ADC. We provide a more detailed analysis of this aspect in Section S10 of the SI, using the so-called Schreier figure of merit^35^ (FOM) as a performance metric. For a 320 GHz photonic-electronic ADC system that implements our approach based on components that are available already today, we estimate a Schreier FOM of 140.4 dBJ^−1^. This compares well to all-electronic high-speed ADC, which offer Schreier FOM in the range between 130 dBJ^−1^ and 150 dBJ^−1^, while acquisition bandwidths are limited to below 100 GHz, see Fig. S11 of the SI.

Another important aspect for real-world application of the proposed photonic-electronic ADC scheme is the computational complexity of the DSP-based signal reconstruction. Notably, our signal-reconstruction scheme only involves fundamental DSP functions such as fast Fourier transformations (FFT), digital filtering in the frequency domain, and inverse fast Fourier transformations (IFFT), see Section S12 of the SI. All of these functions are routinely used in high-speed coherent optical communication systems, typically implemented on application-specific integrated circuits (ASIC) that are well suited for real-time processing, such that implementation in a compact photonic-electronic ADC should not be a fundamental problem. In Section S12 of the SI, we support this notion by quantifying the computational complexity of our signal reconstruction scheme and by comparing it to the computational power of state-of-the-art communication ASIC and field-programmable gate arrays (FPGA).

### Application potential

**Fig. 4:**
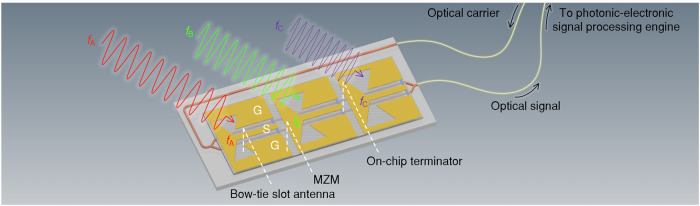
Conceptual illustration of an ultra-broadband analogue photonic-electronic front end. The front end relies on a single Mach-Zehnder modulator (MZM) for simultaneous readout of an array of bow-tie slot antennas. Within this array, each antenna is optimised for a specific frequency band (centre frequencies *f*_A_, *f*_B_, *f*_C_) and coupled to the ground-signal-ground (GSG) coplanar transmission line of the MZM, thus allowing for efficient multiplexing of various received millimetre-wave signals on the same optical carrier. The resulting optical signal is transmitted through optical fibres and further processed in the photonic-electronic signal processing engine, see Fig. 1 above. Assuming an MZM with sufficient bandwidth, the illustrated concept allows for efficient acquisition of ultra-broadband signals with overall bandwidths of hundreds of GHz. In comparison to conventional millimetre-wave receivers, the illustrated concept replaces bulky and costly analogue components such as RF filters, intermediate frequency (IF) amplifiers, and RF mixers with a compact and robust fibre-coupled photonic-electronic front-end converter

Combining cutting edge photonic integrated circuits (PIC) technology with state-of-the-art microelectronics, we believe that ultra-broadband photonic-electronic ADC can open vast application potential in many fields of industry and science. One such field is ultra-wideband radio communications, e.g., in sixth-generation (6G) wireless networks. In this context, photonic-electronic ADC could allow for direct processing of RF and millimetre-wave signals in the digital domain, offering not only unprecedented processing bandwidth, but also replacing bulky and costly analogue components such as RF filters, intermediate frequency (IF) amplifiers, and RF mixers by a compact and robust fibre-coupled photonic-electronic front-end converter. Figure 4 shows a conceptual illustration of such a front-end, leveraging a single MZM for parallel readout of an array of bow-tie slot antennas. Within this array, each antenna is optimised for a specific frequency band (centre frequencies *f*_A_, *f*_B_, *f*_C_) and coupled to the ground-signal-ground (GSG) coplanar transmission line of the MZM, thus allowing for efficient multiplexing of various received millimetre-wave signals on the same optical carrier. The resulting optical signal is transmitted through optical fibres, offering much larger bandwidth and lower loss than RF cables, and finally further processed in the integrated photonic-electronic signal processing engine, see Fig. 1 above. Assuming an MZM with sufficient bandwidth, such a concept would allow for efficient acquisition of ultra-broadband signals with spectra spread out over a large frequency range of hundreds of GHz. Note that the acquisition band of the proposed photonic-electronic ADC scheme can be easily frequency-shifted by using higher-order LO comb tones that have a larger offset to the optical carrier *f*_0_. Having direct digital access to hundreds of gigahertz of coherent ADC bandwidth will also open entirely new prospects for software-defined radio communications, allowing for instantaneous switching between different frequency bands and communication protocols and enabling highly dynamic allocation of spectral resources. Similarly, advanced radar systems could benefit from ultra-large ADC bandwidths to offer improved detection accuracy and better spatial resolution, e.g., for detecting small objects.

Another application field is high-speed test and measurement equipment. Notably, the impressive evolution of CMOS technology on the newest 5 nm and 3 nm technology nodes has led to significant bandwidth increases of commercially available electronic integrated circuits. Examples comprise, e.g., application-specific integrated circuits (ASIC) for signal-processing in optical transceivers, for which symbol rates beyond 200 GBd are being discussed^36^. Testing and verification of such ASIC will require measurement equipment that clearly exceeds the performance of the respective device under test. This is becoming increasingly difficult, not only for technical reasons but also from an economic perspective. Specifically, while advanced ASIC for high-speed communications have large market volumes and hence justify the costly use of most advanced semiconductor technology nodes, the market of test and measurement equipment is rather limited and has to rely on older, less expensive nodes. As a consequence, state-of-the-art high-speed oscilloscopes from leading vendors still rely on ADC chips, which are implemented in rather old 65 nm CMOS nodes and are combined with multi-stage front-end samplers that leverage InP-based high-electron-mobility transistor (HEMT) technology^5^. This leads to very complex system architectures with limited potential for scaling the bandwidth. Our approach paves a path towards overcoming these limitations by leveraging photonic integrated circuits along with optical frequency combs for simplified bandwidth scaling, while simultaneously reducing timing jitter and associated signal distortions, see Section S11 of the SI for a more detailed discussion. We believe this offers a compelling solution for next-generation test and measurement systems—not only for signal detection but also for signal generation^37^.

### Summary

In summary, we demonstrate a photonic-electronic ADC with a record-high acquisition bandwidth of 320 GHz, corresponding to an effective sampling rate of at least 640 GSa/s. The scheme relies on a high-speed electro-optic (EO) modulator to convert the analogue broadband electrical waveform to an optical signal. While the compact EO modulator can be placed very close to the source signal, the converted optical signal can be transmitted through a fibre to the signal processing engine further away, avoiding the signal loss typically associated with long RF cables in conventional ADC systems. In the processing engine, the optical signal is first decomposed into spectrally sliced tributaries, which are then coherently detected using a Kerr comb with phase-locked tones serving as a multi-wavelength local oscillator (LO), and the digital representation of the initial analogue waveform is reconstructed from the resulting electronic tributary signals. For precise signal reconstruction, we develop a quantitative system model that is calibrated by measuring the EO transfer function of the modulator and the optoelectronic (OE) transfer functions of the various detection paths. Our system offers ENOB values between 2.6 and 3.3, depending on the frequency of the respective test signal. We demonstrate the viability of the scheme by acquiring an ultra-broadband analogue data signal, comprising a 30 GBd 32QAM signal centred at 24.4 GHz, a 40 GBd QPSK signal centred at 233.4 GHz, and a 10 GBd 16QAM signal centred at 264.4 GHz, leading to bit-error ratios (BER) below 10^−5^. To the best of our knowledge, this is the first demonstration of a microcomb-based ADC, leading to the largest acquisition bandwidth demonstrated for any ADC so far. By increasing the number of slices and by exploiting highly stable optical frequency combs with ultra-low phase noise^38^, our concept may open a path to acquisition bandwidths of the order of 1 THz using a fully integrated photonic-electronic system^22^. We believe that our work shows the disruptive potential of Kerr frequency combs, complementing a series of earlier demonstrations by various groups, e.g., in the fields of high-speed optical communications^39,40^, ultra-fast distance measurements^41^, massively parallel light detection and ranging (LiDAR)^42^ or high-resolution optical spectroscopy^43^.

## Materials and methods

A complete and detailed description of the materials and methods supporting the main conclusions of this paper is provided in the attached Supplementary Information (SI).

## Supplementary information

Supplementary Information

## Data Availability

The data that support the findings of this study may be obtained from the corresponding authors upon request.
