# Supplementary material for: 320 GHz photonic-electronic analogue-to-digital converter (ADC) exploiting Kerr soliton microcombs
Source: Light Sci Appl. 2025 Jul 8;14:241. doi: 10.1038/s41377-025-01778-1 (PMC12238241; doi:10.1038/s41377-025-01778-1)
Supplement: Supplementary file 1 — Supplementary Information [file 41377_2025_1778_MOESM1_ESM.pdf]

Supplementary Information for “320 GHz Photonic-Electronic Analogue-to-Digital Converter (ADC) Exploiting Kerr Soliton Microcombs”

Dengyang Fang[1,2†*], Daniel Drayss[1,2,3†], Huanfa Peng[1,4], Grigory Lihachev[5], Christoph Füllner[1], Artem Kuzmin[3], Pablo Marin-Palomo[1], Patrick Matalla[1], Prashanta Kharel[6], Rui Ning Wang[5], Johann Riemensberger[5], Mian Zhang[6], Jeremy Witzens[7], J. Christoph Scheytt[8], Wolfgang Freude[1], Sebastian Randel[1], Tobias J. Kippenberg[4,5], Christian Koos[1,2,3,4*]

[1]*Institute of Photonics and Quantum Electronics (IPQ), Karlsruhe Institute of Technology (KIT), 76131 Karlsruhe, Germany*
[2]*Teragear GmbH, Seboldstrasse 22, 76227 Karlsruhe, Germany*
[3]*Institute of Microstructure Technology (IMT), Karlsruhe Institute of Technology (KIT), 76344 Eggenstein-Leopoldshafen, Germany*
[4]*Deeplight GmbH, 76131 Karlsruhe, Germany | Deeplight SA, 1025 St. Sulpice VD, Switzerland*
[5]*Institute of Physics, Swiss Federal Institute of Technology Lausanne (EPFL), 1015 Lausanne, Switzerland*
[6]*Hyperlight Corporation, Cambridge, MA, 02139, USA*
[7]*Institute of Integrated Photonics (IPH), RWTH Aachen University, 52074 Aachen, Germany*
[8]*Heinz Nixdorf Institute (HNI), University of Paderborn, 33102 Paderborn, Germany*
†*These authors contributed equally: D. Fang, D. Drayss*
**e-mail:* dengyang.fang@kit.edu, christian.koos@kit.edu

Table of Contents

S1. Experimental setup 3
S2. System model and signal reconstruction 4
S2.1 Signal and system model 4
S2.2 Signal reconstruction and compensation of phase drifts 9
S3. Calibration of the in-phase/quadrature (IQ) receiver 13
S4. Calibration of the Mach-Zehnder modulator (MZM) electro-optic (EO) response 14
S5. Calibration of the system opto-electronic (OE) response 16
S6. Frequency response of the photonic-electronic ADC 18
S7. Effective number of bits (ENOB) analysis of the photonic-electronic ADC 19
S7.1 Full-scale input range (FSIR) 19
S7.2 Signal-to-noise-and-distortion ratio (SINAD) and effective number of bits (ENOB) 20
S7.3 Further improvement in ENOB 23
S8. Acquisition of broadband data signals 24
S9. Characteristic of Kerr soliton microcomb 25
S9.1 Optical carrier-to-noise ratio (OCNR) 25
S9.2 Phase noise and timing jitter 27
S10. Power consumption of spectrally sliced photonic-electronic ADC 28
S11. Impact of electronic and Kerr-comb-related timing jitter 30
S12. Computational complexity of signal reconstruction 32
S12.1 Estimation of computational complexity 32
S12.2 Comparison to state-of-the-art ASIC performance 35
S12.3 Comparison to state-of-the-art FPGA performance 36
S13. References 38

This document provides supplementary information to manuscript "320 GHz Photonic-Electronic Analogue-to-Digital Converter (ADC) Exploiting Kerr Soliton Microcombs", containing technical details of the experimental setups, an explanation of the underlying system models and calibration techniques, as well as additional explanations related to signal reconstruction and analysis of measurement data.

S1. Experimental setup

The simplified experimental setup is depicted in Fig. S1. The analogue electrical signal (Analogue in) is translated to the optical domain by a high-speed thin-film lithium-niobate Mach-Zehnder modulator (MZM, Hyperlight Corp., MA, USA) [1]. The optical carrier with frequency f_0 is provided by a tuneable external-cavity laser (ECL, TLB-6700, Newport Corp., CA, USA). The MZM operates at the zero-transmission point such that the carrier should be fully suppressed. However, due to the limited extinction ratio of the MZM of 23 dB, we still observe a residual carrier at the output, which is further suppressed by a narrow-band fibre Bragg grating (FBG, AOS GmbH, Dresden, Germany) acting as a notch filter after point Ⓐ in Fig. S1a. As illustrated in Inset Ⓐ of Fig. S1b, the optical upper and lower sidebands of the generated signal spectrum are Hermitian conjugates and contain the same information, such that only one sideband needs to be considered further. In the current experimental implementation, we choose the lower sideband. This choice was motivated by the fact that the associated local-oscillator (LO) tones, that are used for coherent detection of the signal later on, show lower noise levels as compared to the tones corresponding to the upper sideband. However, for a more intuitive understanding of the scheme, we will discuss the system model and signal reconstruction in the following Section S2 based on detection of the upper-sideband signal as illustrated in Fig. S1b. An erbium-doped fibre amplifier (EDFA1) after the FBG compensates the modulator loss. The unnecessary upper

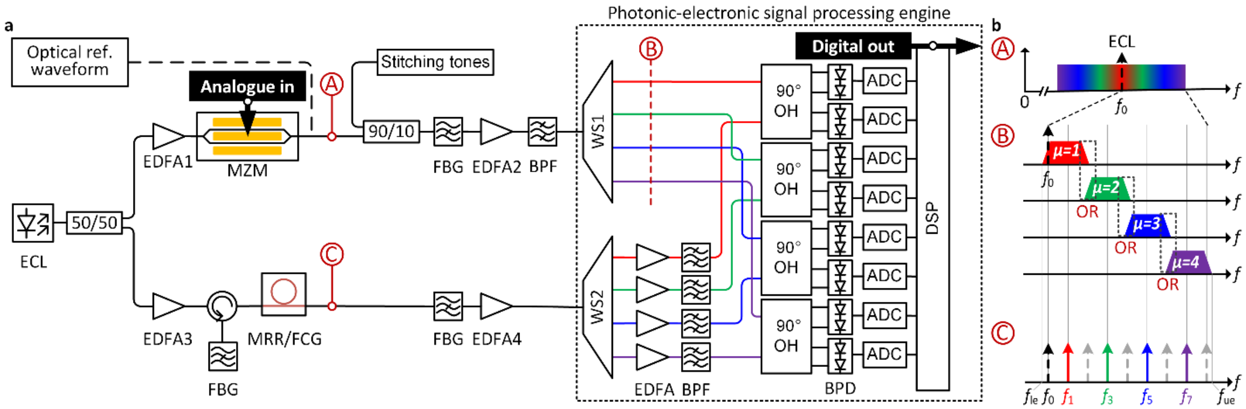

Fig. S1| Simplified experimental setup of an ultra-broadband spectrally-sliced photonic-electronic ADC system. a An external-cavity laser (ECL) emitting a narrowband tone at frequency f_0 provides an optical carrier, which is modulated by a broadband electric signal (Analogue In) via a high-speed Mach-Zehnder modulator (MZM) operated at its zero-transmission point. A copy of the carrier is amplified and serves as a pump tone for a high-Q silicon-nitride (Si_3N_4) microring resonator (MRR), which acts as frequency comb generator (FCG) with a free spectral range $f_{\mathrm{FSR}} = 40.025\ \mathrm{GHz}$. After the MZM, a fibre Bragg grating (FBG) helps to suppressing the residual carrier at frequency f_0 within the optical signal. A programmable optical filter (WS1, Finisar WaveShaper) is used to slice the signal spectrum into four tributaries. An array of coherent receivers follows, each containing a 90° optical hybrid (OH) and a pair of balanced photodiodes (BPD). At the output of the FCG, the residual pump tone is suppressed by another FBG, and a second optical filter (WS2) separates the comb lines, which act as local-oscillator (LO) tones for the coherent receivers. The BPD output signals are digitized by an array of eight ADC, which are part of two synchronized real-time oscilloscopes (Keysight UXR 1004A). Digital signal processing (DSP) techniques are used to extract the digital representation (Digital out) of the analogue input signal (Analog in). **b** Illustrations of optical spectra. Inset Ⓐ: Broadband optical spectrum at Point Ⓐ of the experimental setup. Inset Ⓑ: Four optical spectral slices numbered $\mu = 1, \ldots, 4$ at Point Ⓑ, featuring the desired overlap regions (OR) which are associated with the roll-off of the four spectral slices. The lower edge of the overall detection range is associated with optical frequency f_{le}, whereas f_{ue} refers to the optical frequency associated with the upper edge. Note that the roll-off at the lower edge of the first and the high-frequency edge of the last slice also allows to detect spectral signal components at frequencies $f \in [f_{\mathrm{le}}, f_0]$ slightly below the carrier frequency f_0. Inset Ⓒ: FCG output spectrum at Point Ⓒ. Note that WS2 suppresses all even-numbered comb lines and extracts only the odd-numbered frequencies f_1, f_3, f_5, f_7 centred at the spectral slices $\mu = 1, \ldots, 4$.

sideband and any out-of-band amplified spontaneous emission (ASE) noise from EDFA1 are suppressed by a bandpass filter (BPF, Koshin Kogaku Co. Ltd., Kanagawa, Japan). This avoids overloading the subsequent demultiplexer (WS1, WaveShaper 4000S, Finisar Corp., CA, USA), which decomposes the lower sideband of the optical signal into $M = 4$ spectral slices. To this end, the different channels of WS1 are programmed to act as optical bandpass filters with flat-top transmission, steep roll-off and small overlap regions (OR) between neighbouring slices, as illustrated Inset Ⓑ of Fig. S1b. The redundant information contained in the OR will be exploited later on to facilitate spectral stitching, see Section S2.2 . In case the OR do not contain any components of the use signal, artificial optical stitching tones provided by a bank of ECL (N7714A, Keysight Technologies Inc., CA, USA) can be added by a 90/10 coupler after point Ⓐ. The spectral slices are then routed to four in-phase / quadrature (IQ) receivers for coherent detection. Each IQ receiver comprises a 90° optical hybrid (OH, COH28, Kylia) and a pair of balanced photodiodes (BPD, BPDV21x0R, 43 GHz bandwidth, Finisar Corp.). The resulting photocurrents from the I and Q components of the received optical signal slices are digitized by two synchronized real-time oscilloscopes (UXR 1004A, Keysight Technologies Inc., CA, USA), which offer a total of eight acquisition channels with a sampling rate of 256 GSa/s each.

The local oscillator (LO) comb is derived from a chip-scale optical frequency comb generator (FCG), relying on a silicon-nitride (Si_3N_4) microring resonator (MRR, $f_{FSR} = 40.025$ GHz). The FCG is pumped by the same ECL which provides the carrier for the MZM such that the phase noise of the signal and of the LO tones is identical and essentially cancels during coherent detection. The pump tone is amplified by an EDFA and a combination of a circulator and a FBG acts as a narrow-band optical bandpass filter to remove ASE noise from the pump tone before the FCG to avoid degradation of the optical carrier-to-noise ratio (OCNR) of the generated comb lines. At the FCG output, another FBG notch filter suppresses the residual pump tone before the signal is fed into another EDFA (EDFA4). The amplified comb spectrum is fed into a second demultiplexer (WS2), which selects the odd-numbered comb lines with frequencies f_1, f_3, f_5, f_7 as LO-tones for coherent detection in the various IQ receivers. Note that selecting only the odd-numbered comb lines as LO tones allows for using identical minimum-bandwidth receivers to efficiently cover the full spectral range of the electrical input signal from DC to a maximum frequency of $2Mf_{FSR}$. For each of the selected comb lines, an EDFA is used to compensate the insertion loss of WS2. The spectrum at Point Ⓒ is illustrated in Inset Ⓒ of Fig. S1b, where the broken spectral lines indicate the even-numbered comb tones, which are later suppressed by WS2. To ensure the desired overlap between neighbouring signal slices, the spectral width of these slices has to be slightly larger than twice the free spectral range f_{FSR} of the LO comb.

S2. System model and signal reconstruction

In this section, we describe the mathematical model as well as signal reconstruction for the proposed photonic-electronic ADC system. Throughout this section, we use lowercase letters $\underline{\psi}(t)$ to denote transfer functions or signals with carrier frequencies in the optical domain, i.e. at hundreds of THz. The underscore indicates that we assume a complex-valued signal in the optical domain – usually an analytic signal with a single-sided power spectrum. The associated complex-valued envelopes in the baseband are denoted by uppercase letters $\underline{\Psi}(t)$, which, in some cases, may be written as a real-valued quantity $\Psi(t)$ multiplied by an exponential phase factor, e.g., Eq. (S1) below. The same notation of upper-case letters without underscore is applied to natively real-valued RF signals such as the analogue input signal $U_S(t)$. For complex-valued time-domain signals, e.g., $\underline{\psi}(t)$, Fourier transforms are indicated with both a tilde and an underscore $\underline{\tilde{\psi}}(f)$, while for real-valued time-domain signals, e.g., $\Psi(t)$, the Fourier transform $\tilde{\Psi}(f)$ will not have an underscore.

S2.1. Signal and system model

Signal reconstruction in our photonic-electronic ADC relies on precise mathematical models for the system and the various signals, which shall be derived based on the illustration of the underlying concept in Fig S2. We start from the broadband analogue waveform that is fed to the MZM and that is represented by a real-valued electric signal $U_S(t)$, having a double-sided spectrum with the spectra for positive and negative frequencies being the complex conjugates of one another. This signal is applied to the electrodes of the MZM and modulates an optical carrier at frequency f_0. This leads to a modulated optical waveform, which can be represented by a complex-valued analytic signal,

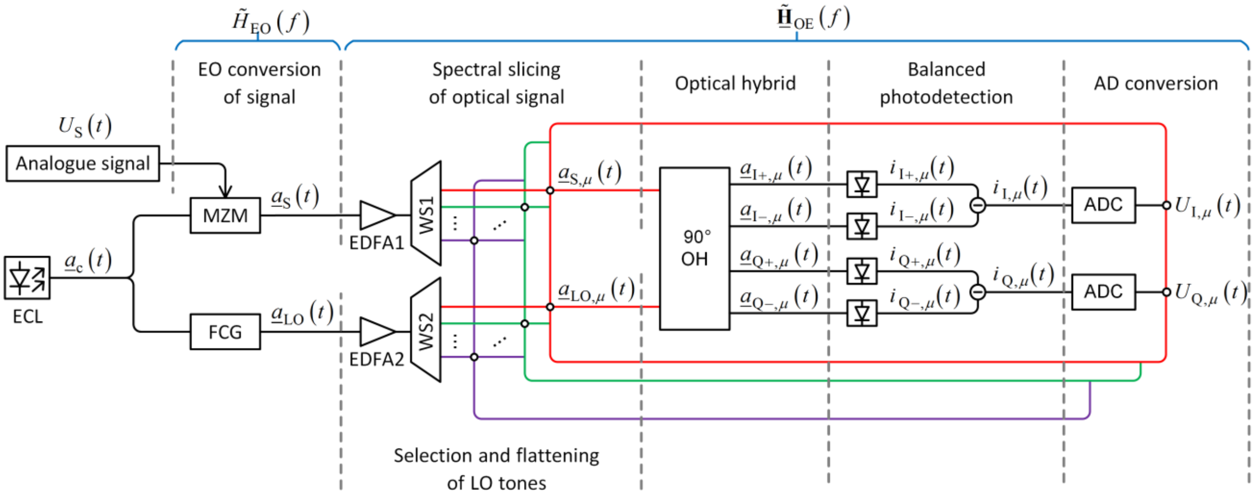

Fig. S2| System model of the spectrally sliced photonic-electronic ADC. The analogue signal $U_S(t)$ is converted to the optical domain by a Mach-Zehnder modulator (MZM) featuring an EO transfer function $\tilde{H}_{EO}(f)$. The optical carrier is provided by an external-cavity laser (ECL) emitting a carrier $\underline{a}_c(t)$. The resulting modulated optical signal $\underline{a}_S(t)$ is first amplified by an erbium-doped fiber amplifier (EDFA1) and then decomposed into M spectrally-sliced signal tributaries $\underline{a}_{S,\mu}(t), \mu = 1, \ldots, M$ by an optical filter, which may be implemented as a wave shaper (WS1). The signal tributaries are detected by an array of coherent receivers, each comprising a 90° optical hybrid (90° OH) and a pair of balanced photodetectors, using a corresponding set of LO tones $\underline{a}_{LO,\mu}(t), \mu = 1 \ldots M$, derived from an optical frequency comb $\underline{a}_{comb}(t)$, generated by a frequency comb generator (FCG). The in-phase (I) and quadrature (Q) components of the resulting electric tributary signals are digitized by an array of 2M electric ADC. The signal associated with each spectral slice $\underline{a}_{S,\mu}(t)$ propagates through waveguides, amplifiers, as well as optical filters and IQ receivers and is finally acquired by an electronic ADC. The transfer function of all these this signal paths can finally be merged into a column matrix of OE transfer functions $\underline{\tilde{\mathbf{H}}}_{OE}(f)$, see Eq. (S20).

$$\underline{a}_S(t) = \underline{A}_S(t) \cdot e^{j2\pi f_0 t} = A_S(t) \cdot e^{j(2\pi f_0 t + \varphi_0)} \tag{S1}$$

where the envelope $A_S(t)$ depends on the applied drive signal $U_S(t)$ and where φ_0 represents the phase of the optical carrier. The Fourier transform of the analytic time-domain signal is denoted by a tilde on top of the corresponding symbol,

$$\underline{\tilde{a}}_S(f) = \int_{-\infty}^{+\infty} \underline{a}_S(t) e^{-j2\pi f t} dt = \tilde{A}_S(f - f_0) e^{j\varphi_0} \tag{S2}$$

For simplicity, we assume the MZM to be perfectly balanced and operated in push-pull configuration with a bias at its zero-transmission point. We further assume that the amplitude of the applied drive voltage $U_S(t)$ is well below the half-wave voltage U_π of the device, leading to a linear relationship between the applied RF signal and the envelope of the modulated optical carrier, which can be expressed by means of a time-domain convolution with an electro-optic impulse response $H_{EO}(t)$ of the MZM,

$$A_S(t) = H_{EO}(t) * U_S(t) \tag{S3}$$

Note that $A_S(t)$, $H_{EO}(t)$, and $U_S(t)$ are all real-valued time-domain quantities. In the frequency domain, the convolution according to Eq. (S3) can be replaced by a product, such that the spectrum $\underline{\tilde{a}}_S(f)$ of the analytic optical signal can be re-written as

$$\begin{aligned} \underline{\tilde{a}}_S(f) &= \tilde{A}_S(f - f_0) e^{j\varphi_0} \\ &= \tilde{H}_{EO}(f - f_0) \tilde{U}_S(f - f_0) e^{j\varphi_0} \end{aligned} \tag{S4}$$

Models for the electro-optic (EO) transfer function $\tilde{H}_{\text{EO}}(f)$ of different MZM implementations can be found in the literature [2][3][5].

Once the RF input signal is translated to the optical domain, we use a comb-based optical arbitrary waveform measurement (OAWM) technique to reconstruct the slowly-varying envelope $A_{\text{S}}(t)$ of the optical signal [11]. To this end, either the upper or the lower sideband of the optical signal is first decomposed into M spectrally sliced tributaries by a first programmable WS (WS1), see Fig. S2. The filtering as well as the further propagation of each signal tributary to the respective IQ receiver can be modelled by a time-domain optical impulse response $\underline{h}_{\text{S},\mu}(t)$, which can be represented by the associated complex-valued optical transfer function $\underline{\tilde{h}}_{\text{S},\mu}(f)$ in the frequency domain. For convenience, we define the corresponding baseband transfer function $\underline{\tilde{H}}_{\text{S},\mu}(f) = \underline{\tilde{h}}_{\text{S},\mu}(f + f_0)$ by down-shifting $\underline{\tilde{h}}_{\text{S},\mu}(f)$ by the optical carrier frequency f_0. The spectrum of the optical signal reaching the IQ receiver of each slice μ can thus be written as

$$\begin{aligned}\underline{\tilde{a}}_{\text{S},\mu}(f) &= \underline{\tilde{h}}_{\text{S},\mu}(f)\,\underline{\tilde{a}}_{\text{S}}(f) \\ &= \underline{\tilde{H}}_{\text{S},\mu}(f - f_0)\,\tilde{A}_{\text{S}}(f - f_0)\,e^{\text{j}\varphi_0} \\ &= \underline{\tilde{H}}_{\text{S},\mu}(f - f_0)\,\tilde{H}_{\text{EO}}(f - f_0)\,\tilde{U}_{\text{S}}(f - f_0)\,e^{\text{j}\varphi_0}\end{aligned} \tag{S5}$$

At the IQ receiver, the various spectrally sliced tributaries $\underline{\tilde{a}}_{\text{S},\mu}(f)$ are fed to an array of 90° optical hybrids (90° OH in Fig. S2), where they are superimposed with phase-locked tones of a Kerr soliton comb acting as a multi-wavelength LO. Note that the pump tone for the LO comb is derived from the same laser used as optical carrier at frequency f_0, onto which we modulate the input signal of our photonic-electronic ADC. Since the phase noise of the pump is essentially inherited to the tones of a Kerr comb, the phase noise of the various signal slices and of the corresponding LO tones is highly correlated and can thus be neglected, and we may model the LO comb by a simple superposition of harmonic signals. Each harmonic signal is defined by a corresponding distinct frequency f_ν ($\nu = -N \ldots N$), a real-valued amplitude $A_{\text{comb},\nu}$, and a phase of φ_ν. The phases φ_ν account for an optical phase offset of the comb as a whole, for slow phase drifts of the comb tones with respect to one another, as well as for a group delay that the comb may have experienced prior to interfering with the signal and that results in a constant phase increment between neighbouring comb lines,

$$\begin{aligned}\underline{a}_{\text{comb}}(t) &= \sum_\nu \underline{a}_{\text{comb},\nu}(t) = \sum_\nu A_{\text{comb},\nu}\,\text{e}^{\text{j}(2\pi f_\nu t + \varphi_\nu)} \\ \underline{\tilde{a}}_{\text{comb}}(f) &= \sum_\nu \underline{\tilde{a}}_{\text{comb},\nu}(f) = \sum_\nu A_{\text{comb},\nu}\,\delta(f - f_\nu)\,\text{e}^{\text{j}\varphi_\nu}\end{aligned} \tag{S6}$$

In this relation, $\delta(f)$ refers to the Dirac delta distribution. A second programmable waveshaper (WS2) is used to select M LO tones at frequencies $f_{\text{LO},\mu}$ ($\mu = 1 \ldots M$) from the comb. Note that in our scheme, the LO tones correspond to the first M comb lines with odd indices $f_{\text{LO},\mu} = f_{2\mu-1}$, see Fig. S1b. The spacing between adjacent LO tones is therefore twice the free spectral range f_{FSR} of the frequency comb, $f_{\text{LO},\mu+1} - f_{\text{LO},\mu} = 2 f_{\text{FSR}}$ $(\mu = 1, 2, \ldots, M-1)$. The amplitudes and the phases of the LO tones are denoted as $A_{\text{LO},\mu} = A_{\text{comb},2\mu-1}$ and $\varphi_{\text{LO},\mu} = \varphi_{2\mu-1}$ respectively. The band-pass filtering and the optical propagation of the μ-th LO tone is described by the optical transfer function $\underline{\tilde{h}}_{\text{LO},\mu}(f)$, and the corresponding baseband transfer function is denoted as $\underline{\tilde{H}}_{\text{LO},\mu}(f) = \underline{\tilde{h}}_{\text{LO},\mu}(f + f_0)$. Since each band-pass-type transfer function with index μ selects just a single LO tone at frequency $f_{\text{LO},\mu}$, the filtering leads to a collapse of the sum over the comb-tone indices $\nu = -N \ldots N$ in Eq. (S6) to only one term with index $2\mu - 1$. The optical spectrum of the LO tone at the input of IQ receiver μ can thus be written as

$$\begin{aligned}\tilde{\underline{a}}_{\mathrm{LO},\mu}(f) &= \tilde{\underline{h}}_{\mathrm{LO},\mu}(f)\,\tilde{\underline{a}}_{\mathrm{comb}}(f) \\ &= \tilde{\underline{H}}_{\mathrm{LO},\mu}(f-f_0)\sum_{\nu} A_{\mathrm{comb},\nu}\mathrm{e}^{\mathrm{j}\varphi_\nu}\delta(f-f_\nu) \\ &= \tilde{\underline{H}}_{\mathrm{LO},\mu}\left(f_{\mathrm{LO},\mu}-f_0\right)A_{\mathrm{LO},\mu}\mathrm{e}^{\mathrm{j}\varphi_{\mathrm{LO},\mu}}\delta\left(f-f_{\mathrm{LO},\mu}\right)\end{aligned} \qquad \text{(S7)}$$

At the 90° OH, the various signal tributaries $\underline{a}_{\mathrm{S},\mu}(t)$ are combined with their corresponding LO tones $\underline{a}_{\mathrm{LO},\mu}(t)$. For simplicity, we assume a set of ideal 90° OH, for which, disregarding the characteristic $\pi/2$ differences of the phase shifts along the various paths, the propagation of the signal slice μ and of the corresponding LO tone from the input of the 90° OH to the respective photodetector can be represented by a common time-domain impulse response $\underline{h}_{\mathrm{OH},\mu}(t)$. The resulting optical output signals, transmitted through optical fibres to the positive-polarity (+) and the negative-polarity (–) ports of the various BPD (Fig. S2), can be then expressed as

$$\begin{aligned}\underline{a}_{\mathrm{I}\pm,\mu}(t) &= \underline{h}_{\mathrm{OH},\mu}(t)*\left(\underline{a}_{\mathrm{S},\mu}(t)\pm\underline{a}_{\mathrm{LO},\mu}(t)\right) \\ \underline{a}_{\mathrm{Q}\pm,\mu}(t) &= \underline{h}_{\mathrm{OH},\mu}(t)*\left(\underline{a}_{\mathrm{S},\mu}(t)\pm\mathrm{j}\,\underline{a}_{\mathrm{LO},\mu}(t)\right)\end{aligned} \qquad \text{(S8)}$$

The phase shifts of $\pm\pi$ and $\pm\pi/2$ imposed by the 90° OH are represented by the factors of ± 1 and $\pm\mathrm{j}$ in Eq. (S8).

The dynamics of the various balanced photodetectors are described by baseband impulse responses $H_{\mathrm{BPD,I},\mu}(t)$ and $H_{\mathrm{BPD,Q},\mu}(t)$, such that the photocurrents representing the I and Q components can be written as

$$\begin{aligned} i_{\mathrm{I}\pm,\mu}(t) &= H_{\mathrm{BPD,I},\mu}(t)*\left|\underline{a}_{\mathrm{I}\pm,\mu}(t)\right|^2 \\ i_{\mathrm{Q}\pm,\mu}(t) &= H_{\mathrm{BPD,Q},\mu}(t)*\left|\underline{a}_{\mathrm{Q}\pm,\mu}(t)\right|^2\end{aligned} \qquad \text{(S9)}$$

where the symbol $*$ denotes a time-domain convolution. The difference currents $i_{\mathrm{I}+,\mu}(t)-i_{\mathrm{I}-,\mu}(t)$ and $i_{\mathrm{Q}+,\mu}(t)-i_{\mathrm{Q}-,\mu}(t)$ at the output of the BPD are fed to electronic ADC, which are modelled with equivalent baseband impulse responses $H_{\mathrm{ADC,I},\mu}(t)$ and $H_{\mathrm{ADC,Q},\mu}(t)$, and the ADC outputs deliver raw "digitized" voltages $U_{\mathrm{I},\mu}(t)$ and $U_{\mathrm{Q},\mu}(t)$ for the I and Q components. By inserting Eq. (S8) into Eq. (S9) and by accounting for the ADC impulse responses, the raw digitized voltages can be written as

$$\begin{aligned} U_{\mathrm{raw,I},\mu}(t) &= H_{\mathrm{ADC,I},\mu}(t)*H_{\mathrm{BPD,I},\mu}(t)*\left(i_{\mathrm{I}+,\mu}(t)-i_{\mathrm{I}-,\mu}(t)\right) \\ &= H_{\mathrm{ADC,I},\mu}(t)*H_{\mathrm{BPD,I},\mu}(t)*4\,\mathrm{Re}\left\{\left(\underline{h}_{\mathrm{OH},\mu}(t)*\underline{a}_{\mathrm{S},\mu}(t)\right)\left(\underline{h}^*_{\mathrm{OH},\mu}(t)*\underline{a}^*_{\mathrm{LO},\mu}(t)\right)\right\} \\ U_{\mathrm{raw,Q},\mu}(t) &= H_{\mathrm{ADC,Q},\mu}(t)*H_{\mathrm{BPD,Q},\mu}(t)*\left(i_{\mathrm{Q}+,\mu}(t)-i_{\mathrm{Q}-,\mu}(t)\right) \\ &= H_{\mathrm{ADC,Q},\mu}(t)*H_{\mathrm{BPD,Q},\mu}(t)*4\,\mathrm{Im}\left\{\left(\underline{h}_{\mathrm{OH},\mu}(t)*\underline{a}_{\mathrm{S},\mu}(t)\right)\left(\underline{h}^*_{\mathrm{OH},\mu}(t)*\underline{a}^*_{\mathrm{LO},\mu}(t)\right)\right\}\end{aligned} \qquad \text{(S10)}$$

where the superscripted symbol * refers to the complex conjugate. The quantities $U_{\mathrm{I},\mu}(t)$ and $U_{\mathrm{Q},\mu}(t)$ are interpreted as the real and the imaginary part of a complex-valued signal $\underline{U}_{\mu}(t)$, representing the raw digitized waveform of one spectral slice,

$$\underline{U}_{\mathrm{raw},\mu}(t) = U_{\mathrm{raw,I},\mu}(t)+\mathrm{j}\,U_{\mathrm{raw,Q},\mu}(t) \qquad \text{(S11)}$$

Note that the IQ receiver may have an imbalance between I and Q channels due to different characteristics of the BPD and ADC. In a next step, we merge all characteristics of the I channel of IQ receiver μ into a single impulse response $H_{\mathrm{Rx},\mu}(t)$, and we define an additional impulse response $H_{\mathrm{imb},\mu}(t)$ that accounts for the IQ-imbalance,

$$\begin{aligned} H_{\mathrm{Rx},\mu}(t) &= 4\times H_{\mathrm{ADC,I},\mu}(t)*H_{\mathrm{BPD,I},\mu}(t) \\ H_{\mathrm{Rx},\mu}(t)*H_{\mathrm{imb},\mu}(t) &= 4\times H_{\mathrm{ADC,Q},\mu}(t)*H_{\mathrm{BPD,Q},\mu}(t)\end{aligned} \qquad \text{(S12)}$$

The above equations (S10), (S11), and (S12) can then be combined into

$$
\begin{aligned}
\underline{U}_{\mathrm{raw},\mu}(t) = H_{\mathrm{Rx},\mu}(t) * \Big[& \mathrm{Re}\Big\{ \big(\underline{h}_{\mathrm{OH},\mu}(t) * \underline{a}_{\mathrm{S},\mu}(t) \big) \big(\underline{h}^{*}_{\mathrm{OH},\mu}(t) * \underline{a}^{*}_{\mathrm{LO},\mu}(t) \big) \Big\} \\
& + \mathrm{j} H_{\mathrm{imb},\mu}(t) * \mathrm{Im}\Big\{ \big(\underline{h}_{\mathrm{OH},\mu}(t) * \underline{a}_{\mathrm{S},\mu}(t) \big) \big(\underline{h}^{*}_{\mathrm{OH},\mu}(t) * \underline{a}^{*}_{\mathrm{LO},\mu}(t) \big) \Big\} \Big]
\end{aligned}
\tag{S13}
$$

For signal reconstruction, it is necessary to compensate for the IQ-imbalance through a pre-calibration step. This involves directly feeding a continuous wave (CW) laser to the IQ receivers and sweeping the frequency such that the beating frequency between the laser and the LO covers the entire electrical bandwidth of the IQ receivers. Further details on this technique can be found in Section S3 below. This pre-calibration procedure allows to extract the additional impulse response $H_{\mathrm{imb},\mu}(t)$ in the digital domain. Once $H_{\mathrm{imb},\mu}(t)$ is known, we can correct the imaginary part of $\underline{U}_{\mathrm{raw},\mu}(t)$. for the additional impulse response by a division by $\tilde{H}_{\mathrm{imb}.\mu}(f)$ in the frequency domain. This leads to the corrected complex-valued waveform $\underline{U}_{\mu}(t)$, which only depends on the single impulse response $H_{\mathrm{Rx},\mu}(t)$,

$$
\underline{U}_{\mu}(t) = H_{\mathrm{Rx},\mu}(t) * \Big[\big(\underline{h}_{\mathrm{OH},\mu}(t) * \underline{a}_{\mathrm{S},\mu}(t) \big) \big(\underline{h}^{*}_{\mathrm{OH},\mu}(t) * \underline{a}^{*}_{\mathrm{LO},\mu}(t) \big) \Big] \tag{S14}
$$

We obtain the representation of the spectral slice by a Fourier transformation of Eq. (S14) and by substitution of Eq. (S5) and (S7),

$$
\begin{aligned}
\underline{\tilde{U}}_{\mu}(f) &= \tilde{H}_{\mathrm{Rx},\mu}(f) \Big\{ \big(\underline{\tilde{h}}_{\mathrm{OH},\mu}(f)\, \underline{\tilde{a}}_{\mathrm{S},\mu}(f) \big) * \big(\underline{\tilde{h}}^{*}_{\mathrm{OH},\mu}(-f)\, \underline{\tilde{a}}^{*}_{\mathrm{LO},\mu}(-f) \big) \Big\} \\
&= \tilde{H}_{\mathrm{Rx},\mu}(f) \Big\{ \big(\underline{\tilde{H}}_{\mathrm{OH},\mu}(f-f_0)\, \underline{\tilde{H}}_{\mathrm{S},\mu}(f-f_0)\, \tilde{A}_{\mathrm{S}}(f-f_0)\, e^{\mathrm{j}\varphi_0} \big) \\
&\quad * \big(\underline{\tilde{H}}^{*}_{\mathrm{OH},\mu}(-f-f_0)\, \underline{\tilde{H}}^{*}_{\mathrm{LO},\mu}(f_{\mathrm{LO},\mu}-f_0)\, A_{\mathrm{LO},\mu} \mathrm{e}^{-\mathrm{j}\varphi_{\mathrm{LO},\mu}} \delta(f+f_{\mathrm{LO},\mu}) \big) \Big\} \\
&= \tilde{H}_{\mathrm{Rx},\mu}(f)\, \underline{\tilde{H}}^{*}_{\mathrm{LO},\mu}(f_{\mathrm{LO},\mu}-f_0)\, A_{\mathrm{LO},\mu} e^{-\mathrm{j}(\varphi_{\mathrm{LO},\mu}-\varphi_0)} \\
&\quad \times \Big\{ \big(\underline{\tilde{H}}_{\mathrm{OH},\mu}(f-f_0)\, \underline{\tilde{H}}_{\mathrm{S},\mu}(f-f_0)\, \tilde{A}_{\mathrm{S}}(f-f_0) \big) * \big(\underline{\tilde{H}}^{*}_{\mathrm{OH},\mu}(f_{\mathrm{LO},\mu}-f_0)\, \delta(f+f_{\mathrm{LO},\mu}) \big) \Big\} \\
&= \tilde{H}_{\mathrm{Rx},\mu}(f)\, \underline{\tilde{H}}^{*}_{\mathrm{LO},\mu}(f_{\mathrm{LO},\mu}-f_0)\, A_{\mathrm{LO},\mu} e^{-\mathrm{j}(\varphi_{\mathrm{LO},\mu}-\varphi_0)}\, \underline{\tilde{H}}^{*}_{\mathrm{OH},\mu}(f_{\mathrm{LO},\mu}-f_0) \\
&\quad \times \Big\{ \big(\underline{\tilde{H}}_{\mathrm{OH},\mu}(f-f_0)\, \underline{\tilde{H}}_{\mathrm{S},\mu}(f-f_0)\, \tilde{A}_{\mathrm{S}}(f-f_0) \big) * \delta(f+f_{\mathrm{LO},\mu}) \Big\} \\
&= \tilde{H}_{\mathrm{Rx},\mu}(f)\, \underline{\tilde{H}}^{*}_{\mathrm{OH},\mu}(f_{\mathrm{LO},\mu}-f_0)\, \underline{\tilde{H}}^{*}_{\mathrm{LO},\mu}(f_{\mathrm{LO},\mu}-f_0)\, A_{\mathrm{LO},\mu} e^{-\mathrm{j}(\varphi_{\mathrm{LO},\mu}-\varphi_0)} \\
&\quad \times \underline{\tilde{H}}_{\mathrm{OH},\mu}\big(f+(f_{\mathrm{LO},\mu}-f_0)\big)\, \underline{\tilde{H}}_{\mathrm{S},\mu}\big(f+(f_{\mathrm{LO},\mu}-f_0)\big)\, \tilde{A}_{\mathrm{S}}\big(f+(f_{\mathrm{LO},\mu}-f_0)\big)
\end{aligned}
\tag{S15}
$$

In a next step, we introduce intermediate frequencies (IF) for each slice μ , $f_{\mathrm{IF},\mu} = f_{\mathrm{LO},\mu} - f_0$ ($|f_{\mathrm{IF},\mu}| \ll f_0$, $\mu = 1,2,\ldots,M$) and combine all opto-electronic transfer functions into one LO-dependent baseband transfer function $\underline{\tilde{H}}_{\mathrm{OE},\mu}(f)$ for each slice μ, connecting the optical signal output of the MZM at Marker Ⓐ to the ADC outputs in Fig. S1,

$$
\underline{\tilde{H}}_{\mathrm{OE},\mu}(f) = \tilde{H}_{\mathrm{Rx},\mu}(f)\, \underline{\tilde{H}}^{*}_{\mathrm{OH},\mu}(f_{\mathrm{IF},\mu})\, \underline{\tilde{H}}^{*}_{\mathrm{LO},\mu}(f_{\mathrm{IF},\mu})\, \underline{\tilde{H}}_{\mathrm{OH},\mu}(f+f_{\mathrm{IF},\mu})\, \underline{\tilde{H}}_{\mathrm{S},\mu}(f+f_{\mathrm{IF},\mu})\, A_{\mathrm{LO},\mu} e^{-\mathrm{j}(\varphi_{\mathrm{LO},\mu}-\varphi_0)} \tag{S16}
$$

Equation (S15) can thus be re-written as

$$
\underline{\tilde{U}}_{\mu}(f) = \underline{\tilde{H}}_{\mathrm{OE},\mu}(f)\, \tilde{A}_{\mathrm{S}}(f+f_{\mathrm{IF},\mu}) \tag{S17}
$$

So far, the detected baseband signals $\underline{\tilde{U}}_{\mu}(f)$ are assumed to be noiseless. In reality, however, they are impaired by shot noise, thermal noise, and quantization noise of the ADC. The various noise contributions are statistically independent among each other, and their superposition can be modelled as additive white Gaussian noise (AWGN) with random amplitude spectrum $\underline{\tilde{N}}_{\mathrm{Rx},\mu}(f)$. Using the transfer functions defined in Eq. (S16) and including the random noise contributions, the complex-valued digitized waveforms $\underline{U}_{\mu}(t)$ can be written as

$$\underline{\tilde{U}}_\mu(f) = \underline{\tilde{H}}_{\mathrm{OE},\mu}(f)\tilde{A}_\mathrm{S}(f + f_{\mathrm{IF},\mu}) + \underline{\tilde{N}}_{\mathrm{Rx},\mu}(f) \tag{S18}$$

This relation serves as a base for signal reconstruction as described in the next section.

S2.2. Signal reconstruction and compensation of phase drifts

For reconstructing the original signal $U_\mathrm{S}(t)$, we first estimate on the sidebands of the optical signal $A_\mathrm{S}(f)$ by merging the various complex-valued baseband signals $\underline{\tilde{U}}_\mu(f)$ according to Eq. (S18) by digital signal processing. To this end, we first apply a numerical frequency shift to the baseband $\underline{\tilde{U}}_\mu(f)$ corresponding to the various slices μ, with the respective intermediate frequencies $f_{\mathrm{IF},\mu} = f_{\mathrm{LO},\mu} - f_0$,

$$\underline{\tilde{U}}_\mu(f - f_{\mathrm{IF},\mu}) = \underline{\tilde{H}}_{\mathrm{OE},\mu}(f - f_{\mathrm{IF},\mu})\tilde{A}_\mathrm{S}(f) + \underline{\tilde{N}}_{\mathrm{Rx},\mu}(f - f_{\mathrm{IF},\mu}) \tag{S19}$$

Considering all M slices, Eq. (S19) can be re-written by using column matrices,

$$\underbrace{\begin{pmatrix} \underline{\tilde{U}}_1(f - f_{\mathrm{IF},1}) \\ \underline{\tilde{U}}_2(f - f_{\mathrm{IF},2}) \\ \vdots \\ \underline{\tilde{U}}_M(f - f_{\mathrm{IF},M}) \end{pmatrix}}_{\underline{\tilde{\mathbf{U}}}(f)} = \underbrace{\begin{pmatrix} \underline{\tilde{H}}_{\mathrm{OE},1}(f - f_{\mathrm{IF},1}) \\ \underline{\tilde{H}}_{\mathrm{OE},2}(f - f_{\mathrm{IF},2}) \\ \vdots \\ \underline{\tilde{H}}_{\mathrm{OE},M}(f - f_{\mathrm{IF},M}) \end{pmatrix}}_{\underline{\tilde{\mathbf{H}}}_\mathrm{OE}(f)} \tilde{A}_\mathrm{S}(f) + \underbrace{\begin{pmatrix} \underline{\tilde{N}}_{\mathrm{Rx},1}(f - f_{\mathrm{IF},1}) \\ \underline{\tilde{N}}_{\mathrm{Rx},2}(f - f_{\mathrm{IF},2}) \\ \vdots \\ \underline{\tilde{N}}_{\mathrm{Rx},M}(f - f_{\mathrm{IF},M}) \end{pmatrix}}_{\underline{\tilde{\mathbf{N}}}_\mathrm{Rx}(f)} \tag{S20}$$

For simplicity, we write the column matrices with bold letters as indicated by the underbraces in Eq. (S20),

$$\underline{\tilde{\mathbf{U}}}(f) = \underline{\tilde{\mathbf{H}}}_\mathrm{OE}(f)\tilde{A}_\mathrm{S}(f) + \underline{\tilde{\mathbf{N}}}_\mathrm{Rx}(f) \tag{S21}$$

For signal reconstruction, the column matrix of frequency-dependent opto-electronic transfer functions $\underline{\tilde{\mathbf{H}}}_\mathrm{OE}(f)$ must be known, which requires an independent calibration measurement of the system, see Section S5 below. Importantly, the transfer functions $\underline{\tilde{\mathbf{H}}}_\mathrm{OE}(f)$ are impacted by amplitude and phase fluctuations of the comb lines as well as by slow phase drifts introduced by the fibres along the various signal paths. These variations occur on a long time scale τ and can be described by a slow τ-dependence of the expression $\underline{\tilde{H}}_{\mathrm{OE},\tau,\mu}(\tau) = A_{\mathrm{LO},\mu}(\tau)e^{-\mathrm{j}(\varphi_{\mathrm{LO},\mu}(\tau) - \varphi_0(\tau))}$ in Eq. (S16). Specifically, the optical linewidth of our comb tones amounts to only a few kHz, and mechanical vibrations in our setup are also limited to frequencies below a few kHz, such that these amplitude and phase drifts occur on time scales τ of hundreds of microseconds. The transfer functions $\underline{\tilde{\mathbf{H}}}_\mathrm{OE}(f)$ may thus be considered constant during one of our 8 µs-long recordings, but they may differ from recording to recording. This can be modelled by splitting the column matrix of transfer functions $\underline{\tilde{\mathbf{H}}}_\mathrm{OE}(f)$ into a time-invariant, but frequency-dependent part $\underline{\tilde{\mathbf{H}}}_{\mathrm{OE},f}(f)$, and in a frequency-independent time-variant part $\underline{\tilde{\mathbf{H}}}_{\mathrm{OE},\tau}$ that changes slowly on the longer time scale τ (> 100 µs),

$$\underline{\tilde{\mathbf{H}}}_\mathrm{OE}(\tau, f) = \underline{\tilde{\mathbf{H}}}_{\mathrm{OE},\tau}(\tau) \odot \underline{\tilde{\mathbf{H}}}_{\mathrm{OE},f}(f) \tag{S22}$$

where $\odot$ denotes the Hadamard product, i.e., the element-wise multiplication of the two column matrices $\underline{\tilde{\mathbf{H}}}_{\mathrm{OE},\tau}(\tau)$ and $\underline{\tilde{\mathbf{H}}}_{\mathrm{OE},f}(f)$. The time-invariant frequency-dependent part $\underline{\tilde{\mathbf{H}}}_{\mathrm{OE},f}(f)$ of the column matrix of transfer functions $\underline{\tilde{\mathbf{H}}}_\mathrm{OE}(f)$ accounts for the spectral characteristics of the components used in the opto-electronic paths as well as for the group delay and the dispersion introduced by the optical fibres and EDFA in our system. For measuring $\underline{\tilde{\mathbf{H}}}_{\mathrm{OE},f}(f)$, we perform a one-time calibration measurement in which we feed the system with a known reference waveform, derived from an ultra-stable femtosecond laser with well-defined pulse shape, small repetition rate and thus densely spaced comb lines, see Section S5 for details. By comparing the measured complex-valued signal spectra $\underline{\tilde{U}}_\mu(f - f_{\mathrm{IF},\mu})$ of the various slices with the known signal spectrum at the input, we can retrieve the time-invariant matrix of transfer functions $\underline{\tilde{\mathbf{H}}}_{\mathrm{OE},f}(f)$.

In a next step, the slowly time-variant complex-valued factors $\underline{\tilde{\mathbf{H}}}_{\mathrm{OE},\tau}(\tau)$ of the opto-electronic transfer functions $\underline{\tilde{\mathbf{H}}}_\mathrm{OE}(f)$ need to be estimated for each recording. To this end, we isolate a complex-valued factor

$\underline{C}(\tau) = A_{\mathrm{LO},1}(\tau) e^{-\mathrm{j}(\varphi_{\mathrm{LO},1}(\tau)-\varphi_0(\tau))}$ associated with the first spectral slice, which we use as a reference,

$$\tilde{\underline{\mathbf{H}}}_{\mathrm{OE},\tau}(\tau) = \underline{C}(\tau) \cdot \tilde{\underline{\mathbf{H}}}'_{\mathrm{OE},\tau}(\tau) \tag{S23}$$

This ensures the first element of the remaining column matrix $\tilde{\underline{\mathbf{H}}}'_{\mathrm{OE},\tau}(\tau)$ is equal to 1, $\tilde{\underline{H}}'_{\mathrm{OE},\tau,1} = 1$, while the other matrix elements $\tilde{\underline{H}}'_{\mathrm{OE},\tau,\mu}$ ($\mu = 2,3,...,M$) represent the amplitude and phase differences between the μ-th slice and the first slice,

$$\tilde{\underline{\mathbf{H}}}_{\mathrm{OE},\tau}(\tau) = \underline{C}(\tau) \cdot \underbrace{\begin{pmatrix} 1 \\ \dfrac{A_{\mathrm{LO},2}(\tau)}{A_{\mathrm{LO},1}(\tau)} e^{-\mathrm{j}(\varphi_{\mathrm{LO},2}(\tau)-\varphi_{\mathrm{LO},1}(\tau))} \\ \vdots \\ \dfrac{A_{\mathrm{LO},M}(\tau)}{A_{\mathrm{LO},1}(\tau)} e^{-\mathrm{j}(\varphi_{\mathrm{LO},M}(\tau)-\varphi_{\mathrm{LO},1}(\tau))} \end{pmatrix}}_{\tilde{\underline{\mathbf{H}}}'_{\mathrm{OE},\tau}(\tau)} \tag{S24}$$

To obtain the remaining components $\tilde{\underline{H}}'_{\mathrm{OE},\tau,\mu}(\tau) = A_{\mathrm{LO},\mu}(\tau) \big/ A_{\mathrm{LO},1}(\tau) \cdot e^{-\mathrm{j}(\varphi_{\mathrm{LO},\mu}(\tau)-\varphi_{\mathrm{LO},1}(\tau))}$ ($\mu = 2,3,...,M$) of $\tilde{\underline{\mathbf{H}}}_{\mathrm{OE},\tau}$, we first correct each of the measured spectral slices by the corresponding time-invariant part $\tilde{\underline{\mathbf{H}}}_{\mathrm{OE},f}(f)$ of the measured transfer function and then compare the resulting complex-valued spectra in the spectral overlap regions between adjacent slices. This leads to a recursive relation of the form:

$$\tilde{\underline{H}}'_{\mathrm{OE},\tau,\mu}(\tau) = \tilde{\underline{H}}'_{\mathrm{OE},\tau,\mu-1}(\tau) \cdot \left\langle \frac{\tilde{\underline{H}}^{-1}_{\mathrm{OE},f,\mu}(f - f_{\mathrm{IF},\mu}) \tilde{\underline{U}}_{\mu}(f - f_{\mathrm{IF},\mu})}{\tilde{\underline{H}}^{-1}_{\mathrm{OE},f,\mu-1}(f - f_{\mathrm{IF},\mu-1}) \tilde{\underline{U}}_{\mu-1}(f - f_{\mathrm{IF},\mu-1})} \right\rangle_{\mathrm{OR}}, \mu = 2,3,...,M \tag{S25}$$

where $\tilde{\underline{H}}'_{\mathrm{OE},\tau,1} = 1$. In this relation, $\tilde{\underline{H}}^{-1}_{\mathrm{OE},f,\mu}(f - f_{\mathrm{IF},\mu})$ corresponds to the inverse of the previously measured time-invariant transfer function for slice μ, and $\langle ... \rangle_{\mathrm{OR}}$ denotes an average over the spectral overlap region (OR) within which both tributary signals $\tilde{\underline{U}}_{\mu}(f - f_{\mathrm{IF},\mu})$ and $\tilde{\underline{U}}_{\mu-1}(f - f_{\mathrm{IF},\mu-1})$ are sufficiently strong. Optical stitching tones can be artificially added to the OR if the signals under consideration obtain nor or too little spectral content within the OR.

Using the procedures described in the previous paragraphs, we obtain a column matrix of transfer functions $\tilde{\underline{\mathbf{H}}}'_{\mathrm{OE}}(f) = \tilde{\underline{\mathbf{H}}}'_{\mathrm{OE},\tau}(\tau) \odot \tilde{\underline{\mathbf{H}}}_{\mathrm{OE},f}(f)$, which still differs from the complete transfer functions $\tilde{\underline{\mathbf{H}}}_{\mathrm{OE},\tau}(\tau)$ by an unknown complex-valued factor $\underline{C}(\tau)$ according to Eq. (S22) to Eq. (S24),

$$\begin{aligned} \tilde{\underline{\mathbf{H}}}_{\mathrm{OE}}(f) &= \underline{C}(\tau) \cdot \tilde{\underline{\mathbf{H}}}'_{\mathrm{OE},\tau} \odot \tilde{\underline{\mathbf{H}}}_{\mathrm{OE},f}(f) \\ &= \underline{C}(\tau) \cdot \tilde{\underline{\mathbf{H}}}'_{\mathrm{OE}}(f) \end{aligned} \tag{S26}$$

Note that we have skipped the slow-time argument τ of $\tilde{\underline{\mathbf{H}}}'_{\mathrm{OE},\tau}$ and $\tilde{\underline{\mathbf{H}}}'_{\mathrm{OE}}(f)$ in Eq. (S26) and in the subsequent relations, since all relations refer to a specific recording for which $\tilde{\underline{\mathbf{H}}}'_{\mathrm{OE},\tau}$ and $\tilde{\underline{\mathbf{H}}}'_{\mathrm{OE}}(f)$ have already been estimated using the recursive relation in Eq. (S25). The only remaining τ-dependent unknown is hence the complex-valued factor $\underline{C}(\tau) = A_{\mathrm{LO},1}(\tau) e^{-\mathrm{j}(\varphi_{\mathrm{LO},1}(\tau)-\varphi_0(\tau))}$ in Eq. (S26), which accounts for the slow amplitude and phase drift of the first LO comb line as well as for the phase drift in the fibres along the signal and the LO path of the first slice. As explained above, these drift effects occur on time scales much larger than the observation time of the signal such that $\underline{C}(\tau)$ can be considered constant during one recording. To find a way for estimating $\underline{C}(\tau)$ from our recordings, we first insert Eq. (S26) into Eq. (S21), which leads to

$$\tilde{\underline{\mathbf{U}}}(f) = \tilde{\underline{\mathbf{H}}}'_{\mathrm{OE}}(f) \left(\underline{C}(\tau) \cdot \tilde{A}_{\mathrm{S}}(f) \right) + \tilde{\underline{\mathbf{N}}}_{\mathrm{Rx}}(f) \tag{S27}$$

We then first extract an intermediate estimate of

$$\tilde{\underline{A}}'_{\mathrm{S}}(f) = \underline{C}(\tau) \cdot \tilde{A}_{\mathrm{S}}(f) \tag{S28}$$

which still contains the unknown complex-valued factor $\underline{C}(\tau)$. Note that the spectrum $\tilde{A}_\mathrm{S}(f)$ of the time-domain envelope $A_\mathrm{S}(t)$ that is modulated on the optical carrier does not carry an underscore since $A_\mathrm{S}(t)$ is real-valued, see Eq. (S3). In contrast to that, the intermediate estimate $\underline{\tilde{A}}'_\mathrm{S}(f)$ corresponds to a complex-valued time-domain function $\underline{A}'_\mathrm{S}(t)$, and both quantities are hence marked with an underscore. Equation (S27) can then be re-written as

$$\underline{\tilde{\mathbf{U}}}(f) = \underline{\tilde{\mathbf{H}}}'_\mathrm{OE}(f)\,\underline{\tilde{A}}'_\mathrm{S}(f) + \underline{\tilde{\mathbf{N}}}_\mathrm{Rx}(f) \tag{S29}$$

From this relation, we can estimate $\underline{\tilde{A}}'_\mathrm{S}(f)$ using the maximum-ratio combing (MRC) algorithm [12] by multiplying the measured and numerically frequency-shifted complex-valued baseband signals $\underline{\tilde{\mathbf{U}}}(f)$ with the pseudo-inverse $\left(\underline{\tilde{\mathbf{H}}}'^{\dagger}_\mathrm{OE}(f)\,\underline{\tilde{\mathbf{H}}}'_\mathrm{OE}(f)\right)^{-1}\underline{\tilde{\mathbf{H}}}'^{\dagger}_\mathrm{OE}(f)$ of the column matrix $\underline{\tilde{\mathbf{H}}}'_\mathrm{OE}(f)$, that can be interpreted as the transfer matrix of a single-input multiple-output (SIMO) receiver,

$$\underline{\tilde{A}}'_\mathrm{S,est}(f) = \underbrace{\left(\underline{\tilde{\mathbf{H}}}'^{\dagger}_\mathrm{OE}(f)\,\underline{\tilde{\mathbf{H}}}'_\mathrm{OE}(f)\right)^{-1}\underline{\tilde{\mathbf{H}}}'^{\dagger}_\mathrm{OE}(f)}_{\text{pseudo-inverse of } \underline{\tilde{\mathbf{H}}}'_\mathrm{OE}(f)}\,\underline{\tilde{\mathbf{U}}}(f) \tag{S30}$$

Note that the pseudo-inverse of $\underline{\tilde{\mathbf{H}}}'_\mathrm{OE}(f)$ on the right-hand side of Eq. (30) can only be calculated for frequencies f, for which at least one element of the column matrix of opto-electronic transfer functions $\underline{\tilde{\mathbf{H}}}'_\mathrm{OE}(f)$ is non-zero. This is the case for frequencies $0 \le f \le f_{2M}$ and for some small frequency ranges to the left and the right of this interval, which are still within the roll-off-region of the transfer function $\underline{\tilde{H}}_\mathrm{OE,1}(f - f_\mathrm{IF,1})$ and $\underline{\tilde{H}}_{\mathrm{OE},M}(f - f_{\mathrm{IF},M})$. As a consequence, the intermediate estimate $\underline{\tilde{A}}'_\mathrm{S,est}(f)$ according to Eq. (S30) predominantly contains spectral components at non-negative frequencies $0 \le f \le f_\mathrm{ue}$ and some residual spectral components at slightly negative frequencies $f_\mathrm{le} - f_0 \le f < 0$, where f_ue and f_le correspond to optical frequencies associated with the upper and lower edge of the acquisition band. This can be seen in the right-hand sketch of the spectra at Point Ⓑ in Fig. S1, taking into account that the frequency axis in this plot refers to the optical frequency whereas Eq. (S30) is formulated for baseband frequencies f. The roll-off region just below the carrier f_0 in Fig. S1 hence corresponds to a small range of slightly negative frequencies $f_\mathrm{le} - f_0 \le f < 0$ in Eq. (S30). The residual spectral components at these slightly negative frequencies $f_\mathrm{le} - f_0 < f < 0$ in Eq. (S30) can be used to determine the phase $\varphi_\mathrm{C}(\tau) = \arg\{\underline{C}(\tau)\}$ of the complex-valued factor $\underline{C}(\tau)$. Note that it is generally sufficient to estimate the phase of the recording-dependent complex-valued factor $\underline{C}(\tau)$, since the magnitude $|\underline{C}(\tau)|$ simply corresponds to an overall scaling factor of the time-domain signal. To estimate the phase, we make use of the fact that the slowly varying envelope $A_\mathrm{S}(t)$ of the optical signal is real-valued and the corresponding spectrum is thus conjugate symmetric, i.e., $\tilde{A}_\mathrm{S}(f) = \tilde{A}^*_\mathrm{S}(-f)$. We may hence request that the same relation applies to the estimated reconstructed signal $\tilde{A}_\mathrm{S,est}(f)$

$$\tilde{A}_\mathrm{S,est}(f) = \tilde{A}^*_\mathrm{S,est}(-f) \tag{S31}$$

For frequencies f within the acquisition band, $f \in [f_\mathrm{le} - f_0, f_\mathrm{ue} - f_0]$, the estimated reconstructed signal $\tilde{A}_\mathrm{S,est}(f)$ is related to the intermediate estimate $\underline{\tilde{A}}'_\mathrm{S,est}(f)$ via the equivalent of Eq. (S28),

$$\underline{\tilde{A}}'_\mathrm{S,est}(f) = \underline{C}(\tau)\cdot\tilde{A}_\mathrm{S,est}(f) \ \text{ for } f \in [f_\mathrm{le} - f_0, f_\mathrm{ue} - f_0] \tag{S32}$$

Inserting Eq. (S32) into Eq. (S31) leads to

$$\underline{C}^{-1}(\tau)\,\underline{\tilde{A}}'_\mathrm{S,est}(f) = \underline{C}^{*-1}(\tau)\,\underline{\tilde{A}}'^{*}_\mathrm{S,est}(-f) \ \text{ for } f \in [f_\mathrm{le} - f_0, f_0 - f_\mathrm{le}] \tag{S33}$$

From this relation, the phase $\varphi_\mathrm{C}(\tau)$ of the complex-valued factor $\underline{C}(\tau) = |\underline{C}(\tau)|e^{\mathrm{j}\varphi_\mathrm{C}(\tau)}$ can be estimated by comparing the phases of spectral components at slightly negative and positive frequencies, i.e, within the low-frequency (LF) range $f_\mathrm{le} - f_0 \le f \le f_0 - f_\mathrm{le}$,

$$\varphi_\mathrm{C}(\tau) = \frac{1}{2}\arg\left\{\frac{\underline{C}(\tau)}{\underline{C}^*(\tau)}\right\} = \frac{1}{2}\arg\left\{\frac{\underline{\tilde{A}}'_\mathrm{S,est}(f)}{\underline{\tilde{A}}'^{*}_\mathrm{S,est}(-f)}\right\} = \frac{1}{2}\arg\left\{e^{\mathrm{j}2\varphi_\mathrm{C}(\tau)}\right\} \tag{S34}$$

To reduce the uncertainty, we may not only evaluate one pair of frequencies f and $-f$ in Eq. (S34), but average over the whole LF range $f_{\text{le}} - f_0 \le f \le f_0 - f_{\text{le}}$,

$$\varphi_{\text{C}}(\tau) = \frac{1}{2}\left\langle \arg\left\{ \frac{\tilde{\underline{A}}'_{\text{S,est}}(f)}{\tilde{\underline{A}}'^{*}_{\text{S,est}}(-f)} \right\} \right\rangle_{f_{\text{le}} - f_0 \le f \le f_0 - f_{\text{le}}} \tag{S35}$$

Note, however, that it was not possible to apply this technique for evaluating the measurements presented in the main manuscript because all spectral components of the use signal $\tilde{A}_{\text{S}}(f)$ in the LF range were effectively suppressed by the FBG that was used to eliminate the residual carrier, see Fig. S1. Additionally, the LF region does not always contain a signal component arising from modulation. We therefore rely on an alternative approach, which exploits the fact that our optical signal still contains a residual optical carrier that leaks through the MZM because of the limited extinction ratio of around 23 dB and that is still visible after the notch filter. This residual carrier corresponds to a DC offset of the slowly varying envelope, resulting in a Dirac-type peak $\underline{A}'_{\text{DC}} \cdot \delta(f) = \underline{C}(\tau) \cdot A_{\text{DC}} \cdot \delta(f)$ at zero frequency after reconstruction in the frequency domain, which is also visible in the reconstructed spectrum $\tilde{\underline{A}}'_{\text{S,est}}(f)$ and which features a real-valued weight $A_{\text{DC}} = \underline{C}^{-1}(\tau)\underline{A}'_{\text{DC}}$. The phase of the complex factor can hence be estimated from the complex-valued weight $\underline{A}'_{\text{DC}}$ of the corresponding delta peak found in the intermediate estimate, accessible by integrating the intermediate estimate $\tilde{\underline{A}}'_{\text{S,est}}(f) \approx \underline{A}'_{\text{DC}} \cdot \delta(f)$ over a small frequency interval $f \in [-f_\varepsilon, f_\varepsilon]$ with $f_\varepsilon \approx 100\,\text{kHz}$ around zero frequency

$$\begin{aligned} \varphi_{\text{C}}(\tau) &= \arg\left\{\underline{A}'_{\text{DC}}\right\} \\ &= \arg\left\{ \int_{-f_\varepsilon}^{f_\varepsilon} \tilde{\underline{A}}'_{\text{S,est}}(f)\, df \right\} \end{aligned} \tag{S36}$$

Based on the estimated phase factor $\varphi_{\text{C}}(\tau)$, we hence obtain the complex-valued factor $\underline{C}(\tau) = |\underline{C}(\tau)| e^{\text{j}\varphi_{\text{C}}(\tau)}$ to finally estimate $\tilde{A}_{\text{S,est}}(f)$ using Eq. (S32) and exploiting the Hermitian symmetry,

$$\tilde{A}_{\text{S,est}}(f) = \begin{cases} \underline{C}^{-1}(\tau)\tilde{\underline{A}}'_{\text{S,est}}(f) & \text{for } f \in [0, f_{\text{ue}} - f_0] \\ \left(\underline{C}^{-1}(\tau)\tilde{\underline{A}}'_{\text{S,est}}(-f)\right)^* & \text{for } f \in [-(f_{\text{ue}} - f_0), 0] \end{cases} \tag{S37}$$

According to Eqs. (S4) and (S5), we finally arrive at an estimate $U_{\text{S,est}}(t)$ of the original broadband RF signal $U_{\text{S}}(t)$ by first compensating for the EO transfer function of the MZM, and then the inverse Fourier transform

$$U_{\text{S,est}}(t) = \mathcal{F}^{-1}\left(\frac{\tilde{A}_{\text{S,est}}(f)}{\tilde{H}_{\text{EO}}(f)} \right) \tag{S38}$$

Note that the estimate $U_{\text{S,est}}(t)$ still differs from the original broadband THz waveform $U_{\text{S}}(t)$ by a constant multiplier that is related to the unknown magnitude $|\underline{C}(\tau)|$ of the complex-valued factor $\underline{C}(\tau)$. The scaling factor can finally be determined via a separate power calibration. To this end, we feed the system with an RF test signal of known power and compare the amplitude of the reconstructed digital signal to that of the known test signal. We then account for this scaling factor by applying a digital gain as the last step of our signal reconstruction. A more detailed description on the investigation of the frequency response of our ADC system is given in Section S6.

It should also be noted that our measurements and the associated reconstruction according to Eq. (S35) only relies on the magnitude $|\tilde{H}_{\text{EO}}(f)|$ of the transfer function of the MZM $\tilde{H}_{\text{EO}}(f)$, which was obtained through a separate calibration measurement and normalized to the response at 2 GHz, as detailed in Section S4. In the future, the phase of the transfer function might also be measured and considered in the reconstruction, e.g., by using a broadband RF excitation pulse with well-known time-domain shape and by applying the same strategy that we used for calibrating the photonic-electronic signal-processing engine, see Section S5. Coupling such a test pulse to the modulator could,

e.g., become possible by using ultra-broadband THz coupling elements that exploit 3D-printed metal-coated freeform structures based on multi-photon lithography [13].

S3. Calibration of the in-phase/quadrature (IQ) receiver

The photonic-electronic signal-processing engine of our ADC, see Fig. S1, relies on an array of optical IQ receivers, that are unavoidably subject to imbalances arising from differences in the group delays of the fibers leading to the balanced photodetectors and from non-identical transfer-functions of the associated photodiodes and acquisition electronics. To model this imbalance, we consider the impulse response of the I channel as a common receiver response $H_{\mathrm{Rx},\mu}(t)$ in the time domain, and we treat the response of the Q channel as a convolution of the common receiver response $H_{\mathrm{Rx},\mu}(t)$ with an additional IQ imbalance response $H_{\mathrm{imb},\mu}(t)$, see Section S2.1 above. This IQ imbalance response $H_{\mathrm{imb},\mu}(t)$ needs to be extracted from a calibration measurement and is then used to translate the "raw" digitized waveforms $\underline{U}_{\mathrm{raw},\mu}(t)$, see Eq. (S12), into their corrected counterparts $\underline{U}_{\mu}(t)$, see Eq. (S15).

To extract the IQ imbalance of the IQ receiver associated with a certain spectral slice (index μ), we it feed a continuous-wave test signal $\underline{a}_{\mathrm{test},\mu}(t) = A_{\mathrm{test},\mu}(t)\, e^{\mathrm{j}\left(2\pi f_{\mathrm{test},\mu} t + \varphi_{\mathrm{test},\mu}(t)\right)}$ derived from an external-cavity laser (ECL) and with the associated LO tone $\underline{a}_{\mathrm{LO},\mu}(t) = A_{\mathrm{LO},\mu}(t)\, e^{\mathrm{j}\left(2\pi f_{\mathrm{LO},\mu} t + \varphi_{\mathrm{LO},\mu}(t)\right)}$, and we record the resulting electric beat signal at the difference frequency $f_{\mathrm{b}} = f_{\mathrm{test},\mu} - f_{\mathrm{LO},\mu}$. In these relations, $A_{\mathrm{test},\mu}(t)$ and $A_{\mathrm{LO},\mu}(t)$ are real-valued amplitudes, and the associated phases are accounted for by $\varphi_{\mathrm{test},\mu}(t)$ and $\varphi_{\mathrm{LO},\mu}(t)$. For each IQ receiver μ, we sweep the emission frequency $f_{\mathrm{test},\mu}$ of the ECL in steps of 1 GHz, thereby ensuring that the beat frequency f_{b} of the laser tone and the corresponding LO tone covers the entire electrical bandwidth of the receiver. Ignoring the phase and the amplitude noise of the laser and the LO assuming constant amplitudes $A_{\mathrm{test},\mu}$ and $A_{\mathrm{LO},\mu}$ as well as constant phases $\varphi_{\mathrm{test},\mu}$, and $\varphi_{\mathrm{LO},\mu}$ for the duration of the measurement, the photocurrents obtained at the I and Q port of the IQ receiver can be written according to Eq. (S14),

$$
\begin{aligned}
U_{\mathrm{test,I},\mu}(t) &= A_{\mathrm{test},\mu} A_{\mathrm{LO},\mu} H_{\mathrm{Rx},\mu}(t) * \cos\left(2\pi f_{\mathrm{b}} t + \varphi_{\mathrm{b}}\right) \\
U_{\mathrm{test,Q},\mu}(t) &= A_{\mathrm{test},\mu} A_{\mathrm{LO},\mu} H_{\mathrm{Rx},\mu}(t) * H_{\mathrm{imb},\mu}(t) * \sin\left(2\pi f_{\mathrm{b}} t + \varphi_{\mathrm{b}}\right)
\end{aligned}
\tag{S39}
$$

where the frequency f_{b} and the phase φ_{b} of the beat note are given by

$$
\begin{aligned}
f_{\mathrm{b}} &= f_{\mathrm{test},\mu} - f_{\mathrm{LO},\mu} \\
\varphi_{\mathrm{b}} &= \varphi_{\mathrm{test},\mu} - \varphi_{\mathrm{LO},\mu}
\end{aligned}
\tag{S40}
$$

The I and Q signals of the photocurrent can be re-written in the frequency domain,

$$
\begin{aligned}
\tilde{U}_{\mathrm{test,I},\mu}(f) &= A_{\mathrm{test},\mu} A_{\mathrm{LO},\mu} \tilde{H}_{\mathrm{Rx},\mu}(f) \cdot \frac{1}{2}\left[e^{\mathrm{j}\varphi_{\mathrm{b}}} \delta(f - f_{\mathrm{b}}) + e^{-\mathrm{j}\varphi_{\mathrm{b}}} \delta(f + f_{\mathrm{b}})\right] \\
&= A_{\mathrm{test},\mu} A_{\mathrm{LO},\mu} \cdot \frac{1}{2}\left[e^{\mathrm{j}\varphi_{\mathrm{b}}} \tilde{H}_{\mathrm{Rx},\mu}(f_{\mathrm{b}}) + e^{-\mathrm{j}\varphi_{\mathrm{b}}} \tilde{H}_{\mathrm{Rx},\mu}(-f_{\mathrm{b}})\right] \\
\tilde{U}_{\mathrm{test,Q},\mu}(f) &= A_{\mathrm{test},\mu} A_{\mathrm{LO},\mu} \tilde{H}_{\mathrm{Rx},\mu}(f) \tilde{H}_{\mathrm{imb},\mu}(f) \cdot \frac{1}{2\mathrm{j}}\left[e^{\mathrm{j}\varphi_{\mathrm{b}}} \delta(f - f_{\mathrm{b}}) - e^{-\mathrm{j}\varphi_{\mathrm{b}}} \delta(f + f_{\mathrm{b}})\right] \\
&= A_{\mathrm{test},\mu} A_{\mathrm{LO},\mu} \cdot \frac{1}{2\mathrm{j}}\left[e^{\mathrm{j}\varphi_{\mathrm{b}}} \tilde{H}_{\mathrm{Rx},\mu}(f_{\mathrm{b}}) \tilde{H}_{\mathrm{imb},\mu}(f_{\mathrm{b}}) - e^{-\mathrm{j}\varphi_{\mathrm{b}}} \tilde{H}_{\mathrm{Rx},\mu}(-f_{\mathrm{b}}) \tilde{H}_{\mathrm{imb},\mu}(-f_{\mathrm{b}})\right]
\end{aligned}
\tag{S41}
$$

We then decompose the common transfer function $\tilde{H}_{\mathrm{Rx},\mu}(f_{\mathrm{b}})$ and the transfer function $\tilde{H}_{\mathrm{imb},\mu}(f_{\mathrm{b}})$ of the imbalance into their respective amplitude and phase and make use of the fact that both of them are Fourier transforms of a real-valued time-domain impulse response and thus feature Hermitian symmetry,

$$
\begin{aligned}
\tilde{H}_{\mathrm{Rx},\mu}(\pm f_{\mathrm{b}}) &= \left|\tilde{H}_{\mathrm{Rx},\mu}(f_{\mathrm{b}})\right| e^{\pm \mathrm{j}\varphi_{\mathrm{Rx}}(f_{\mathrm{b}})} \\
\tilde{H}_{\mathrm{imb},\mu}(\pm f_{\mathrm{b}}) &= \left|\tilde{H}_{\mathrm{imb},\mu}(f_{\mathrm{b}})\right| e^{\pm \mathrm{j}\varphi_{\mathrm{imb}}(f_{\mathrm{b}})}
\end{aligned}
\tag{S42}
$$

Inserting Eq. (S41) into (S40) and taking the inverse Fourier transform, we obtain

$$\begin{aligned}
U_{\text{test,I},\mu}(t) &= A_{\text{test},\mu} A_{\text{LO},\mu} \left|\tilde{H}_{\text{Rx},\mu}(f_\text{b})\right| \frac{1}{2}\left[e^{\text{j}(2\pi f_\text{b} t + \varphi_\text{b} + \varphi_\text{Rx}(f_\text{b}))} + e^{-\text{j}(2\pi f_\text{b} t + \varphi_\text{b} + \varphi_\text{Rx}(f_\text{b}))}\right] \\
&= A_{\text{test},\mu} A_{\text{LO},\mu} \left|\tilde{H}_{\text{Rx},\mu}(f_\text{b})\right| \cos\left(2\pi f_\text{b} t + \varphi_\text{b} + \varphi_\text{Rx}(f_\text{b})\right) \\
U_{\text{test,Q},\mu}(t) &= A_{\text{test},\mu} A_{\text{LO},\mu} \left|\tilde{H}_{\text{Rx},\mu}(f_\text{b})\right| \left|\tilde{H}_{\text{imb},\mu}(f_\text{b})\right| \frac{1}{2\text{j}}\left[e^{\text{j}(2\pi f_\text{b} t + \varphi_\text{b} + \varphi_\text{Rx}(f_\text{b}) + \varphi_\text{imb}(f_\text{b}))} - e^{-\text{j}(2\pi f_\text{b} t + \varphi_\text{b} + \varphi_\text{Rx}(f_\text{b}) + \varphi_\text{imb}(f_\text{b}))}\right] \\
&= A_{\text{test},\mu} A_{\text{LO},\mu} \left|\tilde{H}_{\text{Rx},\mu}(f_\text{b})\right| \left|\tilde{H}_{\text{imb},\mu}(f_\text{b})\right| \sin\left(2\pi f_\text{b} t + \varphi_\text{b} + \varphi_\text{Rx}(f_\text{b}) + \varphi_\text{imb}(f_\text{b})\right)
\end{aligned} \tag{S43}$$

For each beat frequency f_b, the amplitude $\left|\tilde{H}_{\text{imb},\mu}(f_\text{b})\right|$ and the associated phase $\varphi_\text{imb}(f_\text{b})$ can be extracted from the acquired raw digitized signals by simultaneously fitting cos- and sin-functions to the measured I and Q components $U_{\text{test,I},\mu}(t)$ and $U_{\text{test,Q},\mu}(t)$ in the time domain. By sweeping the frequency $f_{\text{test},\mu}$ of the ECL tone, we sample the imbalance transfer function $\tilde{H}_{\text{imb},\mu}(f_\text{b})$ at discrete points $f_\text{b} = f_{\text{test},\mu} - f_{\text{LO},\mu}$ and approximate the full transfer function by interpolation. For each IQ receiver, the resulting transfer function $\tilde{H}_{\text{imb}.\mu}(f)$ is then corrected for in the digital domain, leading to the corrected complex-valued time-domain waveforms $\underline{U}_\mu(t)$ according to Eq. (S15). Note that the linear slope of the phase φ_imb over frequency f indicates the skew, i.e., the group-delay difference between the I and Q paths, measured from the 90° OH to the ADC.

S4. Calibration of the Mach-Zehnder modulator (MZM) electro-optic (EO) response

The EO response of the thin-film lithium niobate MZM is obtained by applying pure sinusoidal test signals within a frequency range of 2 GHz to 320 GHz to the transmission-line electrodes of the MZM. The measurement method used is similar to the one described in Section 2c. of our pervious publication [3] and is as outlined in the Supplementary Information (SI) thereof. The test signals are coupled to the MZM by standard ground-signal-ground (GSG) probes with a 100 µm pitch. To cover the broad frequency range of interest, three different probes are employed, operating at DC – 110 GHz, 110 – 170 GHz and 220 – 320 GHz, respectively. Similarly, we rely on different signal sources for generating the various test signals: For the frequency range of 2 – 67 GHz, we use a standard RF signal generator (PSG, E8257D, Keysight Technologies Inc., CA, USA), while the frequency ranges of 70 – 110 GHz, 110 – 170 GHz, and 220 – 320 GHz are covered by combining the same RF signal generator with three different frequency multipliers (Virginia Diodes Inc.) operating at the corresponding frequency ranges. Attenuators or bandpass filters are used to ensure the appropriate signal level and purity of the generated signals. For RF frequencies below 67 GHz, the power at the input of the probe is measured using a high-speed oscilloscope (UXR 1004A, Keysight Technologies Inc., CA, USA), while RF power measurements above 67 GHz, are done with a calibrated waveguide-coupled colorimeter (Erickson PM4, Virginia Diodes Inc.). Based on the known power transmission curve $\left|S_{21}(f)\right|^2$ of the GSG probes, we can thus calculate the power $P_\text{RF}(f)$ arriving at the MZM electrodes as a function of frequency. This information is depicted in Fig. S3a, where the red markers represent the estimated powers that propagate along the on-chip RF waveguides. For each applied test signal of frequency *f*, we record the optical spectra at the MZM output using an optical spectrum analyser (OSA, ANDO). From the peak value of the respective optical sideband of interest we extract the optical power $P_\text{opt}(f_0 + f)$, where f_0 denotes the frequency of the optical carrier fed to the MZM. These values are represented by the blue markers shown in Fig. S3a.

To obtain the EO response of the MZM, we finally divide the optical sideband power by the electrical power applied to the electrodes,

$$P_\text{opt}(f_0 + f)/P_\text{RF}(f) = \left|\tilde{H}_\text{EO}(f)\right|^2 \tag{S44}$$

We normalize the resulting transfer function to its value in the low-frequency limit, i.e., at 2 GHz. The resulting EO transfer function is plotted in Fig. S3b using blue circles as markers of the various data points. The strong dip observed at around 290 GHz is a result of a periodically structured array of buried-metal back-reflectors, which were designed to improve the optical coupling efficiency of the grating couplers used at optical input/output (I/O) interfaces [4], and

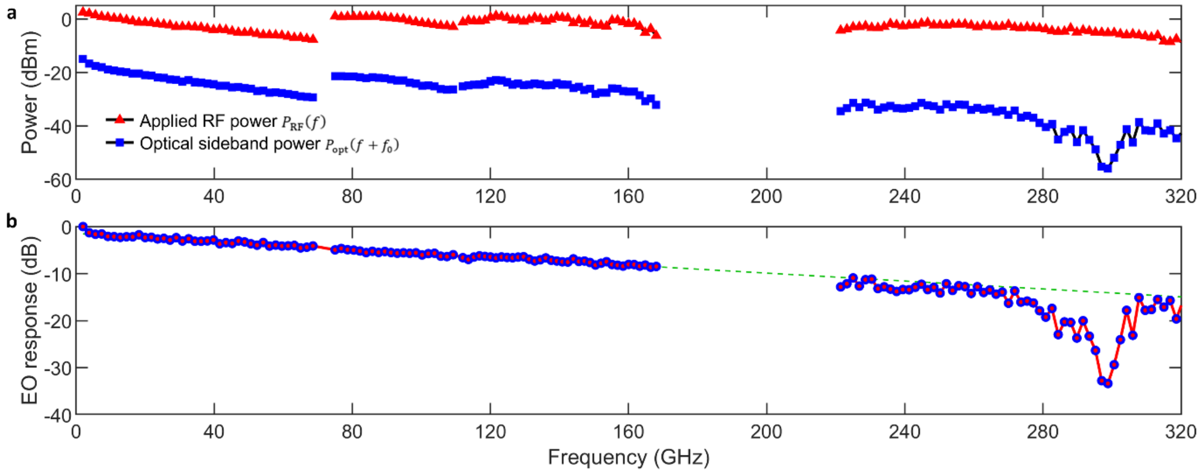

Fig. S3| Measurement of the MZM EO response. a Red markers: Radio-frequency (RF) power $P_{\mathrm{RF}}(f)$ of the test sinusoidal signals (frequency f) applied to the MZM electrodes, calculated based on the $|S_{21}(f)|^2$ power transmission of the probes and the RF power of the test signals measured at the input of the probe. Blue markers: Power $P_{\mathrm{opt}}(f+f_0)$ of the optical sideband, measured by an optical spectrum analyzer (OSA) at the MZM output. **b** EO transfer function $\widetilde{H}_{\mathrm{EO}}(f+f_0)$ of the MZM, normalized to its low-frequency value at $f=2\,\mathrm{GHz}$. The blue circles correspond to measurement points and indicate that the frequency response of the MZM essentially decays linearly. The green dashed line is a linear extrapolation of the lower frequency part. The strong dip in the measured transfer function at around 290 GHz results from the parasitic influence of a periodically structured back-reflector layer on the RF performance of the modulator. This back-reflector layer is intended to improve the coupling efficiency of the optical input/output grating couplers [4].

which unfortunately hamper RF signal propagation around 290 GHz. The empty range from 170 GHz to 220 GHz is due to the absence of a frequency multiplier operating in that specific frequency band. Nevertheless, the frequency response of the MZM exhibits an essentially linear roll-off, which allows to approximate the response in the empty frequency range through linear interpolation. In Fig. S3b, the green dashed line represents a linear extrapolation based on the response in the lower frequency range. The frequency roll-off of the employed MZM is approximately –15 dB at 320 GHz.

The roll-off in the EO response can be compensated in different ways. One approach would be to use a RF peaking amplifier before the MZM [20]. This would, however, require a dedicated amplifier design, which was beyond the scope of our current work. Another method is to compensate the roll-off in the optical domain, e.g., by means of a resonantly enhanced MZM [22] or by an adapted optical gain-flattening filter [21] that is used at the MZM output, e.g., after EDFA1, see Fig. S1a. Optical equalization has the advantage that only the essentially flat ASE noise spectrum introduced by the EDFA1 will be enhanced, while the subsequent ADC noise spectra remain unaffected. This approach is subject of ongoing research. In our experiments, we still relied on a purely digital compensation of the EO response. This concept is straightforward to implement and does not come with additional hardware complexity, but unavoidably leads to an amplification of high-frequency noise contributions and distortions of the acquired signal, including the noise of the electronic ADC. This results in a tilted and distorted noise floor in the reconstructed spectrum, as visible in Fig. S6. The noise floor tilts up by 15 dB at 320 GHz and exhibits a prominent peak around 290 GHz, which is the result of compensating the dip in the EO response of the MZM at that frequency, see Fig. S3b. In our case, the overall noise is dominated by the ADC in case of high RF input powers, and the ADC noise of the high-frequency signal slices ($\mu = 2,3,4$, see Fig. S1). Clearly, the unwanted amplification of ADC noise by digital compensation of the MZM transfer function would not occur for optical equalization, leaving room for further improving the SINAD performance and the ENOB.

In future implementations of photonic-electronic ADC, bandwidth limitations of the MZM can be mitigated by optimized device designs [9] or by using alternative modulator technologies. As an example, 3 dB bandwidths in excess of 360 GHz [3],[6] have been demonstrated by plasmonic-organic hybrid (POH) MZM, albeit at higher optical

loss and higher half-wave voltage U_π. Silicon-organic hybrid (SOH) devices might also reach 3 dB bandwidths well beyond 100 GHz at significantly smaller optical loss and half-wave voltages, either by using optimized doping profiles [5] or by relying on capacitively coupled device schemes [10]. In light of recent reports on photochemical stability of organic electro-optic materials and associated devices [7],[8], both POH and SOH MZM could represent attractive and compact alternatives to the currently used TFLN MZM.

S5. Calibration of the system opto-electronic (OE) response

To determine the opto-electronic (OE) transfer functions $\underline{\tilde{H}}_{\mathrm{OE},\mu}(f)$ from the MZM output, Point Ⓐ in Fig. S1a, to the ADC outputs, a one-time calibration measurement is performed using an optical reference waveform (ORW) with known amplitude and phase. The ORW is derived from an ultra-stable femtosecond laser (MENHIR-1550, Menhir Photonics AG, Glattbrugg, Switzerland), which generates a pulse train with a well-defined pulse shape and a small repetition rate of $f_{\mathrm{ORW,FSR}} \approx 250\,\mathrm{MHz}$, resulting in densely spaced optical comb lines in the spectral domain. The complex-valued spectral envelope $\underline{\tilde{A}}_{\mathrm{ORW}}(f)$ of the laser pulse is measured by the manufacturer using the frequency-resolved optical gating (FROG) technique and is shown in Fig. S4a [14]. To ensure accurate calibration, we select the spectrally flat central region of the ORW spectrum for the calibration measurement – this region is marked by red dot lines in Fig. S4a. Note that photonic-electronic ADC system must cover exactly this region by proper choice of the optical carrier frequency f_0.

To perform the calibration measurement, the ORW is fed to the optical receiver system in Point Ⓐ of the setup, see Fig. S1a, and four spectral slices of the ORW are coherently detected by the corresponding LO tones, resulting in frequency combs with line spacing of $f_{\mathrm{ORW,FSR}} \approx 250\,\mathrm{MHz}$ in the baseband. The associated RF tones contain both the amplitude and phase of the OE transfer functions $\underline{\tilde{H}}_{\mathrm{OE},\mu}(f)$. Using Eq. (S18) and replacing the signal spectrum $\underline{\tilde{A}}_{\mathrm{S}}(f)$ by the ORW spectrum $\underline{\tilde{A}}_{\mathrm{ORW}}(f)$, the detected baseband signals $\underline{\tilde{U}}_{\mathrm{cal},\mu}(f)$ can be expressed as

$$\underline{\tilde{U}}_{\mathrm{cal},\mu}(f) = \underline{\tilde{H}}_{\mathrm{OE},\mu}(f)\,\underline{\tilde{A}}_{\mathrm{ORW}}\left(f + f_{\mathrm{IF},\mu}\right) + \underline{\tilde{N}}_{\mathrm{Rx},\mu}(f) \tag{S45}$$

By numerically shifting the spectral slices to their proper frequencies $f_{\mathrm{IF},\mu}$ in the baseband according to Eq. (S19), we obtain the relation

$$\underline{\tilde{U}}_{\mathrm{cal},\mu}\left(f - f_{\mathrm{IF},\mu}\right) = \underline{\tilde{H}}_{\mathrm{OE},\mu}\left(f - f_{\mathrm{IF},\mu}\right)\underline{\tilde{A}}_{\mathrm{ORW}}(f) + \underline{\tilde{N}}_{\mathrm{Rx},\mu}\left(f - f_{\mathrm{IF},\mu}\right) \tag{S46}$$

We finally compare the measured signal slices with the known ORW at the input to retrieve the OE transfer functions,

$$\underline{\tilde{H}}_{\mathrm{OE,est},\mu}\left(f - f_{\mathrm{IF},\mu}\right) = \frac{\underline{\tilde{U}}_{\mathrm{cal},\mu}\left(f - f_{\mathrm{IF},\mu}\right)}{\underline{\tilde{A}}_{\mathrm{ORW}}(f)} \tag{S47}$$

Note that the received spectral slices are also impaired by independent AWGN $\underline{\tilde{N}}_{\mathrm{Rx},\mu}(f)$. Therefore, the transfer functions are extracted only from the peak points of the comb lines to mitigate the influence of noise. The number of samples obtained from the calibration measurement is thus dictated by the number of ORW tones, which are separated by the FSR of the ORW $f_{\mathrm{ORW,FSR}} \approx 250$ MHz. Still, the associated spectral sampling is sufficiently dense to capture the characteristics of the system and the transfer function $\underline{\tilde{H}}_{\mathrm{OE,est},\mu}\left(f - f_{\mathrm{IF},\mu}\right)$ at intermediate points between the ORW comb tones can be obtained by interpolation. Note that the retrieved OE transfer functions $\underline{\tilde{H}}_{\mathrm{OE,est},\mu}\left(f - f_{\mathrm{IF},\mu}\right)$ may also include an unknown frequency-independent and slowly time-variant factor, which is assumed to be constant during one calibration measurement but may vary from measurement to measurement. This factor, however, is of no practical consequence since the measurement of the optical waveform in the ADC experiment is anyway subject to an unknown frequency-independent and slowly time-variant factor that needs to be estimated during signal reconstruction, see Section S2.2.

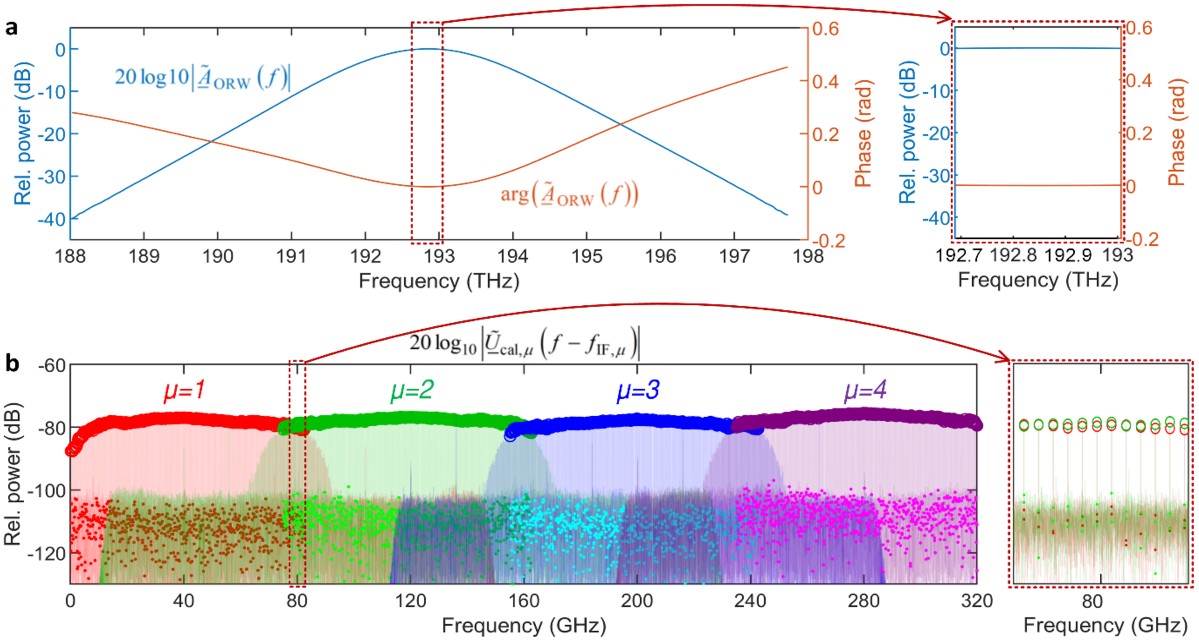

Fig. S4| Power spectra of received RF combs obtained from one calibration measurement. **a** The complex-valued spectral envelope $\tilde{\underline{A}}_{\mathrm{ORW}}(f)$ of the laser pulse measured by the manufacturer using the frequency-resolved optical gating (FROG) technique. **b** Circles: Peak points of the ORW comb tones spaced by $f_{\mathrm{ORW,FSR}} \approx 250\,\mathrm{MHz}$. The complex-valued amplitudes of these tones are used to extract the OE transfer functions $\tilde{\underline{H}}_{\mathrm{OE},\mu}(f)$ of the various detection paths. The gaps between the tones can be closed by appropriate interpolation. Dots: Spectral sampling points in neighbouring frequency bins in-between the ORW comb-tone peaks, representing a pretty uniform noise floor. This proves that spectral leakage can be effectively suppressed by our digital signal processing procedures, thereby permitting a precise measurement of the system OE transfer functions $\tilde{\underline{H}}_{\mathrm{OE},\mu}(f)$. The resolution bandwidth (RBW) of the plot is 1 MHz.

It should also be noted that the calibration measurement may be subject to spectral leakage since the baseband comb tones related to the ORW do generally not coincide with the frequency points that result from a discrete Fourier transform of the time-domain waveform obtained from the real-time-oscilloscope recordings. These leakage effects, however, can be mitigated by appropriate digital signal processing (DSP) techniques that are explained in the following. For each spectral slice μ, the ORW comb tones recorded in the baseband are located at discrete frequencies that can be described as the sum of an slice-specific offset frequency $f_{\mathrm{offset},\mu}$ and integer multiples of the ORW repetition frequency $f_{\mathrm{ORW,FSR}}$,

$$f_{\mathrm{ORW},\mu,m} = f_{\mathrm{offset},\mu} + m \cdot f_{\mathrm{ORW,FSR}}, m \in \mathbb{Z} \tag{S48}$$

The complex-valued signals $\tilde{\underline{U}}_{\mathrm{cal},\mu}(f-f_{\mathrm{IF},\mu})$ are then digitized by the back-end electronic ADC with a sampling rate $f_{\mathrm{samp}} = 256\,\mathrm{GSa/s}$ and a finite overall observation interval $T_{\mathrm{obs}} = N_{\mathrm{samp}}/f_{\mathrm{samp}} \approx 8\,\mu\mathrm{s}$, where N_{samp} indicates the number of recorded samples. This generally leads to spectral leakage of the otherwise sharp ORW comb tones, which can be mitigated by choosing a proper number of samples and by applying a frequency shift, such that both the offset frequency $f_{\mathrm{offset},\mu}$ and the repetition frequency $f_{\mathrm{ORW,FSR}}$ are integer multiples of the frequency bin size $f_{\mathrm{samp}}/N_{\mathrm{samp}}$. To this end, we first extract the exact period T_{ORW} and the associated repetition frequency $f_{\mathrm{ORW,FSR}} = 1/T_{\mathrm{ORW}}$ of the baseband ORW comb by calculating the autocorrelation of the recorded time-domain ORW waveform with relative delays of approximately 1000 ORW periods. This leads to a periodic pattern of autocorrelation peaks from which the exact period T_{ORW} can be extracted with an accuracy much better than the sampling period $T_{\mathrm{samp}} = 1/f_{\mathrm{samp}} \approx 4\,\mathrm{ps}$. Spectral leakage is caused by the fact that the obtained repetition frequency $f_{\mathrm{ORW,FSR}} = 1/T_{\mathrm{ORW}}$ is generally not an exact integer multiple of the frequency bin size $1/T_{\mathrm{obs}} = f_{\mathrm{samp}}/N_{\mathrm{samp}}$ of the originally recorded waveform. To alleviate this problem, we fine-tune the number N_{samp} of samples considered for further processing such that the extracted ORW repetition frequency $f_{\mathrm{ORW,FSR}}$ is as close as possible to an integer multiple of the

frequency bin size $1/\left(N_{\text{samp}}T_{\text{samp}}\right)$. To find the best number of samples, we minimize the offset $\left|f_{\text{ORW}}-M/\left(N_{\text{samp}}T_{\text{samp}}\right)\right|$ of the extracted ORW repetition frequency to its nearest frequency bin $M/\left(N_{\text{samp}}T_{\text{samp}}\right)$, where M is an integer number, and where the usable number of samples N_{samp} is limited to approximately 1 million (observation time $T_{\text{obs}}=N_{\text{samp}}/f_{\text{samp}}\approx 4$ µs) by the drift of the LO tones. In addition, the offset frequencies $f_{\text{offset},\mu}$ still do not match the frequency grid and thus remain as a problem. To mitigate this effect, we perform a second step, in which the baseband combs are numerically shifted by an additional frequency offset f_{shift} within the range of one frequency bin, f_{shift} in $\left[-f_{\text{samp}}/2k, f_{\text{samp}}/2k\right]$. This shift is implemented by a multiplication with a complex exponential of the form $\exp\left(\mathrm{j}2\pi f_{\text{shift}}t\right)$ in the time domain, and the exact value of f_{shift} is chosen to maximize the power ratio between the peak points of the digitally reconstructed ORW comb lines and their neighbouring spectral points, and hence minimizing the spectral leakage. An exemplary power spectrum $20\log_{10}\left|\underline{\tilde{U}}_{\text{cal},\mu}\left(f-f_{\text{IF},\mu}\right)\right|$ of four spectral slices obtained from a calibration measurement are shown in Fig. S4, where the spectral slices have already been displayed with respect to the corresponding respective frequencies $f_{\text{IF},\mu}$. The peak points of the comb lines are marked with densely spaced circles, representing a free spectral range $f_{\text{ORW,FSR}}$ of approximately 250 MHz. The neighbouring spectral sampling points around the comb-tone peaks are marked with dots, which are mainly buried in the noise floor. This demonstrates that the spectral leakage can be effectively mitigated through the digital signal processing procedures described above, enabling a precise readout of the amplitude and phase of the complex-valued transfer functions $\underline{\tilde{H}}_{\text{OE},\mu}\left(f\right)$ from the peak points of the comb lines.

S6. Frequency response of the photonic-electronic ADC

With the transfer functions $\tilde{H}_{\text{EO}}\left(f\right)$ and $\underline{\tilde{H}}_{\text{OE},\mu}\left(f\right)$ of the MZM and of the photonic-electronic signal-processing engine at hand, we finally arrive at the estimate $U_{\text{S,est}}\left(t\right)$ of the original broadband RF signal $U_{\text{S}}\left(t\right)$ as described in Eq. (S37). Still, the reconstructed signal may differ from the original input signal by an overall unknown amplitude scaling factor $\left|\underline{C}\left(\tau\right)\right|$ as described in Section S2.2. It should also be noted, that in the current implementation of our proof-of-concept system, this scaling factor may still differ from recording to recording, since the gain of the EDFA following the MZM (EDFA2 in Fig. S1) was adjusted to yield a constant average output power for each waveform, which was dictated by the maximum power rating of the subsequent WaveShaper (WS1 in Fig. S1). This decision was made to ensure maximum possible voltage swing at the output of the BPD, thereby minimizing the impact of the electronic noise added by the subsequent ADC. This effect is taken into account by decomposing the scaling factor into two real-valued factors parts. Therefore, we decompose the scaling factor into two parts:

$$\left|\underline{C}\left(\tau\right)\right|=C_{\text{EDFA}}\left(\tau\right)C_{\text{system}} \tag{S49}$$

where $C_{\text{EDFA}}\left(\tau\right)>0$ represents to the recording-dependent EDFA gain, while $C_{\text{system}}>0$ is a remaining scaling factor that is associated with the overall system. To reconstruct the waveform with the correct amplitude scaling, we must hence extract the recording-dependent EDFA gain $G_{\text{EDFA,dB}}>10\log_{10}\left(C_{\text{EDFA}}\left(\tau\right)\right)$ and at the same time determine the recording-independent system-related scaling factor C_{system}. Since our proof-of-concept setup may still be subject to drift, we newly extracted the system-related scaling factor C_{system} for each measurement campaign, typically after shutting down and re-starting the system. To this end, we feed the system with different RF test signals, extract the overall scaling factors $\left|\underline{C}\left(\tau\right)\right|$ and the respective recording-dependent EDFA gains $C_{\text{EDFA}}\left(\tau\right)$, and then take the average of the ratio of the two to obtain the system-related scaling factor,

$$C_{\text{system}}=\left\langle C_{\text{EDFA}}\left(\tau\right)/\left|\underline{C}\left(\tau\right)\right|\right\rangle \tag{S50}$$

where the sharp brackets '$\langle\ \rangle$' denote an average over the results obtained from all test signals.

With the system-related scaling factor C_{system} at hand, we can then perform a frequency sweep of the RF input signals, extract the recording-dependent EDFA gain for each test-signal, and finally obtain the reconstructed signal with the correct amplitude scaling. Taking the ratio of the reconstructed signal amplitude to that of the test signal leads to the frequency response shown in in Fig. 2d of the main manuscript. Note that we were lacking adequate signal sources in the range between 170 GHz and 220 GHz, leading to a gap in the measured frequency response. Still, the acquired data

points cover most of the frequencies and indicate a reasonably flat response. Remaining uncertainties are attributed to inaccuracies of the calibration measurement and/or the imprecise extraction of the EDFA gain. Overall, the reproducibility and flatness of the results demonstrate that our photonic-electronic ADC system exhibits an essentially flat frequency response of our system over a 320 GHz bandwidth, see Fig. 2c in the main paper, indicating that the amplitude of the digitized signal accurately represents the amplitude of the analogue input signal. In the Fig. 2e of the main paper, we show three digitized sinusoidal waveforms that were used for checking our calibration. The sinusoidals feature three frequencies of 56 GHz, 280.8 GHz, and 307.8 GHz with known RF powers of 6.1 dBm, 4.3 dBm, and 6.2 dBm, respectively. Assuming a 50 Ω impedance, these RF powers can be translated into known peak-to-peak voltages of 1.28 V, 1.04 V, and 1.30 V, respectively. On the other hand, the voltage swings can also be extracted from the sinusoidal fits of the digitized waveforms, see red traces in Fig. 2e of the main manuscript, relying on the conversion factors extracted in the calibration measurement. This leads to peak-to-peak values of 1.21 V, 1.07 V, and 1.38 V, respectively, which are in good agreement with the values extracted from the measured RF power. We believe that system drift and resulting uncertainties can be further mitigated by using photonic integrated circuits to implement the photonic-electronic signal-processing engine of our system [16][17].

S7. Effective number of bits (ENOB) analysis of the photonic-electronic ADC

After completing the calibration procedures explained in Sections S3 through S6, we have a fully functional photonic-electronic ADC system at hand. To estimate the performance of this system, we quantify the effective number of bits (ENOB). The ENOB is typically calculated from the signal-to-noise-and-distortion ratio (SINAD) of a digitized sinusoidal signal with an amplitude that covers the full-scale input range (FSIR) of the ADC system. To calculate the ENOB from the SINAD of a full-scale sinusoidal test signal, we adopt the relations defined by the IEEE standard 1241-2023 for electronic ADC [18], leading to the widely used relation

$$\mathrm{ENOB} = \frac{\mathrm{SINAD_{dB}} - 1.76}{6.02} \tag{S51}$$

In this relation, $\mathrm{SINAD_{dB}} = 10\log_{10}(\mathrm{SINAD})$ represents the dB value of the SINAD measured for a full-scale test tone. The ENOB serves as a quantitative measure of the resolution and accuracy of our photonic-electronic ADC system and is extracted as described in the following sections.

S7.1. Full-scale input range (FSIR)

To estimate the ENOB of our system, we need to define the full-scale input range (FSIR) and quantify the noises and distortions. Unlike a conventional electronic ADC, where the noises and the distortions are merely independent of the applied input signal, our system is subject to signal-dependent distortions, which limit the usable FSIR and eventually the ENOB. Specifically, the RF input signal is first applied to a front-end MZM, which is subject to a cosine-shaped electro-optic amplitude transfer characteristic, leading to third-order and possibly also to residual second-order intermodulation products, which impair the reconstructed waveform. Since the distortions related to these second- and third-order intermodulation products increase in proportion to the square and the cube of the signal, the SINAD will eventually be reduced as the signal power increases further, thereby leading to an optimum signal power swing for which the photonic-electronic ADC offers the highest SINAD. This signal power swing eventually defines the FSIR.

To measure the FSIR of our system, we drive it with a sinusoidal test tone at 2.0 GHz and sweep the voltage swing or, equivalently, the RF power of the drive signal. For each RF input power, we extract the various noise and distortion contributions, quantified by the ratio of the associated noise and distortion power to the power of the test tone, and calculate the resulting SINAD as the inverse of the sum of all these contributions. On a practical level, this is done by first obtaining the reconstructed waveform for each sinusoidal test tone and by then analysing its spectrum to identify the various noise and distortion contributions, see Section S7.2 below for a more detailed analysis. We repeat this analysis for different RF powers of the test tone at 2.0 GHz, the corresponding results are shown in Fig. S5a. For low RF powers, the ASE noise (‘ASE & Others’) of the EDFA (EDFA1) represents the dominant limitation, whereas the noise of the electronic ADC (‘el. ADC’) and the second- and third-order harmonics resulting, e.g., from MZM nonlinearities (‘Harmonics’) are much weaker, see orange, black, and green curve in Fig. S5a. Note that orange trace

also contains some remaining distortions (‘Other’) such as IQ crosstalk, signal-signal beating interference (SSBI) in the various balanced detectors, as well as stitching tones and clock tones (Clk. tones) from the oscilloscope, which are significantly weaker than most other distortions, see exemplary reconstructed spectra in Fig. S6, and which are therefore not separated out. When increasing RF powers, the relative power of the third-order harmonics caused by the MZM increases rapidly, and nonlinear distortions (green curve) eventually evolve into a dominant limitation of the SINAD. A maximum SINAD of 17.8 dB is reached at an RF input power of 12.5 dBm, corresponding to a full-scale peak-to-peak voltage swing of 2.67 V, see dashed lines in Fig. S5a. Note that we deliberately chose a relatively low test-tone frequency of 2.0 GHz for extracting the FSIR for several reasons: First, MZM-related nonlinear distortions, which limit the FSIR, will become weaker at higher frequencies, as the EO frequency response of the MZM decreases, see Fig. S3b. Test tones at high frequencies would hence lead to an overestimation of the FSIR and hence of the ENOB. Second, the SINAD characterization requires a high-quality signal source that can drive the MZM over its full range without introducing too many distortions itself, which is straightforward at low frequencies, but challenging at high frequencies.

S7.2. Signal-to-noise-and-distortion ratio (SINAD) and effective number of bits (ENOB)

With the determined FSIR, the ENOB at different frequencies can be then extracted by determining the SINAD at the FSIR. For low input-frequency scenarios, where high-power sources are available, the SINAD can directly be measured by driving our system with a sinusoidal with a peak-to-peak swing of 2.67 V that fully covers the FSIR. The SINAD of 17.8 dB obtained for the full-range sinusoidal at 2.0 GHz, Fig. S5a, corresponds to an ENOB of 2.7, and measurements at 5.6 GHz and 9.2 GHz using the same test tone power lead to comparable SINAD values of 17.1 dB, see red circles in Fig. S5b and Fig. S5c, corresponding to an ENOB of 2.6. The slightly decreased SINAD at 5.6 GHz and 9.2 GHz as compared to 2.0 GHz is attributed to the roll-off of the system transfer function towards lower frequencies, caused by the low-pass behaviour of the IQ receiver associated with the first slice (IQR1). For a given RF power of 12.5 dBm, corresponding to the FSIR, the 2.0 GHz test tone hence leads to a lower power of the electrical output signal of IQR1 as compared to the 5.6 GHz and 9.2 GHz tones. Since the range of the corresponding electronic ADC is always adapted to the swing of the input signal, the spectrally white ADC noise background associated with the 2.0 GHz test tone will be lower than the noise background associated with the 5.6 GHz and the 9.2 GHz tones. In both cases, the ADC noise background is subject to the same digital compensation of the system transfer function, such that the ADC-related impairments associated with the acquisition of the 5.6 GHz and the 9.2 GHz signal are slightly higher than the ADC-related impairments for the 2.0 GHz signal. This can be seen by observing the fact that the levels of the relative electrical ADC noise (‘el. ADC’) for the 2.0 GHz signal, see Fig. S5a, is lower than the ADC noise for the 5.6 GHz and the 9.2 GHz signal, see Fig. S5b and S5c.

At increasing input frequencies, the frequency-dependent decay of the MZM transfer function becomes more and more relevant, which decreases the impact of MZM nonlinearities (‘Harmonics’). In addition, it becomes more and more difficult and eventually even impossible to directly measure the SINAD for a full-range input signal with a peak-to-peak swing of 2.67 V due to the lack of spectrally pure signal sources with sufficiently high output power. We therefore had to rely on an extrapolation of the various distortions measured at low RF input power towards higher powers, which is illustrated in Fig. S5d, Fig. S5e and Fig. S5f. To this end, we performed a semi-analytical simulation based on the simplified sketch shown in Fig. S1 by using component characteristics that were either measured directly in our experiment or adopted from the corresponding data sheets. More specifically, measured characteristics comprise the EO response of the MZM, the insertion losses of various optical components, the spectral transmission characteristics of the fibre Bragg grating (FBG), of the bandpass filter (BPF) and of the various frequency-dependent transfer functions related to the signal paths from the input of WS1 to the digitized signal output in the DSP, as well as the electrical ADC noise power of our high-speed oscilloscopes at different voltage scales. Similarly, the amplified LO comb is emulated based on the optical carrier-to-noise power ratio (OCNR) measured is our experiments, whereas the half-wave voltage of the MZM and the noise figures of the various EDFA in the setup are taken from the respective data sheets. In a first step, we verify the reliability of the model by applying it to the cases of low-frequency input signals and by verifying that the predicted noise and distortion levels, indicated by the solid lines in Fig. S5a, Fig. S5b

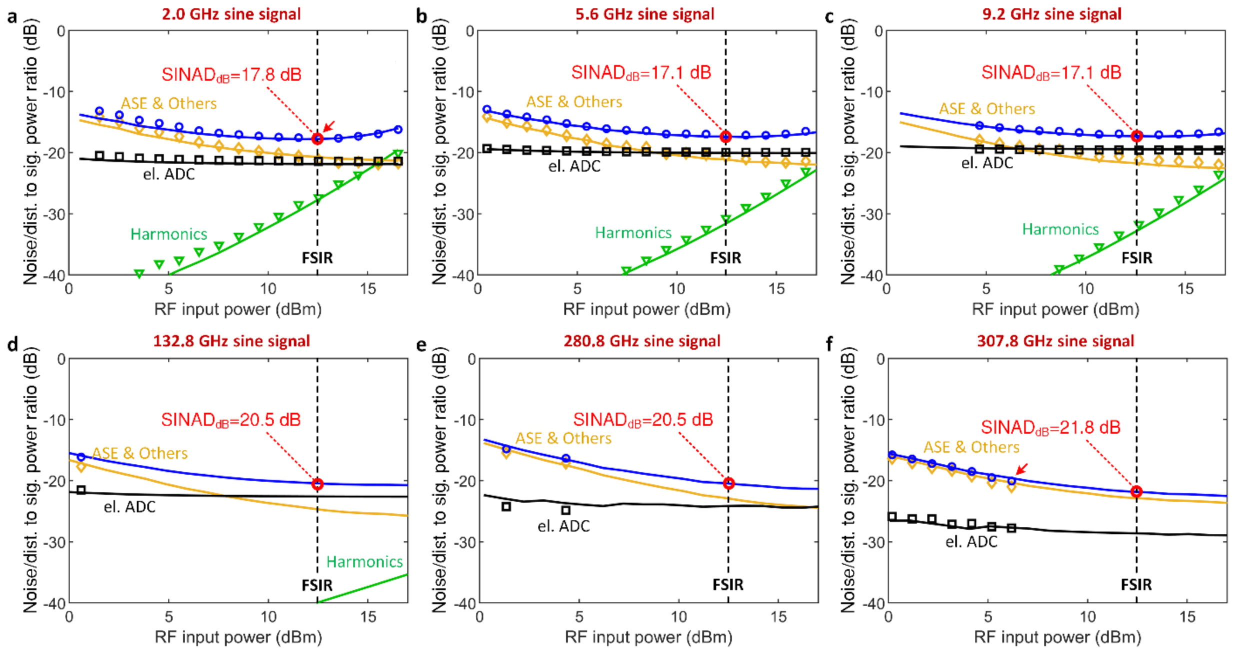

Fig. S5| Estimation of the full-scale input range (FSIR) and of noise and distortions in our photonic-electronic ADC. The plots show the ratios of the powers of various noise and distortion contributions to the signal power as a function of the RF power of an analogue sinusoidal test tone that is coupled to the MZM. The ratios extracted from experimental measurements are labelled by discrete markers. Green triangles: Ratio of the power of nonlinear distortions to the signal power ('Harmonics'). Black squares: Ratio of the power of electronic ADC noise to signal power ('el. ADC'). Yellow diamonds: Ratio of the ASE noise and remaining distortions to the signal power ('ASE & Others'). Blue circles: Ratio of the overall noises and distortions to the signal power ratio. Red circles indicate the SINAD$_{dB}$ at the full-scale input range (FSIR), which is marked by vertical dashed lines and which is defined to correspond to the RF input power that leads to maximum SINAD at a test-tone frequency of 2 V. The solid lines with associated colours are obtained from a dedicated semi-analytical simulation, that relied on a combination of directly measured system characteristics and component specifications obtained from the various data sheets. The good coincidence of the predicted and the measured noise and distortion levels confirms the reliability of the model. **a** Sinusoidal test tone at 2.0 GHz. The FSIR of our system is found at an RF input power of 12.5 dBm, which leads to maximum SINAD and which is indicated by the vertical dashed lines. **b,c** Sinusoidal test tones at 5.6 GHz and at 9.2 GHz. For these frequencies, the power levels of the FSIR signals can still be reached, and hence the ENOB can be directly measured. **d,e,f** Sinusoidal test tone at 132.8 GHz, 280.8 GHz, and 307.8 GHz. Even though we could not directly measure the ENOB due to the lack of a high-quality signal source with sufficiently high output power, we could verify that the simulation predictions are in line with the noise and distortion levels that could be directly measured at low input powers. This gives us confidence to use the mode for quantifying the noise and distortion levels also at higher input powers. The red arrows in Subfigures a and f indicate the data points that correspond to the spectral shown in Fig. S6 below.

and Fig. S5c, coincide very well with the measured characteristics, represented by discrete circular, triangular, rectangular, and diamond-shaped markers. In a second step, the thus verified model is used to estimate the system performance at higher frequencies of 132.8 GHz, 280.8 GHz, and 307.8 GHz, see Fig. S5d, Fig. S5e, and Fig. S5f. For all these frequencies, we again verify that the simulation predictions, solid lines, agree with the noise and distortion levels that could be directly measured at low input powers – the associated discrete measurement points are indicated by blue circles, orang diamonds, and black squares in Fig. S5d, Fig. S5e, and Fig. S5f. The good agreement of these measurement points with the model predictions gives us confidence that the simulated SINAD levels are also reliable at higher input powers. Assuming an input signal with a peak-to-peak swing of 2.67 V that covers the full FSIR, we find SINAD levels of 20.5 dB, 20.5 dB, and 21.8 dB for the considered frequencies of 132.8 GHz, 280.8 GHz, and 307.8 GHz, respectively. This corresponds to ENOB values of 3.1, 3.1 and 3.3, respectively. As expected, these ENOB levels are higher than the ENOB of 2.7 found for the 2.0 GHz test tone due to the reduced impact of MZM nonlinearities and electrical ADC noise. As expected, the impact of MZM nonlinearities ('Harmonics') becomes less and less important for higher frequencies, while the decreasing input power of the modulation sideband into EDFA1 renders ASE noise the dominant impairment.

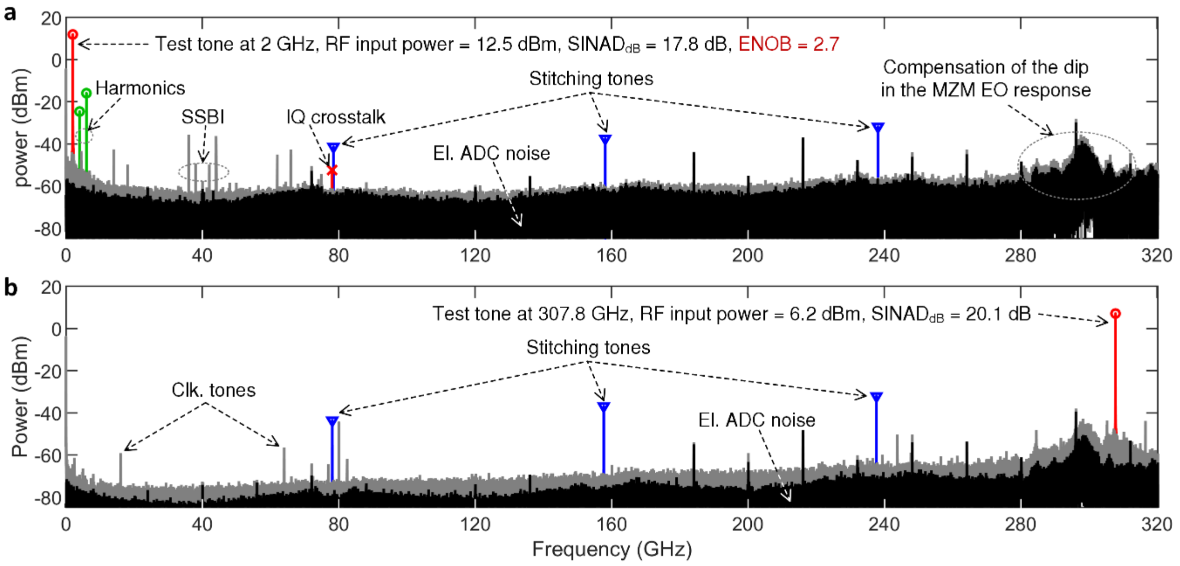

Fig. S6| Spectrum of exemplary reconstructed sinusoidal signals with frequencies of 2 GHz and 307.8 GHz, respectively, corresponding to the data points marked by red arrows in Fig. S5a and c. The fundamental tones are marked in red with circular marker at the peak points. In Subfigure (a), the IQ crosstalk of the fundamental tone is also marked in red, but with a 'x' marker at the peak point, and the second and third-order harmonics of the fundamental tone are marked in green. The plots also reveal the optical stitching tones (blue), which are added at the spectral overlap regions and which help in amplitude and phase stitching, see Section S2.2. The grey part of the spectrum comprises all other noise and distortion contributions, including signal-signal beat interference ('SSBI'), clock tones of the underlying electronic ADC ('Clk. Tones') as well as ASE noise of the various EDFA in the optical setup and electrical ADC noise. The frequency-dependence of the ASE and ADC noise background is caused by digital compensation of the system transfer functions $\underline{\widetilde{H}}_{\mathrm{OE},\mu}(f)$ and of the EO response $\widetilde{H}_{\mathrm{EO}}(f)$ of the MZM. Specifically, the increased noise around 290 GHz is caused by a strong dip of the MZM transfer function at this frequency, see Fig. S3b above. The black graph is a separately plotted spectrum, which indicates only the electrical noise and which is dominated by the ADC noise.

For a better understanding of the various noise and distortion contributions, we depict in Fig. S6 the spectrum of two exemplary reconstructed sinusoidal signals – one with a 12.5 dBm input power level at 2.0 GHz, Fig. S6a, and one with a 6.2 dBm input power level at 307.8 GHz, Fig. S6b. The two signals correspond to data points marked by red arrows in Fig. S5a and Fig. S5f, respectively. In Fig. S6a, the fundamental test tone is marked in red with a circular marker at the peak, while the tone related to IQ crosstalk of the test tone is labelled with a red 'x' marker and while the second-order and third-order harmonics of the fundamental tones are marked in green. For the 307.8 GHz-signal in Fig. S6b, no IQ crosstalk is visible due to better IQ balancing, and higher harmonics are also absent since they fall outside of the detection bandwidth of the system. We additionally include the optical stitching tones indicated in blue in Fig. S6, which are positioned in the spectral overlap regions between neighbouring slices and which help in amplitude and phase stitching of neighbouring slices, see Section S2.2. The remaining noises and distortions are indicated in grey, comprising, e.g., spurious clock tones (Clk. tones) of the back-end electronic ADC and a broadband noise background. In Fig. S6a, signal-signal beating interference (SSBI) appears as an additional impairment which results from non-ideal balanced photodetectors and which leads to beat notes of the test tone with the remaining carrier and with the lower modulation sideband. With respect to the broadband noise background, the spectrum in grey comprises all the contributions of ASE noise and electrical ADC noise. Notably, this noise appears to slightly increase in the vicinity of the respective test tone – we attribute this effect to the beating of the single-frequency test tone with the band-limited ASE noise of the LO tones, which is caused by amplification and subsequent spectral filtering of the underlying frequency comb by EDFA2 and WS2, see Fig. S1. The spectrum plotted in black in Fig. S6 refers to the electrical noise of the ADC only, which was obtained by disconnecting all optical inputs while keeping the same ADC settings and the same DSP that was used for signal reconstruction. This noise background is dominated by noise of the ADC and exhibits a prominent broad peak around 290 GHz, which is a consequence of compensating for the dip in the EO response of the MZM at that frequency, see Fig. S3b. The contribution labelled as 'el. ADC' in Fig. S5 is

obtained by calculating the overall power of the electrical noise background that is indicated in black in Fig. S6a and Fig. S6b.

S7.3. Further improvement in ENOB

With the understanding of the dominant noise and distortion contributions as explained in Section S7.2, we find substantial room for improving the ENOB of our photonic-electronic ADC system. Specifically, the SINAD values obtained in our experiments were mainly limited by the digitally amplified ADC and ASE noise power around 290 GHz, where the MZM transfer function exhibits a strong dip, see Fig. S3b. This limitation can be overcome by using an improved MZM, which does not exhibit such a dip and does hence not require noise-enhancing digital compensation. In our current device, the dip can be related to perturbations caused by periodic buried metal structures [4] that can be avoided, see Inset 1 of Fig. S8. To estimate the performance of a thus improved system, we modify the digitally compensated EO response of the MZM by using a linear interpolation between 273.6 GHz and 307.8 GHz instead of the measured transmission dip – the resulting transfer characteristics thus corresponds approximately to the dashed green line in Fig. S3. We then use the thus modified model to obtain the SINAD associated with the sinusoidal test tones discussed in Sections S7.1 and S7.2. The FSIR is again extracted from the sinusoidal test tone at 2.0 GHz and now corresponds to an RF input power of 9.5 dBm, see the vertical dashed line in Fig. S7a. Using this number, the maximum SINAD values for the 2.0 GHz, 5.6 GHz and 9.2 GHz test tones are improved from 17.8 dB, 17.1 dB and 17.1 dB to 21.6 dB, 21.4 dB and 20.6 dB, respectively, as indicated by red circles in Fig. S7a. This corresponds to improved ENOB values of 3.3, 3.3 and 3.1, and the ENOB values for the higher-frequency test signals would improve accordingly. Moreover, the EO response of the MZM can at least partially be compensated on a hardware level, avoiding again digital amplification of acquired noise. This can, e.g, be accomplished by an RF peaking

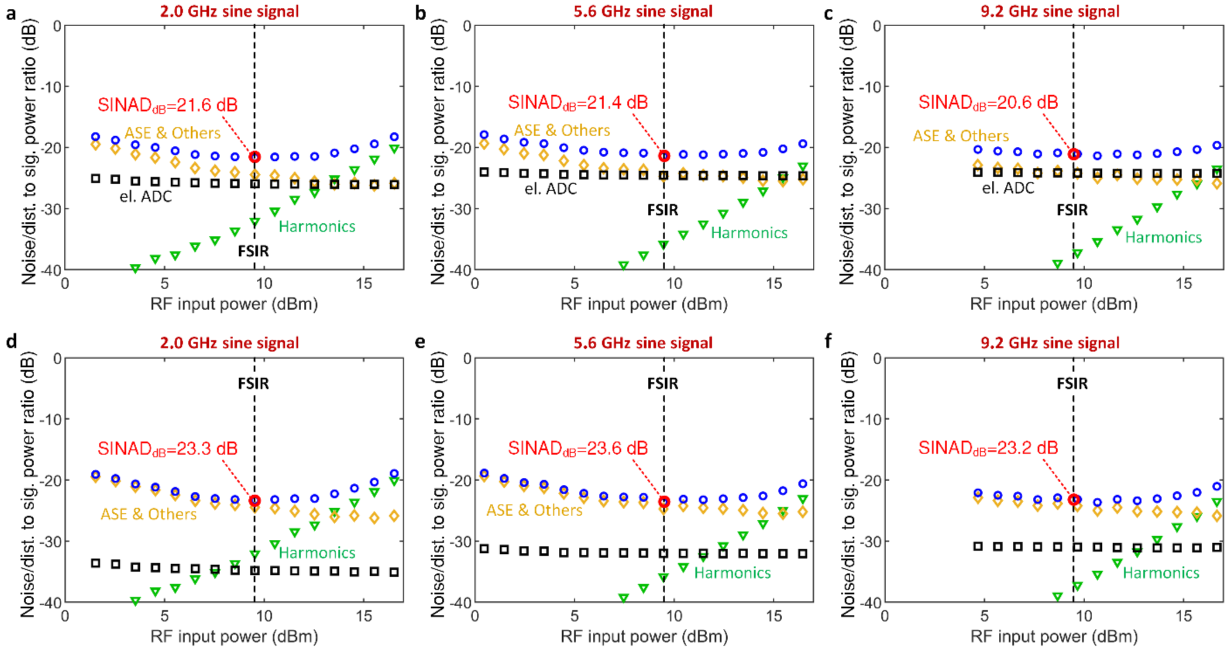

Fig. S7| Estimation of the full-scale input range (FSIR) and of noise and distortions for an improved implementation of our photonic-electronic ADC. The plots show again the ratios of the powers of various noise and distortion contributions to the signal power as a function of the RF power of an analogue sinusoidal test tone that is coupled to the MZM. **a,b,c** Results obtained for assuming that the transmission dip in the EO response of MZM, see Fig. S3b, can be avoided. To this end, we modify the digitally compensated EO response of the MZM in our model by using a linear interpolation between 273.6 GHz and 307.8 GHz. This leads to an improvement of the achievable SINAD by more than 3.5 dB as compared to the result shown in Fig. S5a, S5b, and S5c. **d,e,f** Results emulated by further assuming that the currently used digital compensation of the EO response of the MZM is accomplished in the optical domain by using an appropriate optical filter. The results show that the SINAD values can be improved further by approximately 2 dB.

amplifier at the input port of the MZM, taking, e.g., advantage of millimetre-wave integrated circuits that leverage III-V-based high-electron mobility transistor (HEMT) technology [20]. Alternatively or additionally, compensation can be achieved in the optical domain by using an appropriate optical filter [21] after EDFA1, see Fig. S1. This filter should be designed to undo the frequency-dependent decay of the MZM in the resulting optical output spectrum and could be implemented into the setting of WS1. The optical compensation would ensure that only the presumably flat ASE noise floor introduced by the EDFA1 will be effectively enhanced, while the currently used digital compensation increases both the ASE and the ADC noise floor. We also emulate this effect for the 2.0 GHz, the 5.6 GHz and the 9.2 GHz test tones by taking into account the digitally enhanced ASE noise contribution as extracted in Fig. S7a while the ADC noise contribution was taken from the stitched noise floor without applying the digital compensation of the EO transfer function. This leads to the results shown in Fig. S7b, for which the SINAD values are further improved to 23.3 dB, 23.6 dB and 23.2 dB for the 2.0 GHz, the 5.6 GHz, and the 9.2 GHz test tones, respectively. This corresponds to an ENOB values of 3.6, 3.6, and 3.6, respectively, and the ENOB values for the higher test-tone frequencies would increase accordingly. In addition to these measures, the third-order intermodulation products can be reduced by appropriate digital compensation techniques or by engineering the transfer function of the MZM, e.g., using ring-assisted Mach-Zehnder interferometer (MZI) structures, which can increase the linear range without dramatically increasing the half-wave voltage [22]. Moreover, the performance of the photonic-electronic ADC system can be further improved by using a better LO comb with higher power per comb line and hence higher OCNR, which will reduce the slightly increased noise in the vicinity of the respective test tones caused by the beating of the signal with the bandwidth-limited ASE noise of the amplified LO tones, as shown in Fig. S6. The impact of electrical noise could finally be reduced by using ADC with lower bandwidth and higher ENOB [30] – at the expense of increased hardware effort to implement a higher number of receiver channels for a given overall acquisition bandwidth. Based on these considerations, we feel that the proposed photonic-electronic ADC scheme bears significant room for further improving the signal quality while maintaining bandwidths of hundreds of GHz.

S8. Acquisition of broadband data signals

To further prove the viability of our photonic-electronic ADC concept, we use our experimental system to acquire a broadband analogue signal consisting of a 30 GBd 32QAM waveform centred at 24.4 GHz, a 40 GBd QPSK waveform centred at 233.4 GHz, and a 10 GBd 16QAM waveform centred at 264.4 GHz, see Fig. 3 of the main manuscript for details. Note that currently available RF connectors cannot support the entire 320 GHz acquisition bandwidth of our system. To overcome these limitations, we generate different spectral parts of the broadband signal electrically and

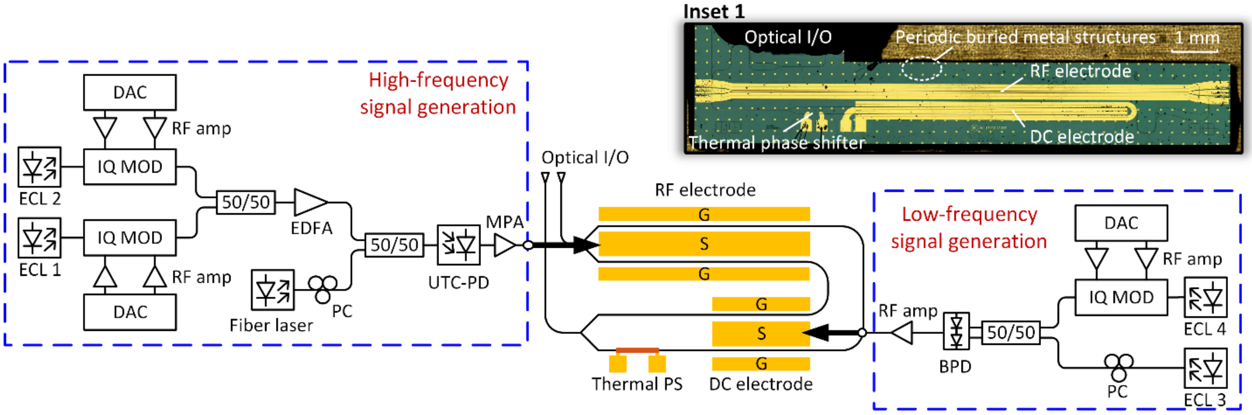

Fig. S8| Experimental setup for ultra-broadband signal acquisition in our proof-of-concept experiments. To overcome the bandwidth limitations of currently available RF connectors, we generate different spectral parts of the broadband test signal separately and couple them to different sets of electrodes of the Mach-Zehnder modulator (MZM). For both spectral parts, we exploit opto-electronic techniques for signal generation, where we first generate corresponding optical data signals and then down-converted them to the electrical domain by using either a uni-travelling-carrier photodiode (UTC-PD) or a standard high-speed balanced photodiode (BPD). At the input of the photonic-electronic ADC, the thus synthesized signals are simultaneously modulated onto a single optical carrier through two sets of MZM electrodes to emulate an ultra-broadband analogue input signal spanning the whole 320 GHz acquisition bandwidth of our system.

couple them to different sets of electrodes of the MZM, see Fig. S8 for the underlying experimental setup. The MZM comprises one electrode segment for broadband millimetre-wave signals (‘RF electrode’) and one segment for low-frequency signals (‘DC electrode’), which are illustrated in Inset 1 of Fig. S8 using a top-view microscope image of the MZM chip used in our experiment. For generating the two spectral parts of the test signal, we also relied on opto-electronic techniques: In a first step, we use electro-optic modulators to generate associated optical data signals, which are the down-converted to the electrical domain by appropriate photodiodes in a second step. Specifically, for the 40 GBd QPSK signal and the 10 GBd 16QAM signal located in J-band (220 – 325 GHz), the optical data signals were generated by using two IQ modulators, each fed by an external cavity laser (ECL1, ECL2 in Fig. S8) and driven by a pair of high-speed digital-to-analogue convertors (DAC), see left part of Fig. S8. The resulting signal is amplified and combined with a continuous-wave local-oscillator (LO) tone provided by a fibre laser before being fed to a J-band uni-travelling-carrier photodiode (UTC-PD, NTT Electronics Inc., Tokyo, Japan) for down-conversion. The power levels of the signal and the LO are set to be approximately equal, such that the maximum power of the resulting THz beat signal is obtained for a given maximum total optical input power of the UTC-PD. The THz signal is then amplified by an H-band medium-power amplifier (MPA, Fraunhofer Institute for Applied Solid-State Physics, Freiburg, Germany) and applied to the RF electrodes of the MZM via a THz waveguide probe (WR3, 220 − 325 GHz, Formfactor Inc., CA, United States). The 30 GBd 32QAM waveform centred at 24.4 GHz is generated in an analogous way, but we exploit a standard balanced photodetector (BPDV21xoR, 43 GHz, Finisar Corp., CA, USA) with coaxial output instead of a waveguide-coupled UTC-PD, see right part for Fig. S8. The resulting electrical signal is amplified and applied to the DC electrodes of the MZM via a coaxial probe (Infinity DC − 110 GHz, Formfactor Inc., CA, USA). Note that the DC electrodes were originally designed for applying a DC bias to the MZM, but still offer a decent bandwidth of a few tens of GHz. The EO response of the second electrodes is characterized in a separate measurement similar to the procedure described in Section S4 and additionally compensated for in the digital domain. Both the high-frequency and the low-frequency signal segments are simultaneously modulated onto the optical carrier to emulate an ultra-broadband analogue input signal spanning over the sub-THz acquisition bandwidth of our system.

The spectrum of the reconstructed broadband signal is shown in Fig. 3 of the main paper. Note that the analogue electrical signals generated by down-conversion of associated optical signals are usually subject to unknown phase, which may in addition drift over time due to the phase-noise of the underlying free-running light sources and which renders direct comparison of generated and reconstructed time-domain waveforms difficult. We therefore deliberately used quadrature-amplitude-modulated (QAM) data signals, for which phase drift and other linear distortions can be easily compensated by digital phase recovery and equalizer techniques and for which metrics like the bit error ratio (BER) can be used to quantify signal quality. Figure 3 of the main manuscript also indicated the constellation diagrams of the various data signals, which exhibit an appreciable increase of noise towards the outer constellation points. This hints to multiplicative noise, which we attribute to a limited optical carrier-to-noise power ratio (OCNR) of the underlying comb, see Section S9.1 below. Note that the data transmission experiment leading to the results shown in Fig. 3 of the main manuscript was performed using a Kerr soliton comb with a rather low $\mathrm{OCNR_{dB}}$ of 22.6 dB. For the ENOB measurements discussed in Section S7 above, the $\mathrm{OCNR_{dB}}$ was improved to values between 28.6 dB and 30.5 dB, see Section S9.1. The OCNR is defined for each comb line individually and refers to the ratio of the comb-line power and the noise power in a 12.5 GHz wide reference bandwidth, corresponding to a 0.1 nm wide frequency interval at a centre wavelength of 1550 nm.

S9. Characteristics of the Kerr soliton microcomb

S9.1. Optical carrier-to-noise ratio (OCNR)

High optical carrier-to-noise ratios (OCNR) of the Kerr soliton comb lines are key to high signal quality and to further scaling the acquisition bandwidth of the proposed photonic-electronic ADC scheme. In the experiments described in the main manuscript, the main OCNR limitation was caused by the rather low comb-line power provided by the frequency-comb generator (FCG). Specifically, the Kerr-comb resonator used for obtaining the results shown in Fig. 3 of the main manuscript was designed close to critical coupling and offered a per-line power in the range from −26 dBm to −25 dBm, whereas the device used for acquisition of the sinusoidal test signals in Fig. 2e of the main manuscript

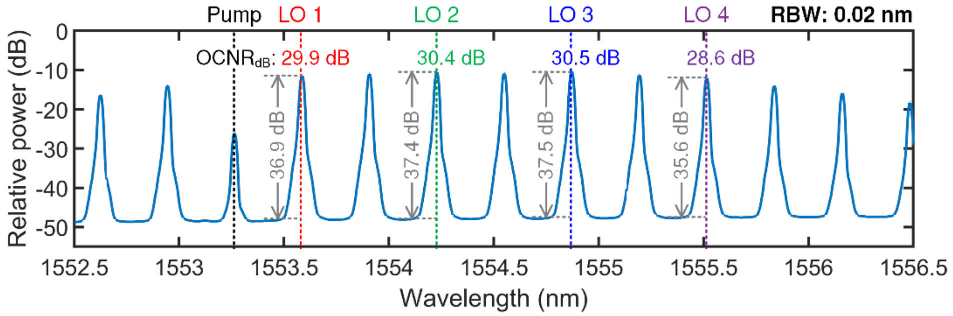

Fig. S9| Optical spectrum of the Kerr soliton comb measured after EDFA 4 shown in Fig. S1. The selected comb lines are marked by dashed lines. To reliably identify the constant noise background in between the much stronger comb tones, the spectrum was taken at an OSA resolution bandwidth of 2.5 GHz (0.02 nm at a wavelength of 1550 nm), whereas the OCNR is defined with respect to a reference noise bandwidth of 12.5 GHz (0.10 nm at a wavelength of 1550 nm). This difference has to be taken into account when translating the peak-to-floor ratios of 36.9 dB, 37.4 dB, 37.5 dB, and 35.6 dB indicated in the figure to the corresponding OCNR values of 29.9 dB, 30.4 dB, 30.5 dB, and 28.6 dB, respectively.

was over-coupled, leading to higher line powers in the range from −19 dBm to −20 dBm. In both cases, the comb had to be boosted by an erbium-doped fibre amplifier (EDFA4 in Fig. 2a of the main manuscript) before being split and fed to the IQR. This unavoidably leads to amplified spontaneous emission (ASE) noise, which limits the OCNR of the LO comb. Figure S9 shows an exemplary optical spectrum of the comb obtained from the over-coupled ring resonator, taken at the output of EDFA4 in Fig. S1. The dashed lines mark the selected comb lines that are used for the demonstration of single-tone acquisition shown in Fig. 2e and discussed in Section "Acquisition of sinusoidal test signals" in the main manuscript. Note that, to reliably identify the constant noise background between the much stronger comb tones, the spectrum was taken at an OSA resolution bandwidth of 2.5 GHz (0.02 nm at a wavelength of 1550 nm), whereas the OCNR is defined with respect to a reference noise bandwidth of 12.5 GHz (0.10 nm at a wavelength of 1550 nm). This difference has to be taken into account when translating the peak-to-floor ratios of 36.9 dB, 37.4 dB, 37.5 dB and 35.6 dB indicated in Fig. S9 to the corresponding OCNR values of 29.9 dB, 30.4 dB, 30.5 dB, and 28.6 dB, respectively. Note that a portion of the ASE noise could be still suppressed by optical bandpass filters (BPF) as shown in Fig. S1 after WS2. Still, spectral noise components in the direct vicinity of the respective comb tones remain and mix with the signal during coherent detection. This degrades the signal quality and leads to multiplicative noise in our demonstration of broadband signal acquisition, see Section S8 above.

Clearly, increasing the OCNR of the LO-comb is key to improving the performance of the photonic-electronic ADC system. This requires FCG with higher comb-line powers, which can be achieved in different ways: First, the coupling between the micro-resonator and bus waveguide can be increased further, allowing even more pump power to be transferred into the resonator for conversion to the Kerr comb, which increases the comb-line power at the output [46]. We have shown in one of our previous publications [44] that comb-line powers of –13.4 dBm and OCNR values beyond 35 dB can be achieved in experiments. However, increasing the coupling the also leads to higher threshold pump power and ruins the power-conversion efficiency of the FCG, leading to values below 1 % [44]. This problem can be overcome by using an auxiliary ring resonator with a large FSR to selectively enhance the coupling only for the pumped resonance [45]. This increases the conversion efficiency to 54 % and leads to experimentally demonstrated comb-line powers of −13 dBm, which may be increased to 0 dBm according to simulations [45]. Alternatively, dark soliton pulses offer a path towards high comb-line powers [46], [47]. Combining this approach with an auxiliary ring for coupling of the pump tone, comb-line powers above –6 dBm have been achieved in conventional waveguide-based devices [48]. More recently, tantalum pentoxide (Ta_2O_5) photonic-crystal resonators have been shown to support dark-pulse soliton combs with conversion efficiencies beyond 65 % and comb-line powers around 0 dBm [49]. Based on these impressive advances, we believe that OCNR levels close to 50 dB might be achievable in the future, allowing for ultra-broadband microcomb-based photonic-electronic ADC, which are not limited by the OCNR of the underlying comb source.

S9.2. Phase noise and timing jitter

Another important quality metric of Kerr comb is the relative phase drift between the comb lines or, equivalently, the timing jitter, which can impact the performance of the proposed photonic-electronic ADC system. To get a quantitative understanding of the jitter levels offered by our current comb source, we measure the RF phase noise of the pulse repetition rate using the setup shown in Fig. S10a. The Kerr soliton comb is generated by pumping the high-Q Si_3N_4 ring resonator using a tuneable laser source (TLS, TLB6728, Newport Corp., CA, United States). The power of the laser is amplified by an EDFA, which followed by a narrow bandpass filter for suppressing the unwanted ASE noise. At the output of the ring resonator, an optical notch filter is used to suppress the strong residual pump and the surrounding ASE noise. The Kerr soliton comb is then sent to a high-speed photodetector (XPDV3120R, Finisar Corp., CA, United States), which produces an RF beat note at the pulse repetition frequency of f_{rep} = 40.025 GHz. The phase noise of the RF beat note is then measured by a phase noise analyser (FSWP50, Rohde & Schwarz GmbH., Germany). The single-sideband (SSB) phase-noise power spectral density of the measured electrical beat note is plotted in Fig. S10b over a frequency range from 1 kHz to 10 MHz. The impact of phase noise on the acquisition performance of an ADC system depends on the length of the acquired signal, which we assume to be T_{acq} = 10 µs – a typical recording length for state-of-the-art high-speed real-time oscilloscopes [28]. For this recording length, the relevant root-mean-square (RMS) timing jitter of the LO comb can be calculated by integrating the single-sideband phase noise power spectrum from a lower limit of $f_1 = 1/T_{acq}$ = 100 kHz to an upper limit of typically half the repetition frequency, $f_2 = f_{rep}/2$. Assuming that the timing jitter of the Kerr comb or, equivalently, the phase noise of the generated RF beat note follows a non-stationary Wiener process, the associated power spectrum would be expected to decay with increasing frequency in proportion to $1/f^2$, as indicated in green dashed line in Fig. S10b. On the other hand, any practical measurement of optical phase noise is subject to a spectrally white background, which may originate from the limited sensitivity of the measurement equipment, from the electronic detector noise and/or from residual ASE noise, and which eventually masks the decay of the phase-noise power spectrum. In our case, the optical power of the soliton comb is distributed over many tones, and the associated fundamental RF beat note is thus rather low. This renders the sensitivity level of our phase-noise analyser the dominant limitation. Specifically, we obtain an RF power of −16.2 dBm at the input of the PNA, leading to a sensitivity level of −135 dBc Hz^{-1}, estimated from the specifications of the manufacturer [32], see the red dashed line in Fig. S10b. Integrating the measured phase-noise spectrum all the way to $f_{rep}/2$ would hence lead to a result that is dominated by a spectrally white part and is hence unsuited for representing the actual phase noise of the signal. The upper boundary of the integral is hence often set to

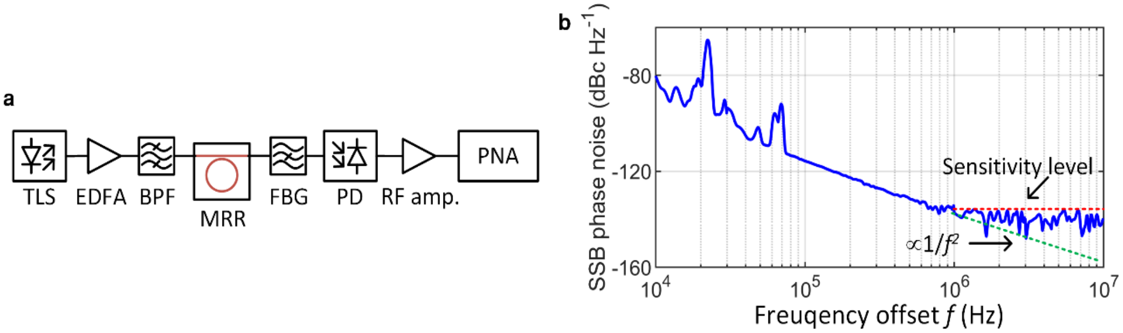

Fig. S10| Characterization of the RF phase noise and timing jitter of our Kerr soliton comb. a Measurement setup. The Kerr soliton comb is generated by pumping the high-Q silicon-nitride (Si_3N_4) microring resonator (MRR) using a tuneable laser source (TLS). The pump tone is amplified by an erbium-doped fibre amplifier (EDFA) and sent through a bandpass filter (BPF) for removing unwanted ASE noise. At the output of the MRR, the strong residual pump tone is suppressed by a fibre Bragg grating (FBG) serving as a notch filter. The comb is then sent to a high-speed photodetector. The resulting RF beat signal passes through an RF amplifier, and the phase noise is extracted by a phase-noise analyser (PNA). **b** Measurement results. The single-sideband (SSB) power spectral density of the phase noise obtained for the fundamental RF beat note of the generated Kerr soliton comb (f_{rep} = 40.025 GHz) is depicted as the blue curve. The red dashed line indicates the sensitivity floor of the PNA for the rather lower power of the RF beat note, which amounts to around −135 dBc Hz^{-1}. The green dashed line indicates a phase noise decay in proportion to $1/f^2$ that would apply to the case of ideal spectrally white frequency noise.

f_2 = 10 MHz [29], assuming that the unavoidable spectrally-white noise background of the measurement does not yet play a significant role, while the true phase-noise components for offset frequencies above f_2 can be neglected. Following this procedure, RMS timing jitter of the LO comb can hence be obtained from the relation

$$\sigma_{\text{LO}} = \frac{1}{2\pi f_{\text{rep}}} \sqrt{2\int_{f_1}^{f_2} \mathcal{L}(f)\, df} \tag{S52}$$

where $\mathcal{L}(f)$ is the SSB phase noise spectrum and where f_{rep} = 40.025 GHz is the repetition rate of the Kerr soliton comb. For our LO comb generator, we find an RMS timing jitter of approximately $\sigma_{\text{LO}} = 3.1\,\text{fs}$, which is already better than the $\sigma_{\text{ADC}} = 25\,\text{fs}$ RMS jitter specified for the electronic clock of our real-time oscilloscopes assuming also an acquisition period of T_{acq} = 10 µs [28].

S10. Power consumption of spectrally sliced photonic-electronic ADC

Power consumption is a key aspect of ADC in general. While our proof-of-concept demonstration discussed in the main manuscript relies on a highly experimental laboratory setup, for which power consumption was not a point of concern, the scheme also lends itself to more efficient implementations using, e.g., high-speed CMOS ADC rather than benchtop-type real-time oscilloscopes. In the following, we estimate the power consumption of a future photonic-electronic ADC similar to the one illustrated in Fig. 1 of the main manuscript, assuming that the implementation fully relies on components that are already available today.

Our estimation starts from a recently published paper [33], which analyses the energy consumption associated with different schemes of photonic-electronic ADC. This analysis is based on the so-called Schreier figure of merit FOM_{Sch} for ADC [34], which relates the sampling frequency f_s and the signal-to-noise-and-distortion ratio (SINAD) of a full-scale test signal to the ADC power consumption P_{ADC},

$$\text{FOM}_{\text{Sch}} = \frac{f_{\text{s}} \times \text{SINAD}}{2P_{\text{ADC}}} \tag{S53}$$

Note that FOM_{Sch} has the unit J^{-1}, and the associated decibel value $\text{FOM}_{\text{Sch,dB}}$ is assigned the unit dB J^{-1},

$$\text{FOM}_{\text{Sch,dB}} = 10\log_{10}\left(\frac{f_{\text{s}} \times \text{SINAD}_{\text{ADC}}}{2P_{\text{ADC}}}\right) \tag{S54}$$

As an example, state-of-the-art electronic ADC based on 5 nm CMOS technology can achieve bandwidths of 60 GHz with sampling rates of 200 GSa/s while offering an $\text{FOM}_{\text{Sch,EADC,dB}}$ of, e.g., 153 dBJ^{-1} [36].

In a next step, we calculate the Schreier FOM that can be expected for an implementation of our photonic-electronic ADC scheme with state-of-the-art components. For a 320 GHz implementation using four spectral slices in the optical domain, we need eight electronic ADC, each offering an acquisition bandwidth of slightly more than 40 GHz. We assume that this can be accomplished with a state-of-the-art 100 GSa/s ADC implementation, having the above-mentioned $\text{FOM}_{\text{Sch,EADC,dB}}$ of 153 dB J^{-1} [36]. We further aim at an effective number of bits of $\text{ENOB}_{\text{PE-ADC}} = 5$ for our photonic-electronic ADC (PE-ADC), corresponding to a $\text{SINAD}_{\text{PE-ADC,dB}}$ of approximately 31.86 dB, see Section S7. Note that these $\text{ENOB}_{\text{PE-ADC}}$ and $\text{SINAD}_{\text{PE-ADC,dB}}$ numbers are common for high-speed ADC [33] and are well within the limits given by the timing jitter of our Kerr comb and by state-of-the-art electric clock oscillators, see Section S11 below. The requirement with respect to the SINAD of the photonic-electronic ADC ($\text{SINAD}_{\text{PE-ADC,dB}}$) can be translated into a corresponding SINAD requirement $\text{SINAD}_{\text{EADC,dB}}$ of the underlying electronic ADC. More specifically, considering a spectrally sliced scheme with N coherent receivers and eight corresponding electronic ADC and assuming that the distortions and noise contributions of the electronic ADC dominate over all other imperfections, the $\text{SINAD}_{\text{PE-ADC}}$ of the photonic-electronic ADC is reduced by a factor of N as compared to the $\text{SINAD}_{\text{EADC}}$ of the electronic ADC,

$$\mathrm{SINAD_{PE-ADC}} = \mathrm{SINAD_{EADC}} / N \tag{S55}$$

This can be understood by considering the fact that the spectrally disjoint noise and distortion contributions in the N spectral slices simply add up in power, while the amplitude of a full-scale signal is still dictated by the optical amplitude that can be detected by a single IQR and digitized by the subsequent pair of ADC. In contrast to that, for the in-phase and quadrature signals detected by each ADC of such a pair, the powers add up, such that the resulting SINAD of a full-scale signal corresponds to the $\mathrm{SINAD_{EADC}}$ of each individual electronic ADC. Note that the decrease of the SINAD with slice count N does not necessarily imply performance degradation of the proposed ADC scheme when it comes to the acquisition of broadband signals. In contrast to the full-scale monochromatic test signals used in the SINAD definition, technically relevant waveforms such as high-speed communication signals usually have broadband spectra with an approximately uniform power spectral density. For such signals, increasing the slice count N does not degrade the highest SNR that can be measured, since additional slices simultaneous increase the system noise power and the maximum signal power that can be acquired by the system.

According to Eq. (S55), the envisaged photonic-electronic ADC implementation with $N = 4$ spectral slices requires electronic ADC with an underlying $\mathrm{SINAD_{EADC}} = 4 \times \mathrm{SINAD_{PE\text{-}ADC}}$, corresponding to $\mathrm{SINAD_{EADC,dB}} = 37.86$ dB. Using this $\mathrm{SINAD_{EADC,dB}}$ value together with the required sampling rate of around $f_s = 100$ GSa/s for a 40 GHz acquisition bandwidth and assuming the above-mentioned Schreier figure-of-merit of $\mathrm{FOM_{Sch,EADC,dB}} = 153$ dB $\mathrm{J^{-1}}$, we can estimate a power consumption of 0.15 W for each of the overall $2N = 8$ hypothetic electronic ADC in our implementation, resulting in a total power of 1.20 W for the entire electronic ADC array. On top of this, we have to consider the power consumption of the photonic front end. To this end, we individually estimate the power dissipation of all the components illustrated in the Fig. 1 of our main manuscript, see Table S1 for an overview of the various numbers. Specifically, we assume four silicon-photonic integrated coherent receivers (ICR) with co-integrated TIA that consume 1.24 W in total [37]. For the external-cavity laser (ECL), which relies on a reflective optical semiconductor amplifier (RSOA) that delivers 20 dBm of saturation output power, was assume a power consumption of approximately 0.40 W [38]. The LO combs as well as the optical carrier fed to the MZM must be amplified, e.g., by semiconductor optical amplifiers (SOA), which consume 0.54 W each, i.e., 1.08 W in total [39]. The optical signal at the output of the MZM should also be amplified, where an erbium-doped waveguide amplifier (EDWA) [43] is preferred over an SOA due to reduced pattern effects and higher signal fidelity. The power consumption of the EDWA can be estimated by assuming a wall-plug efficiency of 33 % for the associated 980 nm pump laser [40], and up to 60 % for pump-to-C-band power conversion efficiency of the EDWA chip [43]. This leads to a power consumption of 0.51 W for 20 dBm of optical output power. The total power consumption of our photonic-electronic ADC implementation hence amounts to 4.43 W. Note that this number does not include the power dissipation of additional digital signal processing (DSP) routines that are used to reconstruct the signal – this power consumption is commonly not considered relevant for the Schreier figure-of-merit [34]. In fact, these DSP routines might be combined with

Components	Power consumption	Reference
8 × 40 GHz ADC	8 × 0.15 W = 1.20 W	[36]
4 × Integrated coherent receiver with TIA	4 × 0.31 W = 1.24 W	[37]
1 × RSOA	0.40 W	[38]
2 × SOA	2 × 0.54 W = 1.08 W	[39]
1 × EDWA	0.51 W	[40],[43]
**Total:**	4.43 W	

Table S1| Estimated power consumption of a photonic-electronic ADC implementation with $N = 4$ spectral slices, offering an overall acquisition bandwidth of 320 GHz. Our analysis follows the scheme illustrated in Fig. 1 of the main manuscript and entirely relies on components that are already available today, see references in the last column. The power consumption of the underlying electronic ADC is estimated based on a Schreier figure of merit of 153 dB $\mathrm{J^{-1}}$ [36] along with a sampling rate of $f_s = 100$ GHz and a SINAD performance that would permit for an overall $\mathrm{ENOB_{PE\text{-}ADC}}$ of 5 ($\mathrm{SINAD_{PE\text{-}ADC,dB}} = 31.86$ dB). The power consumption of the erbium-doped waveguide amplifier (EDWA) is estimated based on the wall plug efficiencies that are provided in [40] and [43]

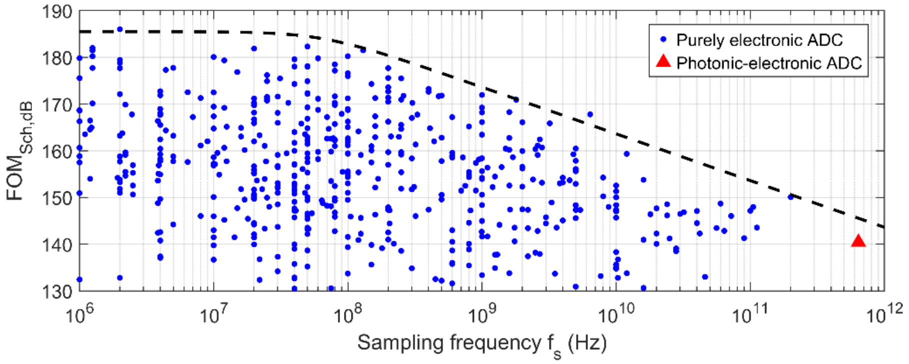

Fig. S11| Schreier figure-of-merit (FOM) as a function of sampling rate. The blue dots represent the Schreier FOM values demonstrated for high-speed all-electronic ADC [41], while the black dashed line indicates an "envelope" of these data points by averaging five data points with the best combination of FOM and sampling frequency. The red marker stands for a realistic future implementation of our photonic-electronic ADC system by using the FOM of electronic ADC demonstrated recently [36].

subsequent application-specific DSP blocks such das fast Fourier transforms (FFT) or inverse FFT (IFFT), see Section S12 for details.

With the total power dissipation of the photonic-electronic ADC at hand, we can now calculate the Schreier figure of merit of our photonic-electronic ADC implementation using Eq. (S54) above. Offering a 320 GHz acquisition bandwidth, the effective sampling rate of our system amounts to at least f_s = 640 GSa/s, leading to a Schreier figure of merit of $\mathrm{FOM_{Sch,EADC,dB}}$ = 140.4 dB $\mathrm{J^{-1}}$. This number compares well to the $\mathrm{FOM_{Sch}}$ values obtained for high-speed all-electronic ADC, which range between 130 dB $\mathrm{J^{-1}}$ and 150 dB $\mathrm{J^{-1}}$ [41] and can also well compete with alternative implementations of photonic-electronic ADC, see [33] for details. For illustration, we have expanded the overview figure given in Ref. [41], see Fig. S11, by including the Schreier figure of merit that we estimated for our photonic-electronic ADC implementation, see red triangle. We conclude that the proposed photonic-electronic ADC concept does not only offer unprecedented bandwidth, but is also amenable to technical implementations with competitive performance in terms of power dissipation and related figures-of-merit, leveraging only optical and electronic components that are already available today.

S11. Impact of electronic and Kerr-comb-related timing jitter

Besides offering large acquisition bandwidth, photonic-electronic ADC can also pave a path towards overcoming the jitter limitations of all-electronic ADC systems, e.g., by using highly stable mode-locked laser as a precise timing reference [26]. When properly stabilized, Kerr combs can offer similar perspectives [27], rendering the proposed photonic-electronic ADC scheme particularly attractive. To estimate the impact jitter on the performance of our system, let us first concentrate on the electronic part and analyse the impact of clock jitter of the electronic ADC that are used to digitize the signal spectral slices. In our experiment, these ADC are implemented by high-speed real-time oscilloscopes (Keysight UXR series), which offer a sampling rate of 256 Gbit/s for each of the overall eight channels. For these devices, the manufacturer specifies a clock timing jitter of 25 fs. On top of this, the photonic-electronic slicing scheme introduces additional timing uncertainties that originate from the uncorrelated part of the optical phase noise associated with the various LO tones. These uncertainties can be quantified through the RF phase-noise spectrum and can be condensed into the simple metric of a root-mean-square (RMS) timing jitter for a given frequency range of interest, see Section S9.2 above.

The combined impact for Kerr-comb timing jitter and clock jitter of the electronic ADC can be quantified by following the approach described in Ref. [31]. Kerr-comb timing jitter and ADC clock are statistically independent, and we can simply calculate the combined distortions by adding the noise and distortion powers of the individual effects. In general, timing jitter leads to distortions with a power spectral density that increases quadratically with frequency. For the electronic ADC, the relevant frequency corresponds to the offset $f_{\mathrm{RF}} - (2\mu - 1) f_{\mathrm{rep}}$ of the RF frequency f_{RF} from

the centre $(2\mu-1) f_{\text{rep}}$ of the respective slice, corresponding to the nearest LO tone in the optical domain. The signal-to-noise-and-distortion ratio $\text{SINAD}_{\text{tj,ADC,dB}}$, that would result if timing jitter of the electronic ADC was the only impairment, thus decreases towards the edge of the respective slice μ and can be written as

$$\text{SINAD}_{\text{tj,ADC,dB}} = 20\log 10\left(\frac{1}{2\pi\cdot\left|f_{\text{RF}}-(2\mu-1) f_{\text{rep}}\right|\cdot\sigma_{\text{ADC}}}\right) \tag{S56}$$

where $f_{\text{RF}} \in \left\lceil 2(\mu-1) f_{\text{rep}}, 2\mu f_{\text{rep}} \right\rceil$. The timing jitter of the optical LO comb adds to these distortions and increases with the spectral offset $2(\mu-1) f_{\text{rep}}$ of the corresponding LO tone at $f_0+(2\mu-1) f_{\text{rep}}$ from the centre pump line at f_0, which was used as an optical carrier for the MZM. The corresponding signal-to-noise-and-distortion ratio can then be written as

$$\text{SINAD}_{\text{tj,LO,dB}} = 20\log 10\left(\frac{1}{(2\mu-1)\cdot 2\pi f_{\text{rep}}\cdot\sigma_{\text{LO}}}\right) \tag{S57}$$

The two effects can be combined by adding the corresponding distortions. The resulting SINAD of a photonic-electronic ADC, that is limited by electronic and Kerr-comb-related timing-jitter effects only, can hence be written as

$$\text{SINAD}_{\text{tj,dB}} = 20\log 10\left(\frac{1}{\sqrt{\left((2\mu-1)\cdot 2\pi f_{\text{rep}}\cdot\sigma_{\text{LO}}\right)^2+\left(2\pi\cdot\left|f_{\text{RF}}-(2\mu-1) f_{\text{rep}}\right|\cdot\sigma_{\text{ADC}}\right)^2}}\right) \tag{S58}$$

where $f_{\text{RF}} \in \left\lceil 2(\mu-1) f_{\text{rep}}, 2\mu f_{\text{rep}} \right\rceil$. Figure S12 shows the individual contributions of the electronic timing jitter (yellow dashed trace) and LO timing jitter (purple dashed trace) as well as the overall $\text{SINAD}_{\text{tj,dB}}$ as a function of RF

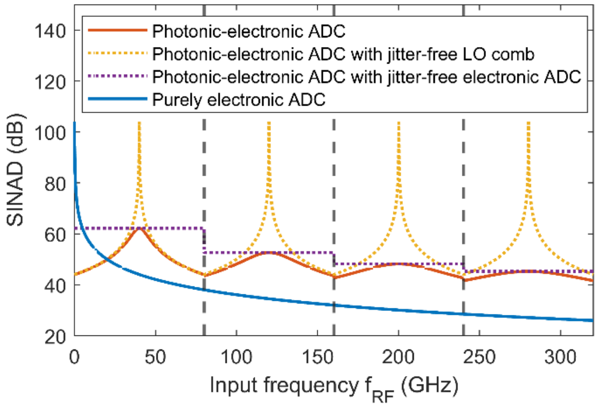

Fig. S12| Jitter-related limitations of the achievable signal-to-noise and distortion (SINAD) ratios for a full-scale sinusoidal test signal as a function of input frequency. We assume a four-slice photonic-electronic ADC with an 80 GHz bandwidth for each slice, resulting in an overall acquisition bandwidth of 320 GHz. The vertical grey dashed lines mark the boundary between neighbouring slices. The dashed yellow traces indicate the individual contributions of the electronic clock jitter originating from the underlying ADC according to Eq. (S53), assuming a clock timing jitter of σ_{ADC} = 25 fs as specified by the manufacturer of our high-speed oscilloscopes. The purple dashed trace indicates the slice-specific contribution of the timing jitter associated with the LO comb, see Eq. (S54). Here, we assumed an LO comb timing jitter of σ_{LO} = 3.1 fs as estimated in Section S9.1. The solid red trace corresponds to the combined effects. As a reference, we also include the jitter-related SINAD limitations of a theoretical 320 GHz purely electronic ADC relying on a clock with a 25 fs timing jitter, see blue trace. We find that the low timing jitter of the LO comb allows the photonic-electronic ADC to provide a jitter-related SINAD limit that is significantly better than that of an all-electronic ADC – besides offering unprecedented bandwidth scalability. Note, however, that our current demonstrations could not yet leverage this advantage since other sources of distortions were still dominant.

frequency f_{RF}. In this plot, we assume a four-slice implementation of a photonic-electronic ADC with an 80 GHz bandwidth for each of the overall four slices, i.e., an overall acquisition bandwidth of 320 GHz, along with an electronic RMS clock jitter of σ_{ADC} = 25 fs as specified by the oscilloscope manufacturer and an RMS comb timing jitter of σ_{LO} = 3.1 fs, as estimated in Section S9.1 above. Figure S12 also shows the jitter-related SINAD limitations that would apply to a theoretical 320 GHz purely electronic ADC, relying on a clock with a 25 fs timing jitter, see blue trace. We find that, due to the low timing jitter of the LO comb, our photonic-electronic scheme can provide a jitter-related SINAD limit that is significantly better than that of an all-electronic ADC – besides allowing for unprecedented bandwidth scalability. Note, however, that our current demonstrations could not yet leverage this advantage since other sources of distortions were still dominant.

S12. Computational complexity of signal reconstruction

When it comes to real-world application of our photonic-electronic ADC, the computational complexity of the signal reconstruction might play an important role. Notably, the digital signal processing (DSP) schemes described in Section S2 above only involve fundamental functions such as fast Fourier transformations (FFT), digital filtering in the frequency domain, and inverse fast Fourier transformations (IFFT). All of these functions are routinely used in high-speed coherent optical communication systems, typically implemented on application specific integrated circuits (ASIC) that are well suited for real-time processing, such that implementation in a compact photonic-electronic ADC should not be a fundamental problem. In the following, we support this notion by quantifying the computational complexity of our digital signal reconstruction techniques and by comparing it to the computational power of state-of-the-art ASIC and field-programmable gate arrays (FPGA).

S12.1. Estimation of computational complexity

Figure S13a shows a graphical illustration of our signal-reconstruction algorithm, based on which we estimate the computational effort in terms of additions and multiplications. Our analysis assumes an implementation with $N = 4$ spectral slices, using $2N = 8$ parallel electronic ADC to produce $2N = 8$ real-valued digital waveforms in the time domain. These samples are initially paired to create four complex-valued data sets, a step that involves only data alignment without arithmetic operations. The DSP process then follows four primary steps that are outlined in the following:

- **Fast Fourier transformation (FFT):** In the first step, an FFT is applied to each data set. This FFT can be efficiently implemented using the so-called overlap-save method, where the incoming time-domain sequence is split into blocks of L_{B} samples with overlaps, where, e.g., the first $L_{\mathrm{B}}/4$ points of each new FFT block are the same as the last $L_{\mathrm{B}}/4$ points of the previous FFT block, see Ref. [50] for details. Choosing a proper number of L_{B} is important to ensure sufficient frequency resolution to account for detailed frequency-dependent characteristics of each of the N detection channels, while maintaining an acceptable computing efficiency. For a raw calibration frequency resolution of 250 MHz in each of our $2N = 8$ detection channels, see Section S5 of SI, the impulse response of the detection channels should not exceed 1/250 MHz = 4 ns. With a minimum required sampling rate of 80 GSa/s for a 40 GHz acquisition bandwidth of each electronic ADC and a raw calibration frequency resolution of 250 MHz four our $2N = 8$ detection channels, see Section S5 of SI, the minimum number of samples per block should be at least $L_{\mathrm{B}} = 320$, to cover the full impulse response of a detection channel. Assuming further a minimum required sampling rate of 80 GSa/s for a 40 GHz acquisition bandwidth of each electronic ADC, the minimum number of samples per block should be at least $L_{\mathrm{B}} = 320$. In our analysis, we assume a much larger block size of $L_{\mathrm{B}} = 2^{13} = 8192$ to be on the safe side, and the overlap of subsequent blocks was set to $L_{\mathrm{OL}} = 2^{11} = 2048$ samples, thus also ensuring that the duration of the overlap exceeds the length of the impulse response of the respective detection channel. According to the split-radix FFT algorithm, as specified in Ref. [51] and Ref. [52], the number real-valued multiplications $M_{\mathrm{FFT},\times}$ required for a single FFT of an L_{B}-point complex-valued waveform

$$M_{\mathrm{FFT},\times} = L_{\mathrm{B}} \log_2\left(L_{\mathrm{B}}\right) - 3L_{\mathrm{B}} + 4 \tag{S59}$$

  while the number $M_{\mathrm{FFT},+}$ of real-valued additions amounts to

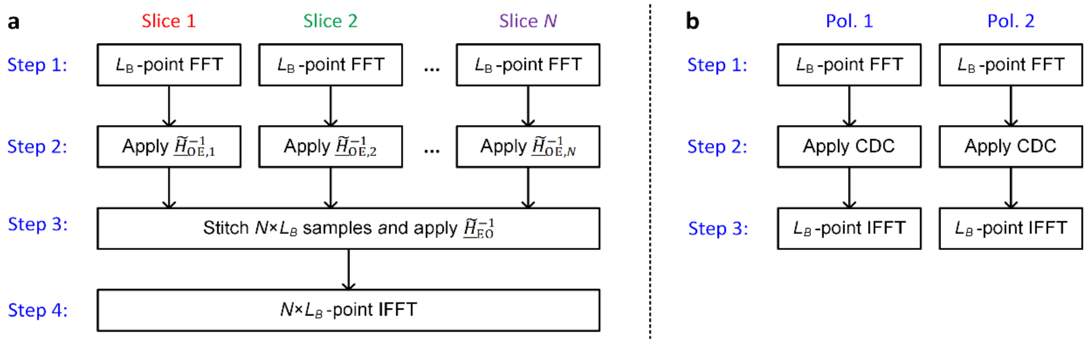

Fig. S13| Graphical illustration of the signal-reconstruction algorithms used in our photonic-electronic ADC and of a bulk chromatic dispersion (BCD) compensation algorithm as used in commercially available long-haul coherent optical transceivers. a Block diagram illustrating the signal-reconstruction algorithm of a spectrally sliced photonic-electronic ADC with N spectral slices and a signal block length of L_B sampling points per slice. The algorithms comprise N fast Fourier transformations (FFT) with L_B complex-valued sampling points for each slice, followed by a compensation of the opto-electronic (OE) transfer function of each slice in the frequency domain. The corrected samples are then stitched to form an N×L_B sequence, to which we apply the compensation of the electro-optic (EO) transfer function. Finally, the reconstructed signal block is obtained by a single N×L_B- inverse FFT (IFFT). **b** Graphical illustration of BCD compensation algorithm used in state-of-the-art long-haul coherent optical transceivers, which requires similar processes. The DSP runs on two polarizations simultaneously, where for each polarization, an L_B-point FFT is performed in a first step. Then the chromatic dispersion compensation (CDC) is applied in the frequency domain, and an L_B-point FFT transforms the signal blocks back to the time domain.

$$M_{\mathrm{FFT},+} = 3L_{\mathrm{B}} \log_2 \left(L_{\mathrm{B}}\right) - 3L_{\mathrm{B}} + 4 \tag{S60}$$

Note that the FFT is performed on all N complex-valued time-domain signal blocks in parallel, i.e., the overall effort for processing all concurrent blocks increases by a factor of N as compared to the values given by Eq. (S59) and (S60).

- **Compensation of the opto-electronic (OE) detection-channel characteristics and signal stitching:** In this step, we compensate for the measured characteristics of each of the N detection channels, see Section S5 of the SI for a more description of the calibration technique. To this end, we multiply the spectrum of the various signal blocks with the corresponding frequency-dependent elements of the pseudo-inverse of the opto-electronic transfer functions $\tilde{\mathbf{H}}'_{\mathrm{OE}}(f)$, see Section S2.2 and Eq. (S27) above in our SI. Assuming that these transfer functions are pre-calibrated and stored as static filter coefficients, the compensation only involves L_B complex-valued multiplications of variables with constants per filter. This translates to $M_{\mathrm{OE},\times}$ real-valued multiplications,

$$M_{\mathrm{OE},\times} = 3L_{\mathrm{B}} \tag{S61}$$

and $M_{\mathrm{OE},+}$ real-valued additions,

$$M_{\mathrm{OE},+} = 3L_{\mathrm{B}} \tag{S62}$$

for each of the N tributary frequency-domain signals. In a next step, the four data sets are stitched by concatenating the N tributary frequency-domain signals of length L_B each, leading to a spectrum of a total of $N \times L_B$ samples for each signal block. This step involves only data alignment without arithmetic operations.

Note that in the experimental implementation based on discrete components, additional operations are needed for compensation of phase drifts, see Section S2.2 above. However, since these phase drifts are slow, the do not need to be re-calculated for each block – it is sufficient to estimate them from time to time and to adapt the slice-specific OE transfer functions accordingly. The associated effort is negligible compared to operations like FFT, IFFT or filtering that need to be performed on each sampling point of the time- or the frequency-domain waveforms.

- **Compensation of the electro-optic (EO) MZM transfer function**: To retrieve the real-valued time-domain waveform with a correct amplitude, we need to correct for the EO transfer function of the MZM and for a residual common amplitude and phase factor, see details in Section S2.2 above. This requires complex-valued

multiplications of a total of $N \times L_B = 4L_B$ samples with static filter coefficients, which translates to $M_{EO,\times}$ real-valued multiplications

$$M_{\mathrm{EO},\times} = 3N \times L_{\mathrm{B}} \tag{S63}$$

and $M_{EO,+}$ real-valued additions

$$M_{\mathrm{EO},+} = 3N \times L_{\mathrm{B}} \tag{S64}$$

Note that the compensation for the EO MZM transfer function could also be merged with the compensation of the opto-electronic (OE) detection-channel characteristics as described in the previous bullet point, thus reducing the computational complexity further. In this sense, our estimate of the computational complexity should be considered to lead to an upper boundary.

- **Inverse FFT (IFFT):** In the last step, an IFFT is performed on the $N \times L_B$ complex-valued frequency-domain samples. This requires $M_{IFFT,\times}$ real-valued multiplications,

$$M_{\mathrm{IFFT},\times} = N \times L_{\mathrm{B}} \log_2\left(N \times L_{\mathrm{B}}\right) - 3N \times L_{\mathrm{B}} + 4 \tag{S65}$$

and $M_{IFFT,+}$ real-valued additions,

$$M_{\mathrm{IFFT},+} = 3N \times L_{\mathrm{B}} \log_2\left(N \times L_{\mathrm{B}}\right) - 3N \times L_{\mathrm{B}} + 4 \tag{S66}$$

Using Eqs. (S59) to (S66), we can now estimate the total number of operations required for signal reconstruction in each of the blocks. For the overall number of real-valued multiplications per block we obtain

$$\begin{aligned} M_{\times} &= N \times M_{\mathrm{FFT},\times} + N \times M_{\mathrm{OE},\times} + M_{\mathrm{EO},\times} + M_{\mathrm{IFFT},\times} \\ &= N \times L_{\mathrm{B}} \log_2\left(L_{\mathrm{B}}\right) + N \times L_{\mathrm{B}} \log_2\left(N \times L_{\mathrm{B}}\right) + 4N + 4 \end{aligned} \tag{S67}$$

whereas the total number of real-valued additions for each block amounts to

$$\begin{aligned} M_{+} &= N \times M_{\mathrm{FFT},+} + N \times M_{\mathrm{OE},+} + M_{\mathrm{EO},+} + M_{\mathrm{IFFT},+} \\ &= 3N \times L_{\mathrm{B}} \log_2\left(L_{\mathrm{B}}\right) + 3N \times L_{\mathrm{B}} \log_2\left(N \times L_{\mathrm{B}}\right) + 4N + 4 \end{aligned} \tag{S68}$$

To further simplify the results, we make use of the fact that real-valued multiplications of variables with constants can be implemented by binary shifts and additions, also referred to the shift-add algorithm [54]. The constant values of the transfer functions can be pre-computed, and the binary shifts can be hard-wired. Assuming the binary samples to be signed values where the most-significant bit (MSB) represents the sign and using a number of N_{bit} bits to represent each real-valued number, each real-valued multiplication can be replaced by a number of α additions, see Ref [53],[54],

$$\alpha = \frac{N_{\mathrm{bit}} - 1}{2} - 1 \quad \text{for } N_{\mathrm{bit}} > 3 \tag{S69}$$

The equivalent total number of additions needed for processing N blocks of L_B complex-valued input samples can hence be estimated as

$$\begin{aligned} M_{\mathrm{total},+} &= \alpha M_{\times} + M_{+} \\ &= (\alpha + 3) N \times L_{\mathrm{B}} \log_2\left(L_{\mathrm{B}}\right) + (\alpha + 3) N \times L_{\mathrm{B}} \log_2\left(N \times L_{\mathrm{B}}\right) + (4\alpha + 4)(N + 1) \end{aligned} \tag{S70}$$

Considering an implementation with $N = 4$ slices and $L_B = 2^{13} = 8192$ complex-valued input samples, and assuming a realistic value of $N_{bit} = 8$, i.e., a multiplication-to-addition conversion factor of $\alpha = 2.5$, the reconstruction of a single block algorithm requires a total number of $M_{total,+} = 5\,046\,342$ additions. We further need to consider that

the overlap-save method requires a total of $N \times L_{\mathrm{OL}}$ samples of the reconstructed time-domain signal need to be discarded since they fall into the overlap region,

$$M_{\mathrm{norm},+} = \frac{M_{\mathrm{total},+}}{N \times \left(L_{\mathrm{B}} - L_{\mathrm{OL}}\right) + 1} \tag{S71}$$

For $L_{\mathrm{OL}} = 2^{11} = 2048$, we obtain a total number $M_{\mathrm{norm},+} = 205$ of real-valued additions for each output sample. In our implementation, to achieve a total acquisition bandwidth of 320 GHz, the output sampling rate will be $f_{\mathrm{s}} = 640$ GSa/s. We can thus estimate the overall operation rate of real-valued additions that is needed for real-time reconstruction of our signals

$$M_{\mathrm{op}} = M_{\mathrm{norm},+} \times f_{\mathrm{s}} = 131 \times 10^{12}\ \text{additions/s} \tag{S72}$$

S12.2. Comparison to state-of-the-art ASIC performance

The operation rate estimated in Section S12.1 can be performed in real-time on a state-of-the-art ASIC. To support this notion, we consider the computational effort to that of bulk chromatic dispersion (BCD) compensation algorithms that are routinely used in long-haul coherent optical transceivers. Specifically, DSP ASIC for 800 Gbit/s coherent communications have been demonstrated on 5 nm CMOS nodes, offering transmission over up to 2000 km using 120 GBd 16QAM in two polarizations [55],[56]. Within the ASIC, BCD DSP is estimated to consume at most 50% of the total DSP resources [55]. Similar to our analysis of the signal reconstruction algorithm for our photonic-electronic ADC in Section S12.1 above, we estimate the number of additions that is needed for each output sample, assuming a DSP chain like the one illustrated in Fig. S13b. The dispersion compensation in the considered DSP ASIC also relies on an overlap-save algorithm with an FFT block size of approximately $L_{\mathrm{B}} = 2^{13} = 8192$ samples, dictated by the dispersion within a 2000 km-long single-mode fibre. In a first step, we analyse the complexity for a single polarization by calculating the numbers $M_{\mathrm{FFT},\times}^{(\mathrm{DC})}$ and $M_{\mathrm{FFT},+}^{(\mathrm{DC})}$ of the multiplications and additions required for the FFT, see Eqs. (S59) and (S60),

$$M_{\mathrm{FFT},\times}^{(\mathrm{DC})} = L_{\mathrm{B}} \log_2\left(L_{\mathrm{B}}\right) - 3L_{\mathrm{B}} + 4 \tag{S73}$$

$$M_{\mathrm{FFT},+}^{(\mathrm{DC})} = 3L_{\mathrm{B}} \log_2\left(L_{\mathrm{B}}\right) - 3L_{\mathrm{B}} + 4 \tag{S74}$$

For chromatic dispersion compensation, frequency-dependent filter coefficients need to be multiplied to the L_{B} complex-valued frequency-domain samples of each block, so the operations $M_{\mathrm{BCD},\times}^{(\mathrm{CD})}$ and $M_{\mathrm{BCD},+}^{(\mathrm{CD})}$ that are required are the same as estimated in Eq. (S61) and (S62)

$$M_{\mathrm{BCD},\times}^{(\mathrm{DC})} = 3L_{\mathrm{B}} \tag{S75}$$

$$M_{\mathrm{BCD},+}^{(\mathrm{DC})} = 3L_{\mathrm{B}} \tag{S76}$$

Subsequently, an IFFT is performed, acting again on L_{B} complex-valued samples. The numbers $M_{\mathrm{IFFT},\times}^{(\mathrm{DC})}$ and $M_{\mathrm{IFFT},+}^{(\mathrm{DC})}$ of real-valued multiplications and additions is then given by

$$M_{\mathrm{IFFT},\times}^{(\mathrm{DC})} = L_{\mathrm{B}} \log_2\left(L_{\mathrm{B}}\right) - 3L_{\mathrm{B}} + 4 \tag{S77}$$

$$M_{\mathrm{IFFT},+}^{(\mathrm{DC})} = 3L_{\mathrm{B}} \log_2\left(L_{\mathrm{B}}\right) - 3L_{\mathrm{B}} + 4 \tag{S78}$$

Converting again real-valued multiplications to additions, see Eq. (S71), we can estimate the total number of additions needed to process one signal block of length of L_{B},

$$\begin{aligned} M^{(\mathrm{DC})}_{\text{single-pol},+} &= \alpha\left(M^{(\mathrm{DC})}_{\mathrm{FFT},\times} + M^{(\mathrm{DC})}_{\mathrm{BCD},\times} + M^{(\mathrm{DC})}_{\mathrm{IFFT},\times}\right) + \left(M^{(\mathrm{DC})}_{\mathrm{FFT},+} + M^{(\mathrm{DC})}_{\mathrm{BCD},+} + M^{(\mathrm{DC})}_{\mathrm{IFFT},+}\right) \\ &= \alpha\left(2L_\mathrm{B}\log_2\left(L_\mathrm{B}\right) - 3L_\mathrm{B} + 8\right) + \left(6L_\mathrm{B}\log_2\left(L_\mathrm{B}\right) - 3L_\mathrm{B} + 8\right) \\ &= \left(2\alpha + 6\right)L_\mathrm{B}\log_2\left(L_\mathrm{B}\right) - \left(3\alpha + 3\right)L_\mathrm{B} + 8\alpha + 8 \end{aligned} \tag{S79}$$

The number of bits of the ADC used in such ASIC chip was not provided, but an $N_\mathrm{bit} = 8$ ($\alpha = 2.5$) is very common in recently published papers on similar ADC chips [35][36]. Using Eq. (S79), we can estimate a total number of $M^{(\mathrm{DC})}_{\text{single-pol},+}$ = 1 085 468 real-valued additions for processing a block of L_B samples for each polarization. Assuming again an overlap of $L^{(\mathrm{DC})}_\mathrm{OL}$ samples, the number of additions per output sample can be written as

$$M^{(\mathrm{DC})}_{\mathrm{norm},+} = \frac{M^{(\mathrm{DC})}_{\text{single-pol},+}}{L_\mathrm{B} - L^{(\mathrm{DC})}_\mathrm{OL} + 1} \tag{S80}$$

Note that the overlap region has to be chosen slightly larger than in the case of the signal reconstruction for the photonic-electronic ADC due to the long impulse response of the dispersive fibre. For 2000 km standard single-mode fibre (SSMF) with a chromatic dispersion coefficient of 20 ps/(nm × km), the accumulated dispersion amounts to 40 000 ps/nm. Reference [58] specifies a 1.4 ns-long dispersion-related impulse-response for an SSMF with an overall dispersion of 1 000 ps/nm, operated at a symbol rate of 95.6 GBd. For the 800 Gbit/s ASIC discussed above, the symbol rate amounts to 120 GBd. Based on these values, we can estimate a value of 70 ns for the length of the dispersion-related impulse response, taking into account that it scales linearly with both the accumulated dispersion and the symbol rate, i.e., the bandwidth of the signal spectrum. Assuming a signal with a root-raised cosine (RRC) pulse shaping and a spectral roll-off of about 10%, an oversampling ratio of at least 1.1 is required to resolve the signal spectrum, leading to a sampling rate of at least 132 GSa/s. The 70 ns-long impulse response hence corresponds to approximately 9200 points, which is already comparable to the FFT block of $L_\mathrm{B} = 2^{13} = 8192$. Therefore, the overlap region has to cover a significant portion of the overall block length, and we assume a value of $L^{(\mathrm{DC})}_\mathrm{OL} = L_\mathrm{B}/2 = 4096$. In this case, the number of additions required per output sample $M^{(\mathrm{DC})}_{\mathrm{norm},+}$ amounts to 265. Note that this analysis only covered the calculational effort for one polarization, while such dispersion compensation has to be performed on both polarizations simultaneously. Therefore, for a sampling rate of f_s = 132 GSa/s, the total rate of real-valued additions amounts to

$$M^{(\mathrm{DC})}_\mathrm{op} = 2 \times M^{(\mathrm{DC})}_{\mathrm{norm},+} \times f_\mathrm{s} = 70 \times 10^{12}\ \text{additions/s} \tag{S81}$$

As stated above, the BCD-compensation DSP typically requires less than 50 % of the ASIC resources [55], i.e., the full capacity of the ASIC should amount to approximately 140×10^{12} addition/s. Such an ASIC should hence be able to handle our fully signal reconstruction algorithm, requiring an overall addition rate of $M_\mathrm{op} = 131 \times 10^{12}$ addition/s, see Eq. (S72). In the future, the performance of such DSP ASIC might further increase, with symbol rates in excess of 200 GBd already being discussed [55].

S12.3. Comparison to state-of-the-art FPGA performance

A challenge of using ASIC for signal reconstruction lies in the fact that many applications do not justify the associated development effort on an advanced CMOS node from a commercial point of view. In this case, an FPGA might be used instead. These devices typically have slightly lower computational powers than ASIC and may thus not be able to perform operations in real time, but may still be a viable option, e.g., for using photonic-electronic ADC in test and measurement equipment. To support this notion, we estimate the processing time needed for reconstructing a 1 ms-long sequence that was captured with a photonic-electronic ADC having $N = 4$ spectral slices and an overall bandwidth of 320 GHz. For such a system, the effective output sampling rate is at least 640 GSa/s, leading to a total number of 0.64 GSa that need to be processed for a 1 ms-long recording. Assuming again a computational effort of 205 real-valued additions per output sample, see Section S12.1 above, we need to perform approximately 131×10^9 additions for signal reconstruction. As a benchmark for FPGA performance, we use a recent demonstration of a 2^{20}-point FFT

with 16 bits per sample that could be processed on an AMD Virtex Ultrascale FPGA within 0.43 ms [57]. The required number of additions for this FFT can be calculated by using Eqs. (S59), (S60) and (S69), and amounts to 39×10^6 additions within 0.43 ms, or, equivalently, 92×10^9 additions per second. Note that this demonstration was far from using the full capacity of the FPGA [57], which might be of the order of hundreds of billions of additions per second. Reconstructing the 1 ms-long recording via 131×10^9 additions might hence be accomplished in less than 1 s, which should be acceptable number for most applications that do not require real-time processing in a dedicated ASIC.

Conflict of interest

M.Z. is co-founder and chief executive officer (CEO) of Hyperlight Corp., a company manufacturing and selling thin-film lithium-niobate high-speed modulators. C.K., D.D., and D.F. are co-founders and shareholders of Teragear GmbH (Karlsruhe, Germany), a company engaged in the development of photonic-electronic signal processing technologies and systems. C. K., T.J.K are co-founders and shareholders of Deeplight GmbH (Karlsruhe, Germany) and Deeplight SA (St. Sulpice, Switzerland), start-up companies commercializing frequency-agile low-noise lasers and frequency comb sources based on Si_3N_4 photonic integrated circuits. The other authors declare no competing interests.

S13. References

[1] Wang, C. et al. Integrated lithium niobate electro-optic modulators operating at CMOS-compatible voltages. Nature 562, 101–104 (2018).

[2] Wang, S. Y. & Lin, S. H. High speed III-V electrooptic waveguide modulators at lambda -1.3 mu m. *Journal of Lightwave Technology* **6**, 758–771 (1988).

[3] Ummethala, S. et al. Hybrid electro-optic modulator combining silicon photonic slot waveguides with high-k radio-frequency slotlines. *Optica* **8**, 511 (2021).

[4] S. Kang et al., "High-efficiency chirped grating couplers on lithium niobate on insulator," Opt. Lett., OL, vol. 45, no. 24, pp. 6651–6654, Dec. 2020, DOI: 10.1364/OL.412902.

[5] Zwickel, H. et al. Verified equivalent-circuit model for slot-waveguide modulators. *Opt. Express*, **28**, 12951–12976 (2020).

[6] M. Burla, C. Hoessbacher, W. Heni et al.; "500 GHz plasmonic Mach-Zehnder modulator enabling sub-THz microwave photonics," APL Photonics 4 (5), 056106 (2019)

[7] W. Freude et al., "High-Performance Modulators Employing Organic Electro-Optic Materials on the Silicon Platform," J. Sel. Top. Quantum Electron. 30(4), 1–22 (2024)

[8] Mertens et al., "Silicon-Organic Hybrid Slot Waveguide Modulators on the Verge of Industrial Adoption," PIC Magazine 3, 18–20 (2024)

[9] Y. Zhang et al., "Systematic investigation of millimeter-wave optic modulation performance in thin-film lithium niobate," *Photon. Res*. **10**, 2380-2387 (2022)

[10] Ummethala, S. et al. Hybrid electro-optic modulator combining silicon photonic slot waveguides with high-k radio-frequency slotlines. Optica 8, 511 (2021).

[11] Fang, D. et al. Optical Arbitrary Waveform Measurement (OAWM) Using Silicon Photonic Slicing Filters. *Journal of Lightwave Technology* 40, 1705–1717 (2022).

[12] Brennan, D. G. Linear diversity combining techniques. *Proceedings of the IEEE* **91**, 331–356 (2003).

[13] P. Maier et al., "Freeform terahertz structures fabricated by multi-photon lithography and metal coating." arXiv, Jan. 06, 2024. DOI: 10.48550/arXiv.2401.03316.

[14] D. J. Kane and R. Trebino, "Characterization of arbitrary femtosecond pulses using frequency-resolved optical gating," *J. Quantum Electron.* **29** (2), 571 (1993)

[15] D. Drayss et al., "Slice-Less Optical Arbitrary Waveform Measurement (OAWM) on a Silicon Photonic Chip," ECOC 2022, Paper We4E.6 (2022)

[16] D. Fang et al., "Spectrally Sliced Optical Arbitrary Waveform Measurement (OAWM) Using a Photonic Multi-Chip Receiver Assembly," OFC 2024, Paper Tu2A.4 (2024)

[17] D. Drayss et al., "Integrated Non-sliced OAWM Engine Enabling 320 GHz Photonic-Electronic Analog-to-Digital Conversion," OFC 2024, Paper W3B.4 (2024)

[18] IEEE Standard for Terminology and Test Methods for Analog-to-Digital Converters. IEEE Std 1241-2023 1–98 (2023)

[19] The Effective Number of Bits (ENOB) of my R&S Digital Oscilloscope. https://scdn.rohde-schwarz.com/ur/pws/dl_downloads/dl_application/application_notes/1er03/ENOB_Technical_Paper_1ER03_1e.pdf (2011)

[20] Tessmann, A. et al. 243 GHz low-noise amplifier MMICs and modules based on metamorphic HEMT technology. *Int. J. Microw. Wireless Technol.* **6**, 215–223 (2014).

[21] Inoue, K., Kominato, T. & Toba, H. Tunable gain equalization using a Mach-Zehnder optical filter in multistage fiber amplifiers. *IEEE Photonics Technology Letters* **3**, 718–720 (1991).

[22] Hanke Feng, Ke Zhang, Wenzhao Sun, Yangming Ren, Yiwen Zhang, Wenfu Zhang, and Cheng Wang, "Ultra-high-linearity integrated lithium niobate electro-optic modulators," Photon. Res. 10, 2366-2373 (2022)

[23] S. Fujii, and T. Tanabe, “Dispersion engineering and measurement of whispering gallery mode micro resonator for Kerr frequency comb generation”, Nanophotonics, 9(5), pp. 1087-1104, (2020). https://doi.org/10.1515/nanoph-2019-0497
[24] M. Gao, Q.-F. Yang, Q. Ji, et al. “Probing material absorption and optical nonlinearity of integrated photonic materials”, Nat. Commun. 13, 3323 (2022). https://doi.org/10.1038/s41467-022-30966-5
[25] T. J. Kippenberg, A. L. Gaeta, M. Lipson, M. L. Gorodetsky, “Dissipative Kerr solitons in optical microresonators”, Science, 361, eaan8083 (2018). DOI: 10.1126/science.aan8083
[26] Khilo, A. et al. Photonic ADC: overcoming the bottleneck of electronic jitter. Opt. Express 20, 4454–4469 (2012).
[27] Liu, J., Lucas, E., Raja, A.S. et al. Photonic microwave generation in the X- and K-band using integrated soliton microcombs. Nat. Photonics 14, 486–491 (2020).
[28] Keysight Technologies, Datasheet: Infiniium UXR-Series Oscilloscopes, https://www.keysight.com/de/de/assets/7018-06242/data-sheets/5992-3132.pdf. (Accessed Nov. 2023)
[29] Khilo, A. *et al.* Photonic ADC: overcoming the bottleneck of electronic jitter. Opt. Express 20, 4454–4469 (2012).
[30] D. Drayss, D. Fang, C. Füllner, W. Freude, S. Randel, and C. Koos, “Non-sliced Optical Arbitrary Waveform Measurement (OAWM) Using a Silicon Photonic Receiver Chip,” Journal of Lightwave Technology, pp. 1–19, 2024, DOI: 10.1109/JLT.2024.3378994.
[31] Zazzi, A. et al. Fundamental limitations of spectrally-sliced optically enabled data converters arising from MLL timing jitter. Opt. Express 28, 18790–18813 (2020).
[32] R&S FSWP phase noise analyzer and VCO tester, https://scdn.rohdeschwarz.com/ur/pws/dl_downloads/pdm/cl_brochures_and_datasheets/specifications/3683_7719_22/FSWP_specs_en_3683-7719-22_v0500.pdf (Accessed December 2023)
[33] C. Deakin and Z. Liu, “Energy Efficiency Bounds for Photonic Analog to Digital Converters,” J. Lightwave Technol., vol. 42, no. 6, pp. 1819–1828, Mar. 2024, doi: 10.1109/JLT.2023.3328317.
[34] S. Pavan, R. Schreier, and G. C. Temes, Understanding Delta-Sigma Data Converters. IEEE Press, 2017
[35] G. Li et al., “18.1 A 600Gb/s DP-QAM64 Coherent Optical Transceiver Frontend with 4x105GS/s 8b ADC/DAC in 16nm CMOS,” IEEE International Solid-State Circuits Conference (ISSCC), pp. 338-340 (2024)
[36] R. L. Nguyen et al., “18.4 A 200GS/s 8b 20fJ/c-s Receiver with >60GHz AFE Bandwidth for 800Gb/s Optical Coherent Communications in 5nm FinFET,” IEEE International Solid-State Circuits Conference (ISSCC), pp. 344-346 (2024)
[37] J. Verbist et al., “A 40-GBd QPSK/16-QAM Integrated Silicon Coherent Receiver,” IEEE Photonics Technology Letters, vol. 28, no. 19, pp. 2070–2073, Oct. 2016, doi: 10.1109/LPT.2016.2582799.
[38] High power InP RSOA from Fraunhofer HHI https://www.hhi.fraunhofer.de/fileadmin/PDF/PC/LAS/High_Power_InP_DFB_Laser_V2a.pdf (Accessed in Nov. 2024)
[39] Kasper Van Gasse, Ruijun Wang, and Gunther Roelkens, "27 dB gain III–V-on-silicon semiconductor optical amplifier with > 17 dBm output power," Opt. Express 27, 293-302 (2019)
[40] P. Salet et al., “Spectral beam combining of a single-mode 980-nm laser array for pumping of erbium-doped fiber amplifiers,” IEEE Photonics Technology Letters, vol. 17, no. 4, pp. 738–740, Apr. 2005
[41] B. Murmann, “ADC Performance Survey 1997-2024.” [Online]. Available: https://github.com/bmurmann/ADC-survey.
[42] Keysight Technologies, Infiniium UXR-Series Oscilloscopes. https://www.keysight.com/us/en/assets/7018-06242/data-sheets/5992-3132.pdf (accessed 8 June 2024)
[43] Yang Liu et al., “A photonic integrated circuit–based erbium-doped amplifier,” Science 376,1309-1313(2022).
[44] Marin-Palomo, P., Kemal, J., Karpov, M. et al. Microresonator-based solitons for massively parallel coherent optical communications. Nature 546, 274–279 (2017).
[45] Ó.B. Helgason, M. Girardi, Z. Ye et al. Surpassing the nonlinear conversion efficiency of soliton microcombs. Nat. Photon. 17, 992–999 (2023)

[46] C. Bao et al. Nonlinear conversion efficiency in Kerr frequency comb generation. Opt. Lett. 39, 6126–6129 (2014)
[47] E. Gasmi et al., Bandwidth and conversion-efficiency analysis of Kerr soliton combs in dual-pumped resonators with anomalous dispersion, Phys. Rev. A 108, 023505 (2023)
[48] A. Rizzo, A. Novick, V. Gopal et al. Massively scalable Kerr comb-driven silicon photonic link. Nat. Photon. 17, 781–790 (2023)
[49] J. Zang et al., Laser-power consumption of soliton formation in a bidirectional Kerr resonator, arXiv:2401.16740v1 (2024)
[50] Xu, T et al., “Frequency-Domain Chromatic Dispersion Equalization Using Overlap-Add Methods in Coherent Optical System” Journal of Optical Communications, vol. 32, no. 2, pp. 131-135 (2011).
[51] R. Yavne, “An economical method for calculating the discrete Fourier transform,” in Proceedings of the December 9-11, 1968, fall joint computer conference, part I, in AFIPS ’68 (Fall, part I). New York, NY, USA: Association for Computing Machinery, pp. 115–125 (1968)
[52] P. Duhamel and H. Hollman. Split-radix fft algorithms. Electronics Letters 20, pp. 14-16, (1984)
[53] U meyer-baese, Digital Signal Processing with Field Programmable Gate Arrays, 4th edition. Springer Publishing Company (2014).
[54] P. Matalla et al., “Joint Blind Clock Recovery for Space-Division Multiplexed Optical Transmission Systems” submitted to JLT (2024)
[55] S. H. Fan et al., “Toward 1.6T Low-Power Coherent DSP: Challenges, and Lessons Learned from Preceding Generations,” in Optical Fiber Communications Conference (OFC), paper M2H.1, and the associated Oral Presentation: https://opg.optica.org/abstract.cfm?uri=ofc-2024-M2H.1&origin=search#videoPlayer (2024)
[56] Marvell COLORZ 800 pluggable digital coherent optics transceiver. https://www.marvell.com/content/dam/marvell/en/public-collateral/optical-modules/marvell-optical-module-colorz-800-zr-zr+-product-brief.pdf (Accessed in Nov. 2024)
[57] H. Kanders et al., “A 1 Million-Point FFT on a Single FPGA,” in IEEE Transactions on Circuits and Systems, vol. 66, no. 10, pp. 3863-3873, (2019)
[58] H. Sun et al., "800G DSP ASIC Design Using Probabilistic Shaping and Digital Sub-Carrier Multiplexing," in Journal of Lightwave Technology, vol. 38, no. 17, pp. 4744-4756, 1 Sept.1, 2020
